# A pharmacological modality to sequester homomeric proteins

**DOI:** 10.1038/s41589-026-02141-0

**Published:** 2026-02-05

**Authors:** Ella Livnah, Ohad Suss, Adi Rogel, Atar Gilat, Yuval Abdan, José A. Villegas, Ronen Gabizon, Almog Nadir, Yoav Shamir, Noam Y. Steinman, Barr Tivon, Shira Albeck, Tamar Unger, Ofra Golani, Inna Goliand, Nadav Elad, Silvia Carvalho, Khriesto Shurrush, Haim Barr, David Margulies, Emmanuel D. Levy, Nir London

**Affiliations:** 1 Department of Chemical and Structural Biology, Weizmann Institute of Science, Rehovot, Israel; 2 Structural Proteomics Unit, Department of Life Sciences Core Facilities, Weizmann Institute of Science, Rehovot, Israel; 3 Department of Life Sciences Core Facilities, Weizmann Institute of Science, Rehovot, Israel; 4 Department of Chemical Research Support, Weizmann Institute of Science, Rehovot, Israel; 5 Maurice and Vivienne Wohl Institute for Drug Discovery, Nancy and Stephen Grand Israel National Center for Personalized Medicine, The Weizmann Institute of Science, Rehovot, Israel; 6 Department of Molecular and Cellular Biology, University of Geneva, Geneva, Switzerland; 7 Present Address: Department of Pharmaceutical Sciences, College of Pharmacy, University of Illinois Chicago, Chicago, IL USA

**Keywords:** Small molecules, Medicinal chemistry, Pharmacology

## Abstract

Molecules that facilitate protein–protein interactions are immensely impactful. However, such compounds typically rely on accessory proteins to function, such as E3 ligases for targeted degradation, which may restrict their scope or lead to resistance. We alleviate the need for accessory proteins with a strategy that exploits protein symmetry as a selective vulnerability and is widely applicable because of the ubiquitous nature of homomeric proteins. We target homomeric proteins with PINCHs (polymerization-inducing chimeras)—bifunctional molecules composed of two linked ligands that bridge homomers and trigger their supramolecular assembly into insoluble polymers. We design PINCHs that achieve efficient polymerization of four targets. In cells, we observed that a PINCH targeting Keap1 exhibited a longer duration of action and a PINCH targeting BCL6 displayed selective lowering of B cell viability compared to their monomeric parents. Our results highlight PINCHs as a novel and general strategy to modulate and knock out protein function.

**Figure:**
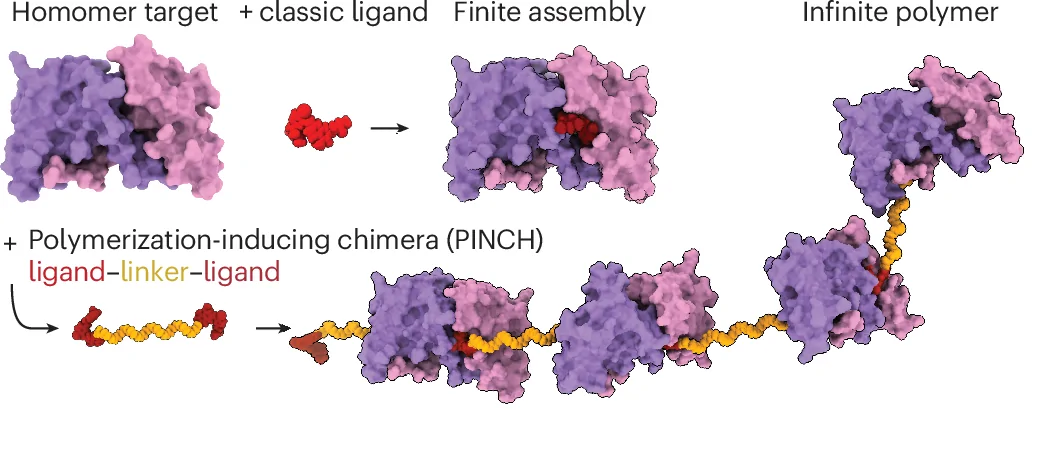

## Abstract (web summary)

Polymerization-inducing chimeras are a novel bifunctional modality that uses protein symmetry to sequester targets by forming insoluble supramolecular assemblies. This approach overcomes reliance on accessory proteins and differs functionally from conventional inhibitors.

## Main

In recent years, the induction of proximity between proteins^1–5^ has become a prevalent approach in chemical biology and drug discovery^6^. A number of modalities use bifunctional small molecules that bind a target protein on one end and ‘hijack’ a cellular effector on the other end to induce proximity between the two proteins and achieve a desired post-translational modification (PTM) on the target. Prominent examples include proteolysis-targeting chimeras (PROTACs) to induce proteasomal degradation^7,8^, lysosome-targeting chimeras to induce lysosomal degradation^9^, phosphorylation-inducing chimeras to induce phosphorylation^10^, deubiquitinase-targeting chimeras^11^ to induce stabilization through deubiqitination and regulated induced proximity-targeting chimeras^12^ to selectively target essential proteins. Monovalent proximity inducers, also known as molecular glues, are also gaining prominence^13,14^.

While targeted degradation by PROTACs offers several advantages over traditional inhibition^15^, these approaches also have a number of drawbacks. The bifunctional molecule has to bind two different molecular targets, which requires, first and foremost, ligands for both proteins and for them to be coexpressed and colocalized in the relevant cellular compartment. In addition, some PTMs have spatial restrictions; thus, solely inducing proximity does not ensure successful modification or degradation of the target^16^. For example, PROTAC-induced or molecular-glue-induced degradation of the target requires a correct positioning of the different components in such a way that there is an available lysine on the surface of the protein for the E2 unit to ubiquitinate^8,17^. From a therapeutic perspective, resistance often stems from the recruited effector^18^. Despite the advances in proximity-based technologies to target proteins of interest^19^, a major challenge remains in expanding their use in a general way. There is a growing need for methods that can be rationally applied to a large range of proteins, minimizing the lengthy screening campaigns that are usually required for each new protein target.

Protein symmetry has long been leveraged to engineer molecular assemblies^20–23^. Recent work has suggested that small chemical changes at the surface of proteins can induce their polymerization, provided that the protein target naturally forms a symmetric complex or homomer^24–27^. The robustness of this approach suggested that proteins are naturally ‘on the verge of’ oligomerization and formation of higher-order protein assemblies. Notably, monovalent small-molecule modulators of a target’s oligomerization state have been mostly discovered serendipitously^28^, with their mode of action typically discovered separately from their functional effect. One example is trifluoroperazine, initially discovered as an inhibitor of S100A4, a member of the S100 protein family that is associated with several human diseases^29^. This compound was eventually revealed to inhibit S100A4’s activity by stabilizing an inactive pentameric complex of it. Another example is a monomeric degrader of the BCL6 transcription repressor^30^, which was shown to polymerize the BTB domain of BCL6 and led to its proteasomal degradation. Recently, Diaz et al. reported a rationally designed trivalent inhibitor designed to capture three units of the lipoprotein(a) K_IV_ domain in an inactive trimer^19^.

**Fig. 1:**
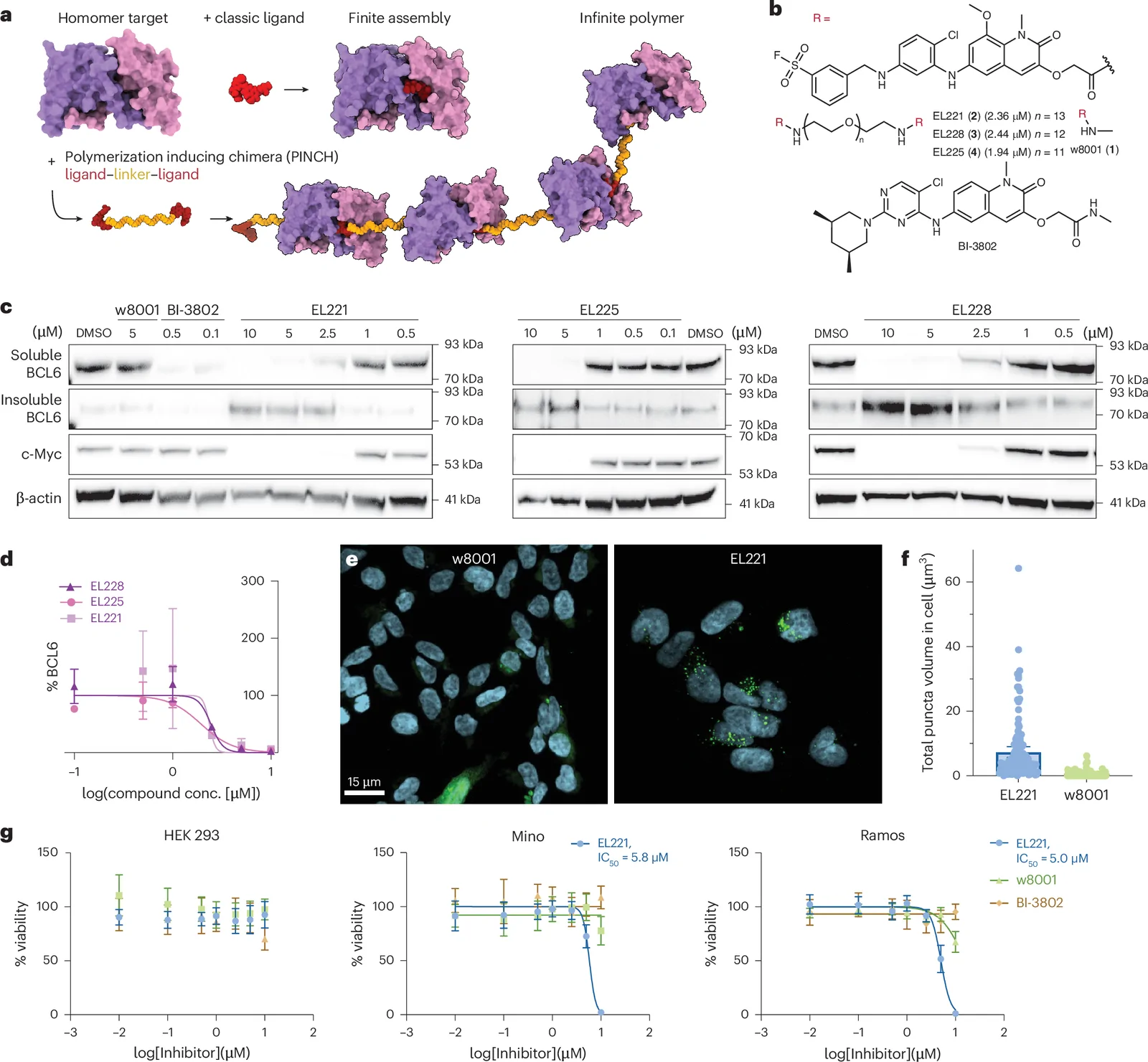
PINCHs precipitate BCL6 in cells and show cell-type-specific viability effects. **a**, Schematic representation of the PINCH mode of action. **b**, Structures of monovalent ligands and PINCHs that target BCL6. EC_50_ values are shown in parentheses. **c**, WB analyses of active BCL6-targeting PINCHs, after 20 h of treatment in Mino cells. Representative results from three independent experiments are shown. Note that we follow the mass of the monomeric BCL6 in WB as, under the harsh sample preparation conditions, the EL221 crosslinked dimer dissociates (Supplementary Fig. 1f–h). **d**, WB band quantification from *n* = 3 independent experiments. Data depict the mean and s.d. EC_50_ curves for individual compounds are shown in Supplementary Fig. 3a. Conc., concentration. **e**, HEK 293 cells overexpressing BCL6(1–275)–GFP treated with 5 μM parent ligand w8001 (left) and 5 μM EL221 (right). **f**, Quantification of BCL6–GFP puncta in each treatment. *P* < 0.0001 in a two-tailed Mann–Whitney test from 223 cells analyzed in two independent experiments. Additional images are shown in Supplementary Fig. 1a. One outlier was removed from the graph for clarity but not from the statistical analysis. Data show the mean values and a 95% confidence interval. **g**, Viability of different cell lines after 72 h of treatment with EL221, parent ligand w8001 or BCL6-polymerizer BI-3802. Representative results from two independent experimental replicates are shown (*n* = 6 technical replicates each). Data depict the mean and s.d. Source data

Here, we rationally design small molecules that can take advantage of the properties of homomeric proteins to efficiently perturb their function. We aimed to achieve this by linking two identical target ligands with a flexible linker. A key design principle is that the bifunctional ligand should not bind two subunits of the same homomer simultaneously, as this would form a closed, finite complex. Instead, if each ligand moiety binds a subunit of a separate homomer, it would drive the assembly of an extended polymeric structure. This process effectively sequesters the protein in a polymeric state that can inhibit its normal function (Fig. 1a). Importantly, as protein homomers are common in nature, making up 25–50% of proteomes and exhibiting a variety of functionalities^31,32^, such an approach is expected to have a wide scope and be generally applicable.

We term these compounds PINCHs (polymerization-inducing chimeras) and present them in this work as a new modality for protein sequestration. We apply this approach to four target proteins: the homodimers BCL6, Keap1 and NQO2 and the homotetramer LDHA. We show that PINCHs can induce polymerization in vitro and in cells and precipitate the target proteins from the soluble phase to the insoluble phase. We show that this polymerization is phenotypically different from inhibition or even degradation and can complement current approaches to perturb protein functions in cells.

## Results

### PINCH-mediated accumulation of BCL6 in the insoluble phase

To evaluate the concept of PINCH as a novel modality, we initially focused on BCL6. The protein BCL6 is a homodimeric transcriptional repressor involved in B cell regulation and its overexpression is correlated with several B cell non-Hodgkin lymphomas^33,34^. Thus, a pharmacological modality to knock down BCL6 is therapeutically relevant and makes it an excellent model system to test the PINCH approach. Several BCL6 inhibitors and degraders have been reported in recent years^35–37^. In addition, a BCL6 PROTAC that is currently under clinical trials demonstrated efficacy in multiple preclinical models of diffuse large B cell lymphoma^38^. Recently, BCL6 was also recruited by a new modality of bifunctional molecules to selectively repress alternative target genes^39,40^. Furthermore, it was discovered that BCL6 was able to polymerize using a small-molecular glue^30^.

**Extended Data Fig. 1:**
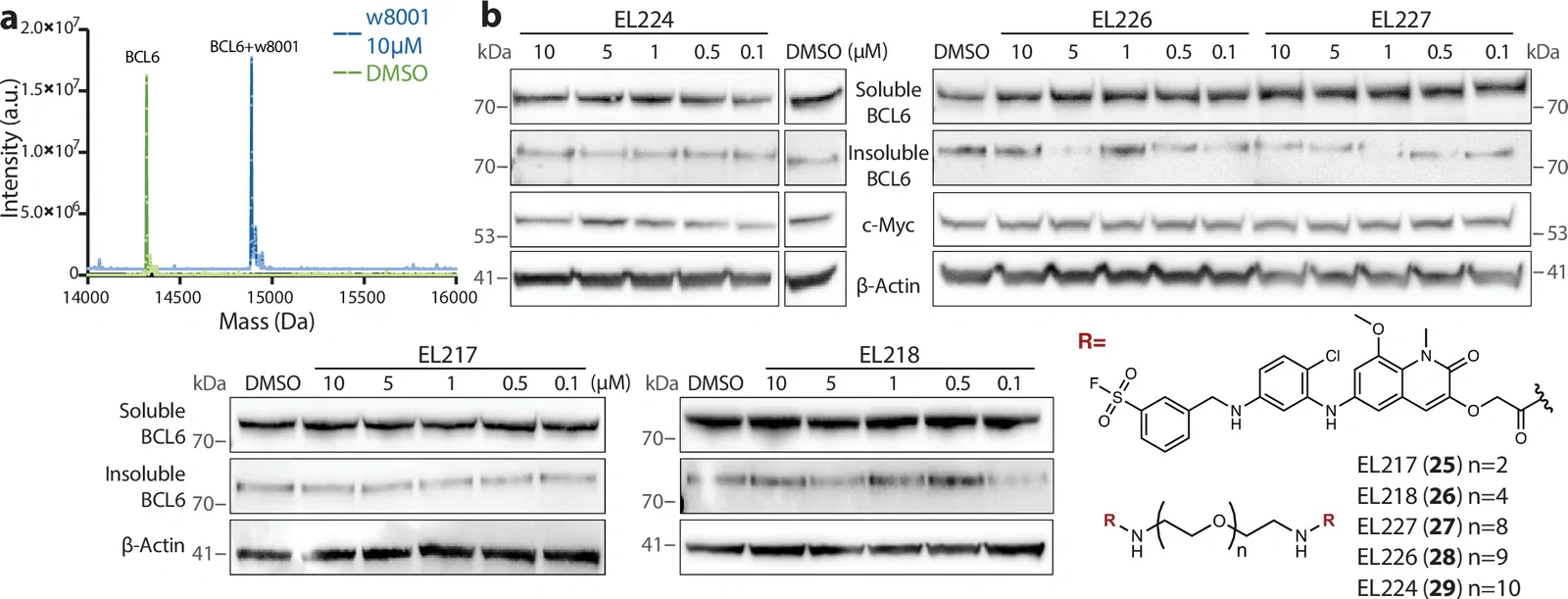
SAR of BCL6 targeting PINCHs. **a**. Intact LC/MS analysis of 2 μM BTB-BCL6 after incubation with DMSO/w8001 for 90 min at room temperature. **b**. WBs of inactive BCL6-targeting PINCHs in Mino cells after 20 h treatments. Representative results from 3 independent experiments, and **c**. These compounds’ structures. Source data

A covalent inhibitor of BCL6 that irreversibly binds a tyrosine residue in the BTB domain dimeric interface was previously reported^41^. We used a close analog of this ligand, w8001 (**1**; Fig. 1b), which showed rapid binding to recombinant BCL6 BTB domain by intact protein liquid chromatography–mass spectrometry (LC–MS) (100% labeling at 2 μM protein and 10 μM compound; incubation time, 90 min; Extended Data Fig. 1a). To create a PINCH targeting BCL6, we synthesized compound EL221 (**2**; Fig. 1b) composed of two w8001 moieties linked by a PEG13 linker. Importantly, the linker had to be long enough to minimize potential steric clashes between bridged homodimers yet short enough to forbid intramolecular binding of subunits from the same homodimer.

We tested the effects of EL221 on BCL6 levels by western blot (WB) in Mino cells, as BCL6 is mostly expressed in B cells. At concentrations starting from 5 μM and after 20 h of incubation, we observed a reduction in BCL6 in the soluble fraction and an accumulation in the insoluble fraction (Fig. 1c). By contrast, the reported BCL6-polymerizing agent BI-3802 (ref. ^30^) showed no accumulation in the insoluble fraction because of proteasomal degradation^7^.

### BCL6 PINCHs functionally differ from their parent inhibitor

To assess the downstream effect of the PINCH-induced polymerization, we monitored the expression of c-Myc (MYC proto-oncogene, bHLH transcription factor), a prominent target regulated by BCL6 (ref. ^42^). In the presence of EL221, BCL6 moved to the insoluble phase and c-Myc protein levels were markedly downregulated, suggesting that EL221 may hinder BCL6 cellular functions (Fig. 1c). Surprisingly, although BI-3802 downregulates BCL6, it showed no such effect on c-Myc, underscoring a potential difference in the mechanism of action of these modalities. To further examine the possibility of EL221-induced c-Myc gene downregulation, we followed its mRNA levels in Mino cells after PINCH treatment. Quantitative PCR (qPCR) analysis did not reveal a difference in c-Myc mRNA levels, implying that the reduction observed by WB does not originate in transcriptional regulation (Supplementary Fig. 1b).

Proteomic analysis of EL221-treated cells revealed many additional downregulated proteins (Supplementary Fig. 1d). We attribute this large and systemic effect to the centrality of BCL6 to B cell biology and the long incubation period (20 h).

Theoretically, using shorter linkers^43–45^ or a different linker chemistry in bifunctional molecules may affect the rigidity of the compound and its interactions with the protein, which could modulate the binding affinity. This motivated us to evaluate how changing the length of the PEG-based linker bridging the two binding moieties impacted the PINCH properties. We observed that the linker could be as short as 11 ethylene glycol units, as for EL225 (**4**; Fig. 1b) but not less (Fig. 1b and Extended Data Fig. 1b,c). The similar potencies we observed for the longer linkers suggest that the PINCHs we examined were not stabilizing a new BCL6–BCL6 interface but rather acted as simple bridges.

To further characterize whether EL221 induces the polymerization of BCL6, we visualized its cellular distribution following treatment in HEK 293 cells expressing GFP fused to BCL6 (residues 1–275; HEK 293 cells were used for ease of transfection). As cells were treated with EL221, the GFP signal accumulated into punctate structures, indicating that BCL6 formed higher-order assemblies. As a control, cells treated with the parent monomeric ligand (w8001) showed no such puncta. Instead, we observed a faint GFP signal spread throughout the cell (Fig. 1e). Indeed, the GFP puncta were significantly overrepresented in EL221-treated cells compared to the monomeric ligand w8001 (*P* < 0.0001; Fig. 1f). An additional experiment comparing active PINCH EL225 (**4**; Fig. 1b) and inactive PINCH EL226 (**28**; Extended Data Fig. 1c), consisting of equivalent concentration of the electrophilic warhead, showed similar significant differences in GFP puncta formation (Supplementary Fig. 1c).

Lastly, given that c-Myc is essential for B cell viability, the marked downregulation in c-Myc levels following PINCH treatment (Fig. 1c) prompted us to evaluate their effect on cellular viability. Comparing active PINCHs EL221 and EL225 and inactive PINCHs EL226 and EL218 (**26**; Extended Data Fig. 1c), as well as w8001 and BI-3802, we observed that no compound affected the viability of HEK 293 cells, which lack BCL6 expression. However, in Mino and Ramos B cells, both of which express BCL6, EL221 and EL225 showed half-maximal inhibitory concentration (IC_50_) values of 5.8 μM and 5.0 μM, respectively, while neither inactive PINCHs EL226 and EL218 nor the monovalent degrader and inhibitor showed any visible effects (Fig. 1g and Supplementary Fig. 1e). These results are in line with another report showing that BI-3802 exhibits viability effects on longer timescales^46^.

### Targeting Keap1 with the PINCH approach

**Fig. 2:**
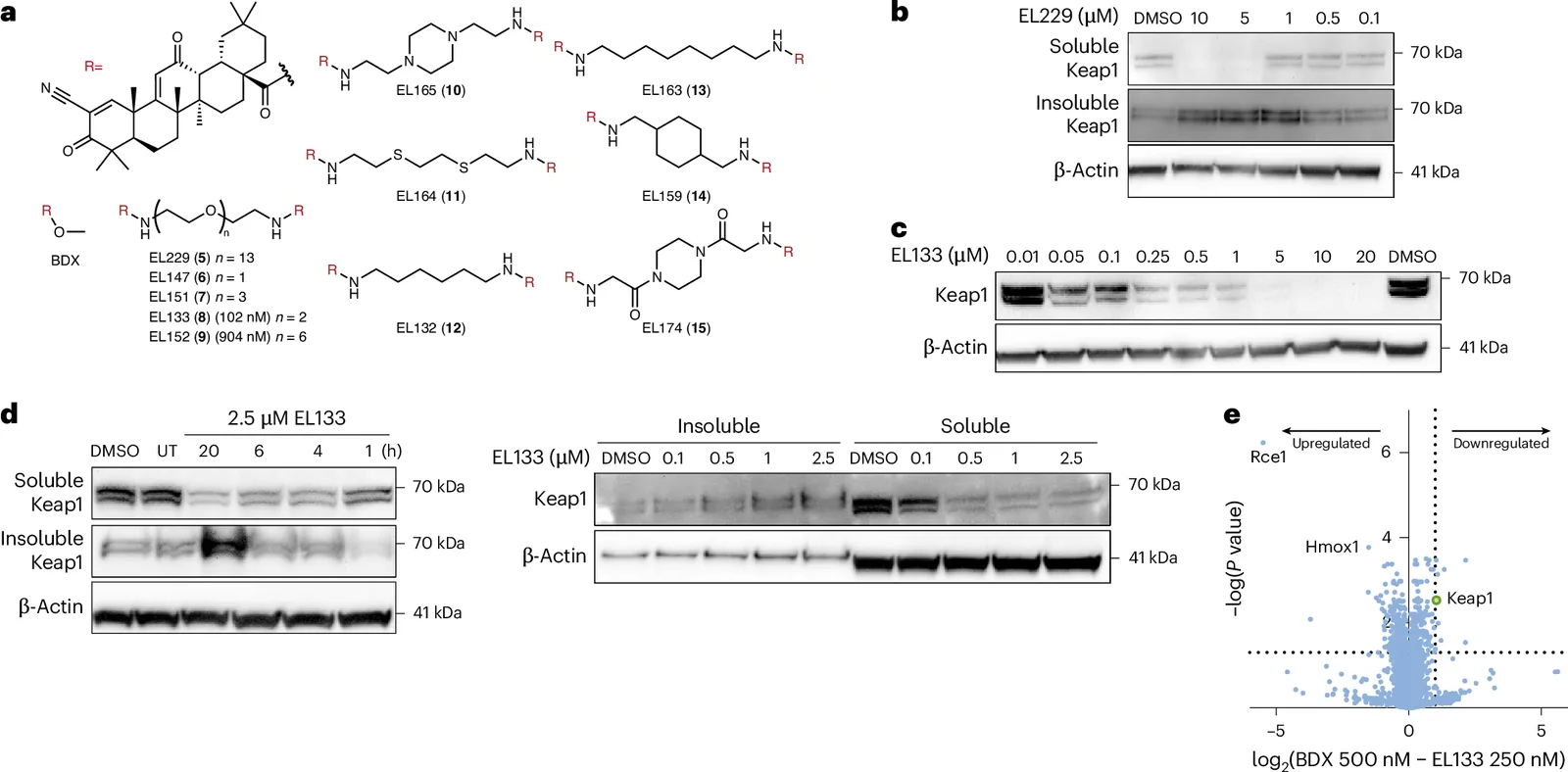
Keap1 PINCHs precipitate Keap1 in cells. **a**, Chemical structures of Keap1-targeting PINCHs. EC_50_ values for the active compounds are shown in parentheses. **b**, WB of EL229’s effect on Keap1 in the soluble and insoluble phases of OCI-AML2 cells (20 h of treatment). Representative results from two independent experiments are shown. **c**, WB of EL133’s effect on soluble Keap1 in OCI-AML2 (20 h of treatment). Representative results from two independent experiments are shown. **d**, EL133 affects Keap1 in the soluble and insoluble OCI-AML2 cell phases, in a time-dependent and dose-dependent manner. WB band quantification from three independent experiments and EC_50_ curves for individual compounds are shown in Supplementary Fig. 3b. UT, untreated. **e**, Global proteomics analysis of the soluble lysate of U2OS cells, showing significant downregulation of Keap1 following treatment with EL133 (250 nM, 20 h) compared to an equivalent amount of BDX (500 nM). The dotted lines mark a twofold reduction in protein level (vertical) and *P* = 0.05 significance in a double-sided Student’s *t*-test (horizontal). Source data

To assess the broader applicability of the PINCH approach, we examined an additional system: the protein Keap1, a substrate adaptor for the E3 ubiquitin ligase complex Cul3–Rbx1, which has a pivotal role in regulating NFE2-like bZIP transcription factor 2 (Nrf2)^47–49^. Nrf2 serves as a master regulator of the cellular anti-oxidative response, and proper functioning of the Keap1–Nrf2 pathway is essential in various diseases characterized by oxidative stress and inflammation^48,50,51^. Similarly to BCL6, Keap1 contains a BTB domain that mediates its homodimerization. Bardoxolone (BDX) is a reversible covalent binder of C151 in the BTB domain of Keap1 and is known to activate the Nrf2 pathway^52,53^. We, therefore, designed a PINCH targeting Keap1 based on BDX, two units of which were coupled by a PEG13 linker, producing EL229 (**5**; Fig. 2a). We tested the effect of EL229 by WB in OCI-AML2 cells, a hematopoietic cell line with stable Keap1 expression, and showed that it reduced Keap1 in the cell’s soluble fraction and induced its accumulation in the insoluble fraction (Fig. 2b).

**Extended Data Fig. 2:**
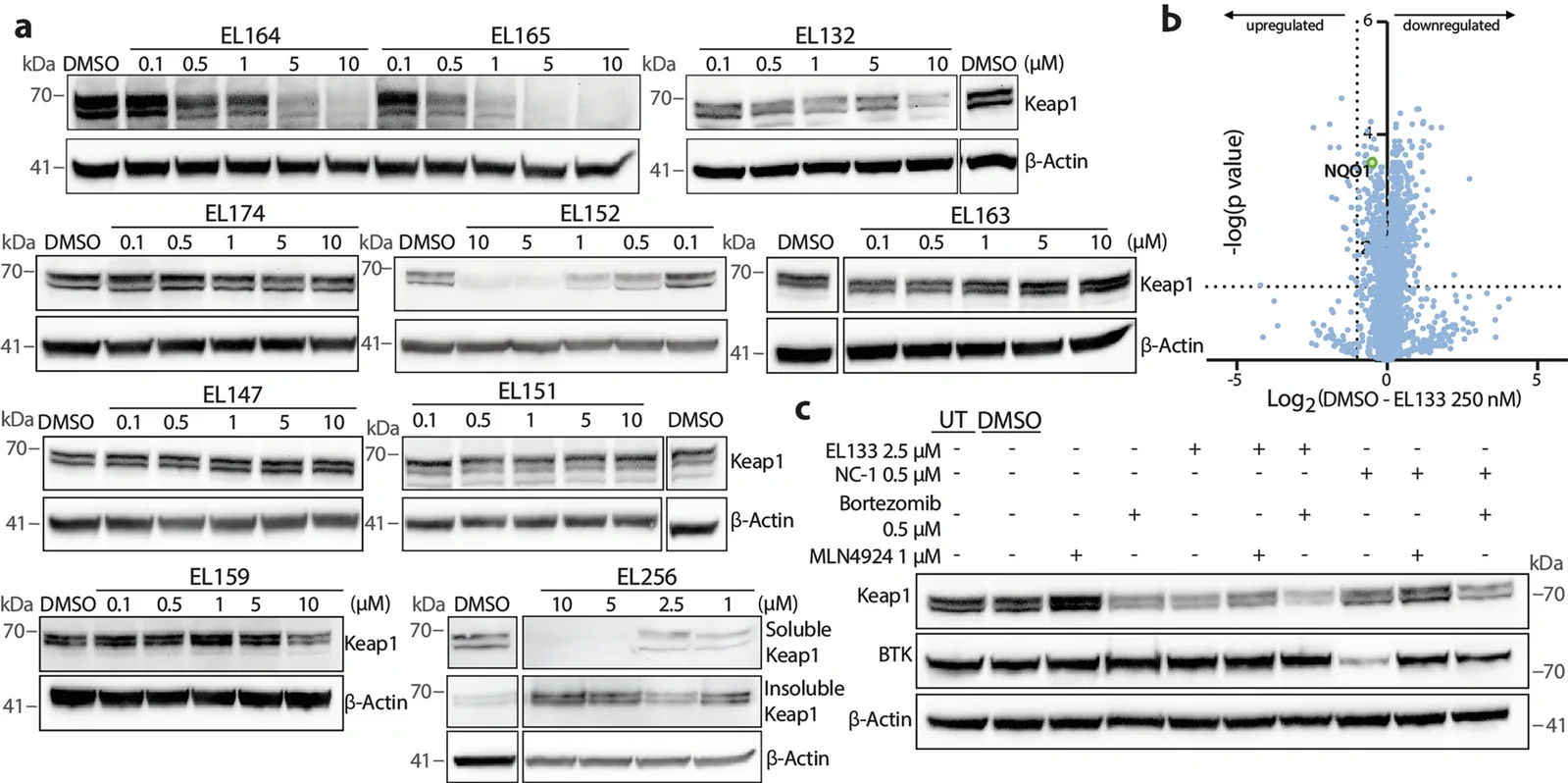
SAR and mechanistic characterization of Keap1 PINCHs. **a**. WB analyses of Keap1-targeting PINCHs in OCI-AML2 cells after 20 h treatments. **b**. Global proteomics analysis of the soluble fraction of U2OS cells treated with 250 nM EL133/DMSO for 20 h. The dotted lines mark a 2-fold upregulation in protein level (vertical) and *p* = 0.05 significance in a double-sided student’s t-test (horizontal). **c**. OCI-AML2 cells pre-treated with Bortezomib or MLN4924 for 1 hour, then with EL133 or NC-1, a BTK-targeting PROTAC^92^, for an additional 7 hours. Source data

As for BCL6, we varied the linker length and composition (Fig. 2a and Extended Data Fig. 2a), which revealed a pronounced structure–activity relationship (SAR). EL152 (**9**), based on intermediary-length PEG6, showed similar activity to EL229 (Extended Data Fig. 2a; half-maximal effective concentration (EC_50_) = 904 nM). EL133 (**8**), based on PEG2, was the most potent compound with EC_50_ = 102 nM (Fig. 2c) and led to the accumulation of Keap1 in the insoluble phase at a concentration as low as 500 nM (Fig. 2d). Moreover, this accumulation occurred in a dose-dependent and time-dependent manner (Fig. 2d). However, similar PEG-based linkers EL147 (**6**) and EL151 (**7**), based on PEG1 and PEG3, respectively, showed no activity, highlighting that the PINCH modality can be highly sensitive to linker length (Extended Data Fig. 2a). Given this apparent sensitivity, we designed several compounds based on the PEG2 design. First, EL164 (**11**) had sulfur atoms replacing the PEG oxygens, and showed good activity in cells, downregulating Keap1 with an EC_50_ value of 434 nM (Extended Data Fig. 2a). Replacing the middle ethylene glycol unit with a piperazine (EL165, **10**) also retained activity in cells (EC_50_ = 342 nM). In another design, EL132 (**12**), we used a six-carbon alkyl linker and downregulated Keap1 with an EC_50_ of 1,024 nM. By contrast, three close analogs of these active compounds, EL163 (**13**) based on an 8-carbon alkyl linker (the same length as EL133), EL159 (**14**) with the same length as EL132 but conformationally restricted and EL174 (**15**), which differs from EL165 by two carbonyl groups, showed no activity (Extended Data Fig. 2a). This subtle SAR mirrors similar trends in bifunctionals such as PROTACs in which small changes in the linker can dramatically impact cellular potency, in part because of cooperative new protein–protein interactions (PPIs) induced by the bifunctional compound^44,54,55^.

Analyzing the crystallographic symmetry of the Keap1–BDX complex (PDB 4CXT; Supplementary Fig. 5a,b), we identified a neighboring Keap1 monomer from an adjacent unit cell positioned such that the BDX-binding sites were close. We hypothesized that EL133 might bridge this crystallographic interface. We used AlphaFold3 (ref. ^56^) to model this complex. With only the sequence of two Keap1 monomers and the EL133 ligand given as input and specifying the covalent attachment points, AlphaFold3 indeed predicted the structure of the crystallographic dimer bridged by EL133 (Supplementary Fig. 5c). This suggests a binding mode through which EL133 could stabilize a low-energy, nonnative interface for Keap1 and, therefore, bind cooperatively.

### PINCH downregulation is selective and proteasome independent

To evaluate the selectivity of EL133, we performed quantitative proteomics. When we lysed cells using RIPA buffer and focused solely on the soluble fraction, Keap1 was significantly downregulated in the treated sample relative to the control (Fig. 2e). Moreover, only a few other proteins were downregulated (Supplementary Data 1), demonstrating selectivity of EL133 for Keap1. To explore the possibility of coprecipitation of the target’s binding partners into the PINCH-induced aggregate, we extracted a list of 63 known PPI partners of Keap1 from UniProt (IntActDatabase^57^; Supplementary Data 1) and analyzed their levels in the proteomics experiment. This revealed only one Keap1-interacting protein (COL1A1) detected as significantly downregulated in the soluble fraction, which suggests that, if interaction partners are ‘pulled’ into the aggregates, it is not a common phenomenon.

Keap1 is also a substrate adaptor protein to a Cullin–RING E3 ligase complex and was shown to induce degradation of targets to which it is recruited^58–61^. Thus, the Keap1 downregulation we observed may result from its self-degradation in a homo-PROTAC manner. Such self-targeting was previously observed for other E3 ligases^62–64^. To test this possibility, we initially treated cells either with MLN-4924 (a NEDDylation inhibitor) or with bortezomib (a proteasome inhibitor), followed by EL133. Inhibiting either of these targets did not rescue Keap1 downregulation by EL133 (Extended Data Fig. 2c), implying that EL133 downregulates Keap1 in a proteasome-independent manner.

**Fig. 3:**
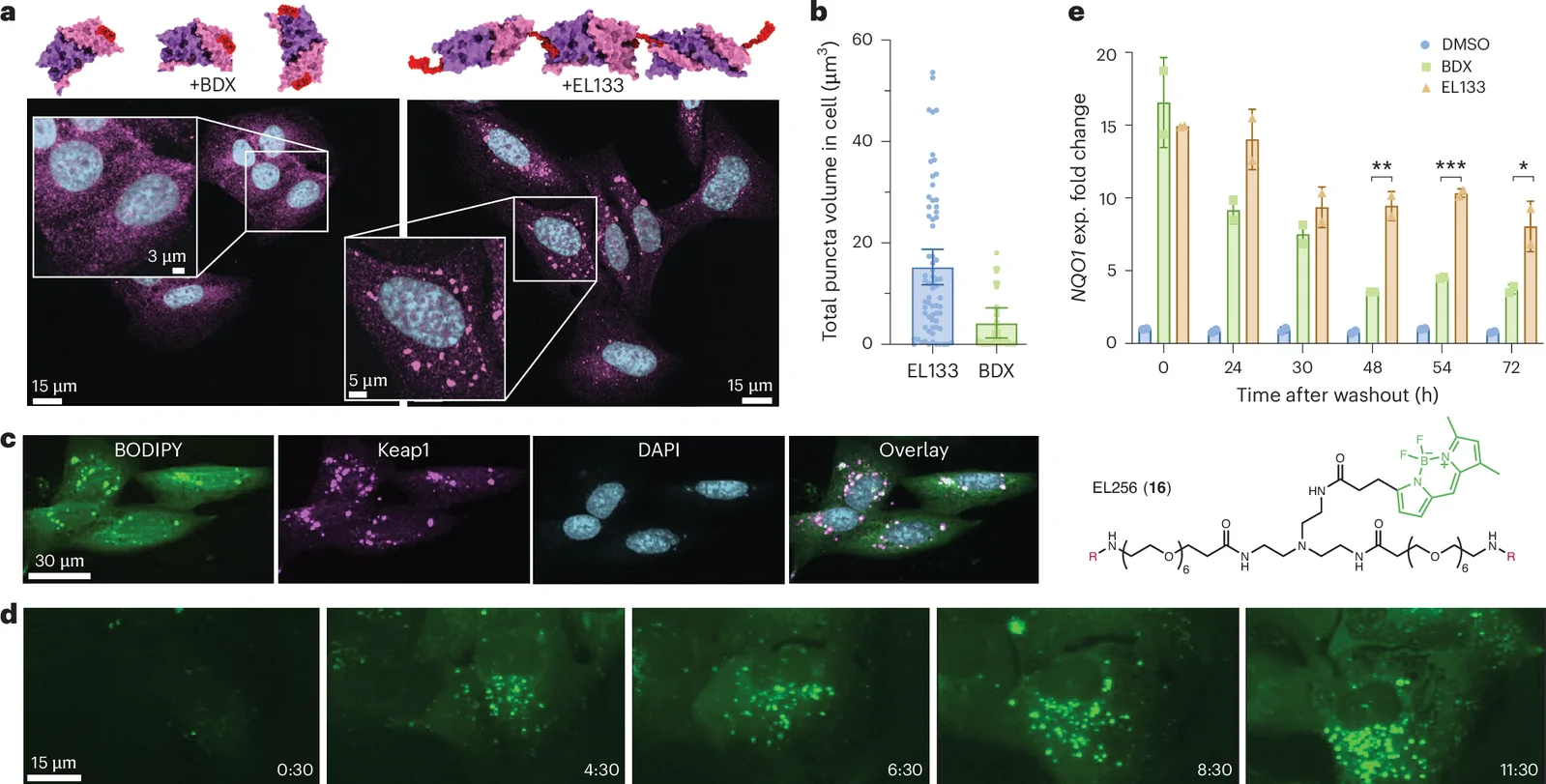
PINCHs drive target supramolecular assembly in cells and exhibit prolonged duration of action. **a**, Immunostaining of U2OS cells following treatment with 250 nM BDX (left) or 250 nM EL133 (right). Keap1 is colored in magenta; DAPI staining is shown in cyan. Additional representative images are shown in Supplementary Fig. 2a. **b**, Quantification of Keap1 puncta in the two treatments shows a significant difference in volume (*P* < 0.0001, two-tailed Mann–Whitney test; *n* = 129 cells analyzed in two independent experiments). Bars show the mean values and a 95% confidence interval. **c**, Immunostaining of U2OS cells following 20 h of treatment with 2.5 μM BODIPY-conjugated PINCH EL256 and its structure. ‘R’ refers to the same structure as in Fig. 2a. Keap1 is colored in magenta; DAPI staining is shown in cyan. Additional representative images and colocalization quantification are provided in Supplementary Fig. 9. **d**, Lattice light-sheet microscope live imaging of U2OS cells, immediately following treatment with 2.5 μM EL256. Time after treatment is indicated in h:min. **e**, qPCR analysis of *NQO1* expression after 20 h of treatment of OCI-AML2 cells with DMSO, EL133 or BDX at 250 nM, followed by washing of the cells. Representative results from two independent experimental replicates with two technical replicates each are shown. Bars show the mean values; error bars show the s.d. *P < 0.05, **P < 0.01 and ***P < 0.001, one-sided unpaired *t*-test. exp., expression. Source data

We visualized the subcellular localization of endogenous Keap1 following PINCH treatment by immunofluorescence in U2OS cells, which are particularly amenable to microscopy. Similarly to BCL6, we observed bright punctate structures in the EL133-treated cells and not in those treated with the parent ligand BDX (*P* < 0.0001; Fig. 3a,b) or with an inactive PINCH EL151 (*P* < 0.0001; Supplementary Fig. 2b,c). To rule out the recently reported^65^ aggregation because of electrophilic stress, we also performed a colocalization experiment with RAS GTPase-activating protein-binding protein 1, showing that EL133 does not induce the formation of its characteristic stress granules (Supplementary Fig. 4).

To follow the cellular localization of the PINCH molecules themselves, we produced a BODIPY-conjugated PINCH EL256 (**16**; Fig. 3c). Its design was based on the long linker of EL229 to minimize interference of the fluorophore with polymerization. A WB analysis of EL256-treated cells confirmed that it retained such ability (Extended Data Fig. 2a). Immunostaining EL256-treated U2OS cells revealed that endogenous Keap1 puncta were colocalized with the BODIPY signal (Fig. 3c and Supplementary Fig. 9), implying incorporation of the PINCH into the Keap1 assemblies and further supporting our proposed mechanism of action for this modality. Taken together, these data indicate that the PINCH downregulates Keap1 by triggering its polymerization into nonsoluble, supramolecular assemblies.

The colocalization also suggests that we may follow the compound’s fluorescence signal as a proxy for the formation of Keap1 polymers in cells. We used live fluorescence imaging of U2OS cells and EL256 to follow the protein’s higher-order assembly formation in real time. This revealed an initial slow uptake of the compound by the cells, followed by puncta formation at 4.5 h after treatment (Fig. 3d). Over the course of several additional hours we observed the formation and expansion of additional puncta (Supplementary Videos 1–3).

### Keap1 PINCHs are longer-acting than monomeric inhibitors

We observed that PINCHs could drive a target protein into forming large macromolecular assemblies, reducing its levels in the soluble fraction and potentially inhibiting its function. This unique mechanism of action prompted us to explore whether it is linked to distinct pharmacodynamic properties, particularly in relation to the duration of action. Keap1 is a negative regulator of Nrf2. We confirmed Nrf2 upregulation by WB, showing a similar effect of BDX and EL133 on its levels (Supplementary Fig. 2e). NAD(P)H quinone dehydrogenase 1 (NQO1) is a well-established downstream target of Nrf2 (refs. ^66,67^) and is, therefore, expected to be upregulated when Keap1 activity decreases. NQO1 was significantly upregulated in the soluble proteomics experiment (Extended Data Fig. 2b). We measured *NQO1* gene expression levels over a 72-h time course by qPCR after subjecting cells to DMSO, BDX or EL133 for 20 h, followed by washout. At the time of washout, both the BDX-treated and the EL133-treated cells showed a similar ~15-fold increase in the expression level of *NQO1*. However, 48 h after treatment, *NQO1*’s expression in the BDX-treated cells returned closer to its original level, whereas it remained higher in the EL133-treated cells (48 h, *P* = 0.0069; 54 h, *P* = 0.0007; 72 h, *P* = 0.0370; Fig. 3e). This result suggests a long-lasting effect of the PINCH and differentiates this modality from traditional inhibition of the target. We performed the same comparison with omaveloxolone (OMVX), a derivative of BDX that recently received US Food and Drug Administration approval for the treatment of Friedrich’s ataxia^68^, in which it had similar effects on *NQO1* gene expression to BDX (Supplementary Fig. 2d). This observation highlights a potential advantage of the PINCH modality relative to other inhibition modes, whereby the duration of action is typically limited by clearance of the compound or half-life of the protein.

### Biophysical characterization of PINCH-induced polymerization

Experiments in cells involve a complex milieu that may affect the PINCH activity. To characterize the mechanism of action of PINCHs independently of potential confounding variables, we evaluated the Keap1–PINCH interaction in vitro with a purified recombinant BTB domain.

**Fig. 4:**
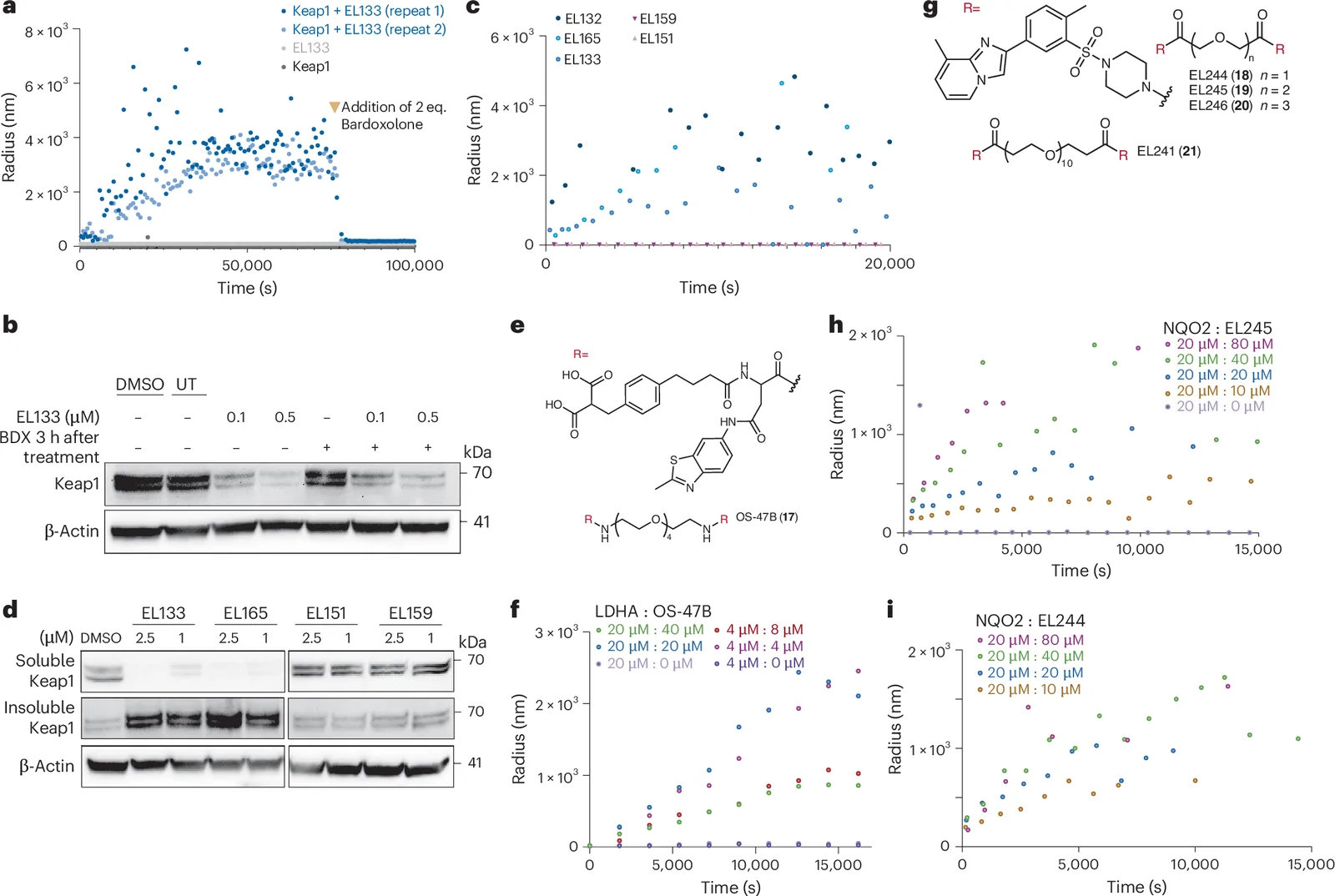
PINCHs induce target polymerization in vitro*.* **a**, DLS experiments following recombinant Keap1 BTB (50 μM; 100 μM binding sites) and EL133 (50 μM; 100 μM binding moieties) over time at room temperature. eq., equivalents. **b**, WB of EL133-treated (20 h) OCI-AML2 cells with or without treatment with excess BDX (5 μM). Representative results from two independent experiments are shown. **c**, Additional Keap1 PINCH WB results in OCI-AML2 cells (20 h) (representative result from two independent experiments). **d**, DLS experiments after treatment with 50 μM recombinant Keap1 BTB with these PINCHs at 50 μM, over time at room temperature. **e**, Chemical structure of LDHA-targeting PINCH OS-47B. **f**, DLS experiments following recombinant LDHA incubated with OS-47B over time at room temperature. The reported concentrations indicate binding sites and binding moieties. Each LDHA tetramer comprises four binding sites and each PINCH comprises two binding moieties. We report the concentrations of monomers (for example, 5 μM LDHA and 20 μM PINCH are indicated as 20 μM:40 μM binding sites:binding moieties). **g**, Chemical structures of NQO2-targeting PINCHs. **h**,**i**, DLS experiments following recombinant NQO2 incubated with EL245 (**h**) or EL244 (**i**) over time at room temperature. NQO2 is dimeric (two binding sites) and the concentrations are reported in the same way for LDHA (binding sites:binding moieties). Source data

We used dynamic light scattering (DLS) to follow the polymerization of Keap1 BTB domain in the presence of EL133. After about 4 h, we observed large molecular species forming that did not appear in samples containing only Keap1, EL133 or BDX (Fig. 4a and Supplementary Fig. 7b). As BDX is a reversible covalent binder of Keap1, we hypothesized that EL133-induced polymer formation should be reversible as well, such that the addition of monomeric BDX could dissolve the large assemblies. Indeed, adding two equivalents of BDX led to the disassembly of Keap1 polymers (Fig. 4a). Surprisingly, this phenotype did not reproduce in the cellular environment; when treating cells with EL133 for 20 h and then adding a large excess of BDX, no change in Keap1 levels in the soluble phase occurred, as seen by WB (Fig. 4b).

We tested additional BDX-based PINCHs with recombinant Keap1 in the same manner and observed the formation of large species over time with EL165 and EL132 but not with EL151 and EL159 (Fig. 4c), mirroring these compounds’ activities in cells (Fig. 4d and Supplementary Fig. 7c). In addition, preincubating Keap1 with BDX before adding EL133 prevented the formation of large Keap1 structures, showing that these are dependent on the BDX-binding site (Supplementary Fig. 7a).

**Extended Data Fig. 3:**
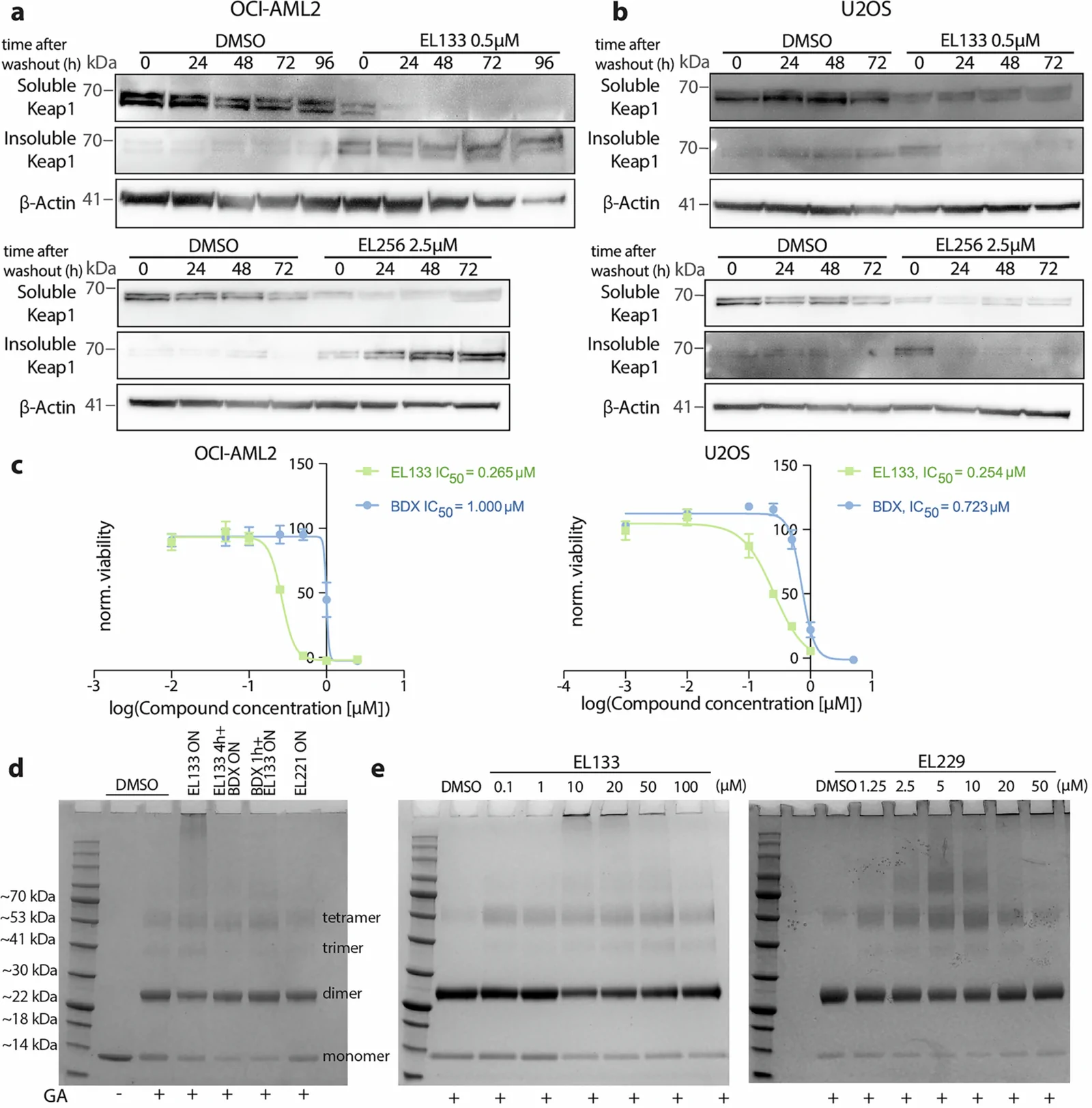
Duration of PINCH induced precipitation and effects on cell viability. **a**. WB of OCI-AML2 cells treated with 500 nM EL133 (top) or 2.5 µM EL256 (bottom) for 20 h followed by washing of the cells, and readout at different times after washout. **b**. WB of U2OS cells treated with 500 nM EL133 (top) or 2.5 µM EL256 (bottom) for 20 h followed by washing of the cells, and readout at different times after washout. **c**. Viability of OCI-AML2 and U2OS cells 72 h after treatment with BDX or EL133, (technical replicates n = 6 for OCI-AML2, n = 4 for U2OS). Data depicting mean and standard deviation values. **d**. *In vitro* polymerization of Keap1-BTB followed by GA treatment, separated on an SDS-PAGE gel and stained by Coomassie blue. Samples contain 50 µM Keap1. The concentrations used are: 50 µM for EL133; 50 µM for EL221; and 100 µM for BDX and compounds were added as indicated. **e**. *In vitro* polymerization of 10 µM Keap1-BTB with compounds at indicated concentrations, incubated overnight at room temperature, followed by GA treatment, shown on an SDS-PAGE gel stained by Coomassie blue. **d** + **e** show representative results from n = 2 independent experiments. Source data

As a complementary approach to follow in vitro polymerization of recombinant Keap1, we used glutaraldehyde (GA) as a crosslinking agent to capture the higher-order state of EL133-induced Keap1. BDX’s reversible covalent bond is lost during sample preparation for SDS–PAGE gel; however, GA forms multiple covalent ‘bridges’ between protein units along the polymer, allowing us to follow the formation of multimeric states of Keap1, induced by EL133. As expected, Keap1 samples treated with active PINCH EL133 induced the formation of higher-order assemblies (Extended Data Fig. 3d). Reversal of polymer formation through the addition of excess BDX and rescue of polymerization through preincubation with BDX were also reproduced in this setting. This approach allowed us to further clarify a potential ‘hook’ effect^69,70^ of PINCHs, which was not apparent in a cellular setting, potentially because of solubility issues. Both EL133 and EL229 (Extended Data Fig. 3e) exhibit peak polymerization activity around a 1:1 ratio of protein-binding sites to compound-binding moieties, with little to no effect when the compound concentration was tenfold lower or higher than that of the protein.

As BDX and our Keap1 PINCHs covalently engage with C151 in Keap1 (ref. ^53^), we produced a Keap1 BTB construct with a C151S substitution to follow the dependence of EL133 on covalent binding. DLS experiments revealed that EL133 is able to form large species in solution with the wild-type (WT) protein but not with the mutant (Supplementary Fig. 6a). Similarly when using GA crosslinking, we observed by gel a ‘smear’ of high-molecular-weight species when EL133 was incubated with WT Keap1 but not with the C151S mutant (Supplementary Fig. 6b).

To gain additional insight into the supramolecular structure of Keap1 in the presence of EL133, we applied negative-stain electron microscopy. This revealed filaments after incubation with EL133 but not after BDX treatment (Supplementary Fig. 8a). In addition, letting Keap1–EL133 polymers form and then adding an excess of BDX to the sample resulted in the disappearance of these filaments (Supplementary Fig. 8b), recapitulating the DLS results.

As both Keap1 and BCL6 dimerize through a BTB domain, we also synthesized and similarly evaluated PINCHs for two additional homomeric proteins. Lactate dehydrogenase A (LDHA) forms a homotetrameric structure. We functionalized its inhibitor developed by Ward et al.^71^ to synthesize an LDHA-targeting PINCH OS-47B (**17**; Fig. 4e). DLS measurements of recombinant LDHA incubated with the PINCH revealed the formation of larger species over time (Fig. 4f). A molar ratio of 1:1 in binding moieties to binding sites resulted in the most effective polymerization in vitro, consistent with expectations. Nonetheless, polymerization was also observed with a twofold excess in favor of either the binding site or the binding moiety. It also persisted with larger stoichiometric differences with an excess of binding moieties (up to eightfold), progressively leading to smaller assemblies, presumably because of saturation of LDHA-binding sites (Supplementary Fig. 7d). Conversely, subconcentrations of PINCH (down to 1:8 binding moieties to binding sites) also led to smaller assemblies, as expected from a lower concentration of ‘crosslinks’. Immunostaining U2OS cells following OS-47B treatment revealed no effect on LDHA localization (Supplementary Fig. 7i).

NQO2 is a homodimeric protein with a role in cellular protection against oxidative stress and has been implicated in neurodegenerative diseases, making it an age-related metabolic risk factor^72,73^. We used YB-537, a reported inhibitor of NQO2 (ref. ^74^), to design a set of PINCHs based on PEG linkers of different lengths (Fig. 4g). Contrary to the case of BCL6, where only longer linker lengths allowed polymer formation, here, PEG10-bearing EL241 (**21**) showed little to no effect in polymerizing NQO2 (Supplementary Fig. 7f). On the other hand, compounds EL244 (**18**), EL245 (**19**) and EL246 (**20**), comprising PEG1, PEG2 and PEG3 linkers, respectively, all showed the ability to form larger species in solution when incubated with NQO2 in a dose-dependent manner (Fig. 4h,i and Supplementary Fig. 7f). In the case of EL244 and EL245, we observed that even a submolar ratio of PINCH was sufficient to induce polymerization. Additionally, even though we would expect binding-site saturation at high PINCH concentration, we observed polymer formation with up to fourfold excess compound. To try and understand the polymerization dependence on the linker length, we followed all NQO2 PINCHs and the parent amine inhibitor using an enzymatic activity assay (Supplementary Fig. 7h). All compounds showed similar IC_50_ values, suggesting that they are able to monomerically bind NQO2 (as an inhibitor); however, polymerization apparently has additional requirements that were fulfilled only by the short-linker compounds in this set. Using WB to test NQO2 PINCHs active in vitro in U2OS cells at several concentrations revealed no effect on soluble protein levels (Supplementary Fig. 7g), perhaps because of permeability issues.

### PINCH-induced assembly effects are cell type dependent

A question that remains open is what happens to the PINCH-induced polymers that form and precipitate in the cells and how does the cell clear these formations, if at all. To explore this, we treated OCI-AML2 and U2OS cells with EL133 or EL256 for 20 h to induce Keap1 polymerization, followed by washing the cells and probing them over several time points (Extended Data Fig. 3a,b). Interestingly, these two cell lines exhibited profoundly different behavior in the presence of insoluble Keap1. Following OCI-AML2 cell treatment with 500 nM EL133 or 2.5 µM EL256, Keap1 was absent from the cell’s soluble phase and remained accumulated in the insoluble phase for up to the 96 h that we tested (Extended Data Fig. 3a). However, in U2OS cells, Keap1 insoluble accumulation appeared at the initial time point (after 20 h of compound treatment) but did not appear in the following ones (Extended Data Fig. 3b). In both cell lines, soluble Keap1 remained absent from the cell throughout the experiment.

To visualize the fate of the Keap1–PINCH polymers, we treated U2OS cells with EL256 for 20 h, then washed the cells and performed immunostaining imaging after 24, 48 and 72 h. We observed puncta formation when imaging cells after treating them for 20 h (Fig. 3c,d); however, after washing the EL256 away, the cells lost most of the puncta within 24 h, corroborating the WB data. Furthermore, 48 and 72 h after wash, minimal BODIPY and Keap1 puncta signals remained and the endogenous Keap1 signal was diffused in the cell (Supplementary Fig. 10).

An additional difference between cell types in PINCH tolerance was reflected in the viability experiments. Comparing IC_50_ values of EL133 and BDX through viability measurements, we expected a twofold difference in their respective cellular effects, considering that every PINCH contains two BDX copies. We observed that OCI-AML2 cells were more sensitive to EL133 compared to U2OS (Extended Data Fig. 3c), with fourfold and threefold differences over BDX, respectively, possibly reflecting their difference in ability to clear the polymers. On the contrary, in the case of Mino, Ramos and HEK 293 cells, no additional viability loss was observed when comparing BDX and EL133 (Supplementary Fig. 2f). As recently observed, large polymers of folded proteins do not appear generally toxic; thus, we anticipate that sensitivity to the molecule is dependent on the functional effects of the PINCH rather than presence of the polymer^75–77^.

### A hetero-PINCH simultaneously downregulates Keap1 and BCL6

**Fig. 5:**
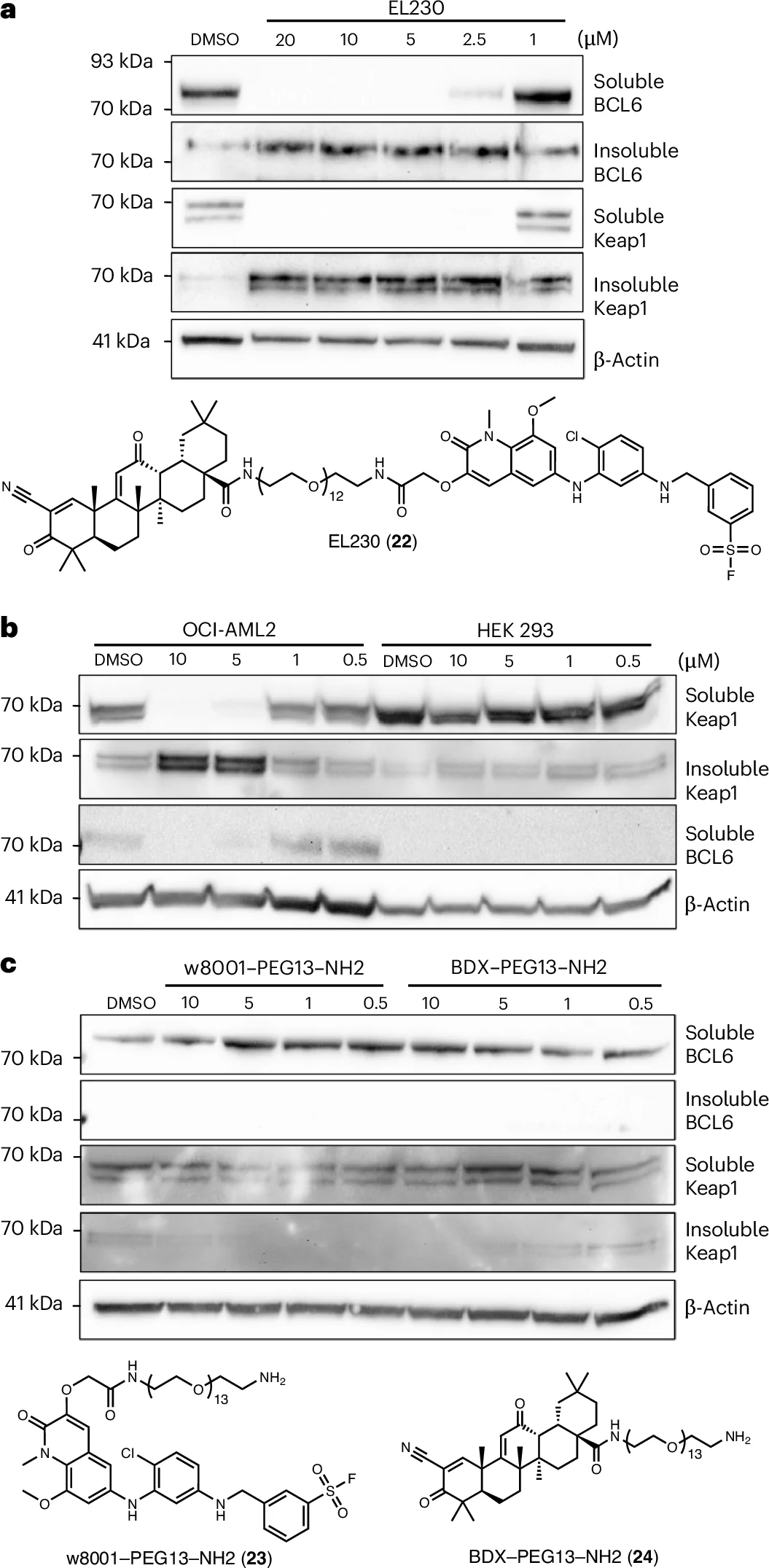
Hetero-PINCHs can simultaneously coprecipitate two targets. **a**, WB and structure of EL230 after 20 h of treatment in Mino cells. **b**, EL230 tested in a BCL6-positive cell line (OCI-AML2) and in a BCL6-negative cell line (HEK 293) after 20 h of treatment, showing inactivity in the latter. **c**, Monomeric versions of EL230, based on BCL6 and Keap1 ligands with linkers, tested after 20 h of treatment in Mino cells. All WBs shown here are representative results from *n* = 2 independent experiments. Source data

In certain scenarios, the simultaneous elimination of more than one target is of therapeutic or biological interest. Accordingly, we propose that two homomeric targets could be induced to coprecipitate by a single PINCH. To illustrate this concept, we synthesized a PINCH consisting of BDX and w8001 on either end (Fig. 5a) to bind Keap1 and BCL6 dimers, respectively. Theoretically, this molecule should induce the formation of a polymer consisting of Keap1 and BCL6 dimers in an alternating fashion.

We tested the effect of hetero-PINCH EL230 (**22**) in Mino cells that express high levels of BCL6 and we observed a reduction in both Keap1 and BCL6 levels in the soluble phase, as well as their corresponding accumulation in the insoluble phase, in a dose-dependent manner (Fig. 5a). To test the dependence of hetero-PINCH activity on the presence of both target proteins, we tested EL230 in two additional cell lines, OCI-AML2 and HEK 293, as the latter does not express BCL6. In OCI-AML2 cells, Keap1 and BCL6 were downregulated simultaneously, whereas Keap1 remained unchanged in HEK 293 cells (Fig. 5b). In addition, monomeric versions of the compounds, consisting of one ligand attached to a linker (compounds **23** and **24**) did not induce such effects on either target in Mino cells (Fig. 5c).

## Discussion

We introduce a novel pharmacological modality for inactivating protein targets through a mechanism that relies on their induced sequestration by polymerization. We applied this approach against two homodimeric proteins in cells and against two additional homomeric proteins in vitro.

This modality is reminiscent of PROTACs, which also involve bifunctional ligands and induce the degradation of a protein target. However, PROTACs require binding to a specific E3 ligase, thereby necessitating its coexpression and colocalization with the target protein of interest. By contrast, PINCHs do not require such additional machinery beyond the target itself. Additionally, while PROTACs require a precise interaction geometry to promote ubiquitination, the PINCH modality, in principle, does not require a highly specific interaction geometry to promote high-order assembly and sequestration. This relaxed requirement can be exploited to scout initial activity using long linkers (Figs. 1b and 2b), whereas optimization of the linker length and chemical nature for maximal potency can be performed later (Fig. 2c and Extended Data Fig. 2a).

While PINCHs are associated with relaxed constraints in terms of the subunit recognition geometry, they do require the bifunctional ligand to (1) act as a bridge between two oligomers and (2) minimize the formation of finite oligomers such as a dimer of dimers (Supplementary Fig. 11) or more generally closed rings. Taking into account the homo-oligomer structure can help in target selection and ligand design for both considerations. Additionally, we anticipate that homo-oligomers with dihedral symmetry will be less susceptible to form closed geometries^20,26,78,79^. As an example, a recent dimeric binder of Keap1 was reported to inhibit it in cells, without sequestration from the soluble fraction^80^. On top of these geometric constraints, concentration is an important property to consider, whereby a ligand concentration that is too low or too high would inhibit high-order assembly through the hook effect^69,70^. Additionally, although we show examples for noncovalent (LDHA and NQO2), reversible covalent (Keap1) and irreversible covalent (BCL6) PINCHs, the role of covalency in promoting efficient polymerization and its interplay with ligand–target stoichiometry and kinetics remain to be fully characterized, as achieved for other covalent proximity inducing modalities^81–83^.

Phenotypically, the PINCHs tested in cells exhibited different functional outcomes compared to their monomeric parent inhibitors. BCL6 PINCHs showed complete elimination of c-Myc, which was not seen with the parent monomeric inhibitor or degrader (Fig. 1c). Similarly, at 72 h, only the active PINCHs induced a viability effect (Fig. 1g and Supplementary Fig. 1e) without an apparent effect on cells not expressing the target and in contrast to reported BCL6 degrader BI-3802, which showed no viability effect on either cell type on this time scale. Additionally, Keap1 PINCHs showed a significantly longer duration of action when compared to the monomeric BDX and second-generation inhibitor OMVX (Fig. 3e and Supplementary Fig. 2d), perhaps because of the persistence of the aggregate following compound washout. While the precise mechanisms underlying these differences warrant further investigation, these results clearly demonstrate that PINCHs can complement other pharmacological modalities.

The scope of this technology might seem limited by the requirement for symmetrical oligomeric targets. However, our recent analysis^84^ suggested that 20% of the human proteome fulfills this requirement as homo-oligomers. Moreover, an additional 30% of the proteome participates in symmetric hetero-oligomers that can be targeted by PINCHs in principle. The requirement for symmetry also adds a dimension of selectivity for PINCHs. While the monomeric ligand recruiter might not be very selective (for example, BDX), only a fraction of its off-targets would also fulfill the symmetry and geometry requirements for polymerization. This may be reflected in the relatively few off-targets downregulated by EL133 (Fig. 2e). We should also note that the technology can in theory also be applied to nonsymmetric systems. One could achieve polymerization of heteromeric complexes with hetero-PINCHs harboring different ligands on either end, targeting the two binding partners. In principle, a monomeric protein could also be targeted through two different binding sites, for example, using imatinib^85^ and asciminib^86^ to polymerize ABL kinase.

To conclude, we present here a novel modality for eliminating a target protein from the cellular soluble phase by inducing its polymerization. This approach can target a large fraction of the human proteome and complement existing modalities. Its simple design principles should facilitate its adoption and application against a variety of new targets, expanding our pharmacological toolbox along with the space of targetable proteins.

## Methods

### WB

Cells were incubated with DMSO at 0.1% or with compound in concentrations ranging 10–20,000 nM for 20 h unless indicated differently. Following incubation, lysis was performed in RIPA buffer and the samples were measured for total protein quantification by BCA assay (Thermo Fisher Scientific, 23225). Then, 20–40 μg of each sample was loaded and run on an SDS–PAGE gel, before transferring onto a nitrocellulose membrane using a Trans-Blot Turbo system (Bio-Rad) and blocking using a 5% BSA in TBS-T (w/v) solution for 1 h at room temperature. Incubation with primary antibodies (mouse anti-BCL-6 (sc-7388, Santa Cruz, 1:500; overnight at 4 °C), mouse anti-Keap1 (sc-365626, Santa Cruz, 1:500; overnight at 4 °C), rabbit anti-LDHA (4H18L6, Thermo Fisher, 1:1,000; overnight at 4 °C), mouse anti-NQO2 (sc-271665, Santa Cruz, 1:500; overnight at 4 °C), rabbit anti-c-Myc (5605, Cell Signaling, 1:1,000; overnight at 4 °C) and mouse anti-β-actin (3700, Cell Signaling, 1:1,000; 1 h at room temperature) was performed and then the membranes were washed and incubated with a horseradish-peroxidase-conjugated secondary antibody (mouse 7076/rabbit 7074, Cell Signaling) at room temperature for 1 h. Imaging of the chemiluminescence signal was performed using a ChemiDoc XRS+ instrument (Bio-Rad). Primary band quantifications were normalized against β-actin bands.

### Overexpression of BCL6(1–275)–GFP

HEK 293 cells were cultured in antibiotic-free medium. At cell confluency of ~70%, transfection was performed using 0.5 μg of plasmid (produced from a bacteria agar stab obtained from Addgene, 169932) per well, to be incubated overnight. The medium was then replaced and compound treatments were introduced as described in the respective experiments.

### Label-free quantitative proteomics

Cells were incubated with DMSO at 0.1% or with compound for 20 h unless indicated differently, in quadruplicate. Following incubation, the cells were lysed in either a 5% SDS and 50 mM ammonium bicarbonate solution or in a mixed detergent buffer (cells were lysed using detergent based buffer containing 50 mM HEPES pH 8.0, 1% SDS, 1% Triton X-100, 1% NP-40, 1% Tween-20, 1% sodium deoxycholate, 50 mM NaCl, 5 mM EDTA and 1% (w/v) glycerol) and probe-sonicated for the shearing of DNA. Samples were then reduced using DTT, followed by reaction with iodoacetamide. The labeled proteins were then precipitated on a dispersion of glass beads, followed by overnight trypsin digestion and purification of peptides. Samples were analyzed using an EASY-nLC 1200 nano-flow ultrahigh-performance LC system, equipped with a PepMap RSLC C18 column (particle size: 2 µm, pore size: 100 Å, diameter: 75 µm, length: 50 cm), mounted using an EASY-Spray source onto an Exploris 240 MS instrument. LC–MS-grade solvents were used for all chromatographic steps at 300 nl min^−1^. The mobile phase consisted of solvent A (H_2_O + 0.1% formic acid) and solvent B (80% acetonitrile + 0.1% formic acid). Peptides were eluted from the column into the MS instrument using the following gradient: 1–40% B in 160 min, 40–100% B in 5 min, maintained at 100% for 20 min, 100–1% in 10 min and finally 1% for 5 min. Ionization was achieved using a 2,100-V spray voltage with an ion transfer tube temperature of 275 °C. Initially, data were acquired in data-independent acquisition mode. MS1 resolution was set to 60,000 (at 200 *m*/*z*), a mass range of 370–1,450 *m*/*z*, normalized automatic gain control of 300% and maximum injection of 20 ms. MS2 resolution was set to 30,000, with 31 isolation windows of 19 Da with a 1-Da overlap, maximum injection time of 50 ms and higher-energy collision-induced dissociation collision energy at 27%. Four samples were analyzed per condition.

Data analysis was performed using Fragpipe^87,88^ (version 22.0) using the MSFragger search engine (version 4.1), and DIANN 1.9. Analysis was performed using a human proteome database from December 2022 (UniProt) with contaminants added, including streptavidin added manually as a contaminant. MSFragger analysis was performed using trypsin as the enzyme that cuts after arginine and lysine, with up to two missed cleavages, peptide length of 7–50 and the N-terminal methionine removed. N-terminal acetylation and methionine oxidation were defined as variable modifications and carbamidomethyl was defined as a fixed modification. A false discovery rate of 0.01 was used at both the peptide level and the protein level. Label-free quantification was performed using DIANN. After analysis, the quantified protein file was analyzed using Perseus^89^. Intensities were converted to log_2_ values, the quadruplicates of each type were grouped and missing values were replaced by 13. Differences and *P* values were calculated using a double-sided Student’s *t*-test. *P* values were adjusted using the Benjamini–Hochberg method^90^. The MS proteomics data were deposited to the ProteomeXchange Consortium through the PRIDE^91^ partner repository with the dataset identifier PXD067656 for Supplementary Data 1.

### Source of cell lines

OCI-AML2 cells were a gift by M. Minden, Princess Margaret Cancer Center, University Health Network. All other cell lines were obtained from commercial resources: Mino, American Type Culture Collection (ATCC; CRL-3000); Ramos, ATCC (CRL-1596); HEK 293, ATCC (CRL-1573); U2OS, ATCC (HTB-96).

### Purification of Keap1-S172A (48–180)

The Keap1 BTB domain (48–180) encoding the single substitution S172A was cloned into pET28-bdSumo vector generating His–bdSumo–Keap1-S172A. A 7.5-L culture of BL21(DE3) was induced with 200 µM IPTG and grown at 18 °C overnight. The cells were harvested and lysed by a cooled cell disrupter (Constant Systems) in lysis buffer (50 mM Tris pH 8, 150 mM NaCl, 1 mM DTT, 10 mM imidazole and 10% glycerol) containing 0.2 mg ml^−1^ lysozyme, 20 µg ml^−1^ DNase, 1 mM MgCl_2_, 1 mM PMSF and protease inhibitor cocktail. After clarification by centrifugation (38,000*g* for 30 min), the lysate was incubated with 5 ml of Ni beads (Adar Biotech, prewashed with lysis buffer) for 1 h at 4 °C. After removing the supernatant by centrifugation, the beads were washed three times with 50 ml of lysis buffer and once with lysis buffer containing 50 mM imidazole (1 mM TCEP replacing the DTT). Keap1 eluted from the beads following incubation of the beads with 20 ml of cleavage buffer (50 mM Tris pH 8, 0.15 M NaCl, 1 mM DTT, 2 mM MgCl_2_, 10% glycerol and bdSumo protease) for 2 h at room temperature. The supernatant containing the cleaved Keap1 was concentrated and applied to a size-exclusion column (HiLoad_16/60_Superdex200, GE Healthcare) equilibrated with 150 mM NaCl, 25 mM Tris-HCl pH 8.0 and 1 mM TCEP. Fractions containing pure Keap1 were pooled, concentrated and frozen (−80 °C) in aliquots.

### Purification of Keap1-C151S;S172A (48–180)

The Keap1 BTB domain (48–180) encoding the double substitution C151S;S172A was cloned into a pET28-Sumo vector generating His–bdSumo–Keap1-C151S;S172A. A 4-L culture of BL21(DE3) was induced with 400 µM IPTG and grown at 16 °C overnight. The cells were harvested and lysed by sonication in lysis buffer (50 mM HEPES pH 7.5, 500 mM NaCl and 25 mM imidazole) containing 0.1 mg ml^−1^ lysozyme and protease inhibitor cocktail. After clarification by centrifugation, the lysate was filtered and loaded on a 5-ml HisTrap HP column (Cytiva). The column was washed with 25 ml of lysis buffer and 25 ml of lysis buffer + 10% lysis buffer that had a higher concentration of imidazole (300 mM). The protein was eluted using lysis buffer + 300 mM imidazole and digested with Ulp1 (1:100 molar ratio) overnight at 4 °C while dialyzing against the lysis buffer. The dialyzed sample was passed through the HisTrap HP column again to remove cleaved His–SUMO and His–Ulp1, concentrated and injected into a Hiload Superdex 75 column equilibrated with 25 mM HEPES pH 7.5 and 0.5 M NaCl. Fractions containing pure Keap1-C151S;S172SA were pooled and frozen (−80 °C) in aliquots.

### Purification of BCL6 BTB domain (5–129)

The BCL6 BTB domain (5–129) was cloned into the pET28-bdSumo vector generating His–bdSumo–BCL6 BTB. The cell culture, lysis and capture on Ni beads were all performed as described for Keap1 BTB with the exception that PBS was used as the buffer for lysis and Ni capture. Cleaved BCL6 eluted from the beads following incubation of the beads with 20 ml of cleavage buffer (PBS supplemented with bdSumo protease) for 2 h at room temperature. The supernatant containing the cleaved BCL6 was pooled and diluted threefold with 50 mM Tris pH 8 and loaded onto a Tricorn Q 10/100 GL anion-exchange column. Pure BCL6 eluted at 250 mM NaCl when a linear gradient to 1 M NaCl was applied. The pooled pure protein was supplemented with 5 mM DTT and flash-frozen (−80 °C) in aliquots.

### Purification of LDHA

The gene encoding full-length WT human LDHA was cloned into pET28 with an N-terminal His_6_ tag followed by a tobacco etch virus protease cleavage site and was expressed in *Escherichia* *coli* BL21(DE3) cells. Overnight precultures were grown at 37 °C with shaking and used to inoculate 5 L of LB medium. Cultures were grown at 37 °C to an optical density at 600 nm (OD_600_) of 0.6, followed by induction with 200 µM IPTG and further incubation for 4 h at 37 °C with shaking. Cells were harvested by centrifugation and resuspended in lysis buffer (50 mM Tris pH 8, 500 mM KCl and 20 mM imidazole) supplemented with 0.2 mg ml^−1^ lysozyme, 20 µg ml^−1^ DNase I, 1 mM MgCl_2_ and protease inhibitor cocktail (Calbiochem set 3). Lysis was performed using a cooled Constant Systems cell disrupter and the soluble fraction was clarified by centrifugation. Purification was carried out by immobilized metal affinity chromatography (IMAC) using a HisTrap HP 5-ml column (Cytiva) on an ÄKTA fast protein LC (FPLC) system (Cytiva). After extensive washing with lysis buffer, LDHA was eluted with lysis buffer containing 500 mM imidazole. Pooled fractions containing LDHA were concentrated and subjected to size-exclusion chromatography (SEC) on a HiLoad 16/60 Superdex 75 column equilibrated with 50 mM Tris pH 7.5 and 150 mM NaCl. The peak fractions were analyzed by SDS–PAGE, aliquoted, flash-frozen in liquid nitrogen and stored at −80 °C.

### Purification of NQO2

The gene encoding NQO2 was cloned into pET28 with an N-terminal MGSSHHHHHHSAG segment and was expressed in *E*. *coli* BL21(DE3) cells. Overnight precultures were grown at 37 °C with shaking and used to inoculate 5 L of LB medium. Cultures were grown at 37 °C to an OD_600_ of 0.6, followed by induction with 200 µM IPTG and further incubation overnight at 15 °C with shaking. Cells were harvested by centrifugation and resuspended in lysis buffer (50 mM Tris pH 8, 300 mM NaCl and 10% glycerol) supplemented with 0.2 mg ml^−1^ lysozyme, 20 µg ml^−1^ DNase I, 1 mM MgCl_2_ and protease inhibitor cocktail (Calbiochem set 3). Lysis was performed using a cooled Constant Systems cell disrupter and the soluble fraction was clarified by centrifugation. Purification was carried out by IMAC using a HisTrap HP 5-ml column (Cytiva) on an ÄKTA FPLC system (Cytiva). After extensive washing with lysis buffer containing 20 mM imidazole, NQO2 was eluted with lysis buffer containing 500 mM imidazole. Pooled fractions containing NQO2 were concentrated and subjected to SEC on a HiLoad 16/60 Superdex 200 column equilibrated with 50 mM Tris pH 8, 50 mM NaCl and 10% glycerol. The peak fractions were analyzed by SDS–PAGE, aliquoted, flash-frozen in liquid nitrogen and stored at −80 °C.

### DLS

DLS samples were prepared in the respective protein’s elution buffer, to which 0.01% Tween was added, along with the compound from a DMSO stock to obtain a final DMSO concentration of 2%. Samples were prepared at room temperature and measured at a constant temperature of 25 °C. All samples were passed through 0.1-μm filters and then placed in a clear-bottom black 384-well plate. DLS data were recorded using a DynaproPlate Reader III (Wyatt Technology). Data were processed with the supplied DYNAMICS software.

### qPCR for *NQO1* expression

Cells were treated with either DMSO or compound at 250 nM, followed by washing out the treated medium after 20 h and reseeding in fresh medium. Cells were then collected at several time points post washout. Total RNA was extracted using RNeasy mini kit (Qiagen, 74104). RNA concentration and purity were determined by Nanodrop and 500–1,000 ng of RNA per sample was used for complementary DNA (cDNA) synthesis using a high-capacity cDNA reverse transcription kit (Applied Biosystems, 4368814). qPCR (10-µl reaction in a 96-well plate format) was performed with Fast-SYBR green master mix (Applied Biosystems, 4385612), using 1–5 ng of cDNA and the following primers (ordered from Integrated DNA Technologies): *NQO1* forward, CCGTGGATCCCTTGCAGAGA; *NQO1* reverse, AGGACCCTTCCGGAGTAAGA; *GAPDH* forward, ACCCACTCCTCCACCTTTGA; *GAPDH* reverse, CTGTTGCTGTAGCCAAATTCGT. PCR amplification was carried out in Step-One Plus thermocycler (Applied Biosystems). Relative quantification was performed using standard curves. *GAPDH* was used for normalization.

### qPCR for c-Myc expression

Cells were treated for 20 h with the indicated concentrations. RNA concentration and purity were determined by Nanodrop and 1,000 ng of RNA per sample was used for cDNA synthesis using high-capacity cDNA reverse transcription kit (Applied Biosystems, 4368814). qPCR (10-μl reaction in a 96-well plate format) was performed with Fast-SYBR green master mix (Applied Biosystems, 4385612), using 1 ng of cDNA and the following primers (ordered from Integrated DNA Technologies): *MYC* forward, GGCTCCTGGCAAAAGGTCA; *MYC* reverse, CTGCGTAGTTGTGCTGATGT; *GAPDH* forward, ACCCACTCCTCCACCTTTGA; *GAPDH* reverse, CTGTTGCTGTAGCCAAATTCGT. PCR amplification was carried out in Quant-Studio 5 (Applied Biosystems). Relative quantification was performed using standard curves. *GAPDH* was used for normalization.

### Viability measurements

Cells were seeded in a transparent-bottom, black 96-well plate, at 15,000 cells per well in sixfold replications. in 90 µl of medium and left to adhere for at least 4 h. The cells were then treated with the respective compound by adding 10 µl of medium–-compound mixture for a final volume of 100 µl in each well. Cell-Titer blue reagent was used to measure viability according to the manufacturer’s instructions. Plate reader measurements were performed on a Tecan Spark Control 10 M fluorescence system.

### Immunostaining

For spinning disk confocal microscopy, cells were allowed to adhere to a 14-mm glass-bottom plate (MatTek P35G-1.5-14-C), followed by fixation with 3.7% formaldehyde for 5 min and permeabilization with 0.1% Triton X-100 for 10 min. The cells were then blocked with 5% BSA for 60 min at room temperature. Cells were stained with primary antibody, at a concentration of 1:100, for 3 h at room temperature and were then subjected to a secondary antibody conjugated to Alexa Fluor, at a concentration of 1:100–500, for 1 h at room temperature. Lastly, cells were also stained with DAPI for 10 min at room temperature.

### Immunostaining imaging

Imaging of the cells was performed using a Dragonfly spinning disk confocal system (Andor Technology) connected to an inverted Leica Dmi8 microscope (Leica Microsystems). The signals were detected using a scientific complementary metal–oxide–semiconductor Zyla (Andor) 2,048 × 2,048 camera and the bit depth was 16. At the specified intervals, *Z* stacks (0.17 µm) of selected cells were acquired with an HC PL APO ×63/1.3 GLYC CORR CS2 objective (506353, Leica Microsystems).

### Image analysis

Total aggregate volume was calculated per cell using Imaris software (version 10.2). Automatic aggregate segmentation was conducted using the surface module with manual threshold fixed for all images of the same experiment (default 2-pixel smoothing, background subtraction with 0.9-μm largest sphere, morphological split with 0.5-μm seed point diameter). Analysis was conducted per cell by manually selecting regions covering the individual cell and not overlapping with neighboring cells.

### Colocalization analysis

To assess endogenous Keap1 puncta colocalization with BODIPY puncta, we segmented each type of aggregates as sphere of variable size with an estimated diameter of 1 μm in the *xy* plane and 2 μm along the *z* axis, using the Spots module of Imaris (version 10.2), and performed spot-based colocalization. Spots were considered colocalized if their centers were up to 1 μm apart. We manually segmented each cell in 3D using the Surface module and quantified the number percentage and the total puncta volume percentage of colocalized spots for each cell.

### Fluorescence live-cell imaging

U2OS cells were seeded on a 35-mm WiLLCo-dish (WillCo Wells, HBST-3522) and incubated in growing conditions overnight. Before imaging, the cells were stained with LysoTracker red DND-99 (1:1,000; Thermo Fisher Scientific, L7528) for 30 min in growing conditions. Compound EL256 was added, followed by the start of imaging. Live-cell imaging was performed using a Lattice Lightsheet 7 microscope (Carl Zeiss) under controlled conditions (37 °C, 5% CO_2_). LysoTracker and EL256 signals were simultaneously captured in two channels with a dual-camera setup, using bandpass filters of 505–545 nm and 575–618 nm. Excitation was achieved with a combination of 488-nm and 561-nm lasers, using a Sinc3 15 × 650 light sheet. Imaging volumes were acquired with 1,001 slices at a 0.2-µm step size and a pixel size of 0.145 µm. Time-lapse recordings were taken at 30-min intervals over 24 h. Data processing, including deskewing, cropping and orthogonal projection, was performed in ZEN Blue 3.10 software, while final visualization and video export were completed using Imaris 10.2 (Bitplane). The LysoTracker signal was used as a reference for cell location and focus during imaging and analysis and its signal was not reported in this work.

### In vitro crosslinking using GA

Recombinant Keap1 samples were incubated at room temperature in desired conditions in a buffer containing 25 mM HEPES, 150 mM NaCl pH 8, 1 mM TCEP and 0.01% Tween. At the selected time point, each sample was treated with a 7.5% aqueous solution of GA (prepared fresh) for a final GA concentration of 0.125% and mixed gently. The crosslinking reaction was left at room temperature for 40 min, followed by addition of NuPage gel sample buffer with 20 mM DTT and boiling at 95 °C for 5 min. Lastly, 2–3 μg of each sample was loaded and run on an SDS–PAGE gel. Bands were visualized using a Coomassie blue solution.

### NQO2 enzyme activity assay

Assay ready plates were prepared by acoustically dispensing stock solutions of the test compounds, in a dose–response series (nine-point assay, threefold dilution, with 10 µM as the upper limit, in triplicate), into a black, low-volume 384-well plate (Greiner Bio-One) using an Echo 555 (Labcyte). The enzymatic activity of QR2 was measured as previously described^74^. Briefly, 7.5 µl of menadione alone (200 µM) or a mixture of menadione and QR2 (200 µM of menadione and 10 nM of QR2) was dispensed into the assay-ready plates, using a Multidrop Combi reagent dispenser (Thermo Fisher) and allowed to incubate with the compounds for 10 min. Dihydrobenzylnicotinamide (200 µM) was then added (7.5 µl) to the reaction and the fluorescence intensity (excitation, 360 nm; emission, 470 nm) was measured after 10 min of incubation in a PHERAstar FS multimode plate reader (BMG Labtech).

### Negative staining

Recombinant Keap1 BTB was incubated at a concentration of 50 µM with compound at indicated times, in a buffer containing 25 mM HEPES pH 8.0, 150 mM NaCl, 1 mM TCEP and 0.01% Tween, at room temperature. Then, samples were diluted tenfold and applied to glow-discharged carbon/formvar-coated grids (Electron Microscopy Sciences), stained with 2% uranyl acetate and imaged using a Tecnai T12 transmission electron microscope (Thermo Fisher Scientific) operated at an accelerating voltage of 120 kV and equipped with a TemCam-XF416 camera (TVIPS).

### Modeling Keap1–EL133 complex

We used AlphaFold3 to predict the structure of two Keap1 monomers in the presence of EL133. The input to AlphaFold3 included the amino acid sequence of Keap1 (explicitly specifying that the predicted complex should contain two monomers), a pregenerated 3D conformer of the reacted form of EL133 with ideal geometry and specification of the four atoms involved in covalent attachment (two PINCH atoms each form a bond with C151 of a different Keap1 monomer). A single seed was used, yielding five diffusion models. We present the top model according to the AlphaFold3 ranking score.

### Reporting summary

Further information on research design is available in the Nature Portfolio Reporting Summary linked to this article.

## Online content

Any methods, additional references, Nature Portfolio reporting summaries, source data, extended data, supplementary information, acknowledgements, peer review information; details of author contributions and competing interests; and statements of data and code availability are available at 10.1038/s41589-026-02141-0.

## Supplementary information

Supplementary Information Supplementary Figs. 1–14, synthetic procedures, analytical data and NMR spectra.

Reporting Summary

Supplementary Video 1 Lattice light-sheet live imaging of U2OS cells treated with EL256.

Supplementary Video 2 Lattice light-sheet live imaging of U2OS cells treated with EL256.

Supplementary Video 3 Lattice light-sheet live imaging of U2OS cells treated with EL256.

Supplementary Data 1 Proteomics data.

## Source data

Source Data Figs. 1, 2, 4 and 5 and Extended Data Figs. 1–3 Uncropped blots.

Source Data Figs. 1 and 3 and Extended Data Figs. 1 and 3 Raw data of graphs.

## Data Availability

All data associated with this study are available in the article and its Supplementary Information. Proteomics raw data were uploaded to the PRIDE repository with dataset identifier PXD067656 (Supplementary Data 1). Source data are provided with this paper.
