# Supplementary material for: A pharmacological modality to sequester homomeric proteins
Source: Nat Chem Biol. 2026 Feb 5;22(9):1414–24. doi: 10.1038/s41589-026-02141-0 (PMC13503280; doi:10.1038/s41589-026-02141-0)

nature chemical biology

Supplementary information

https://doi.org/10.1038/s41589-026-02141-0

A pharmacological modality to sequester homomeric proteins

In the format provided by the authors and unedited

Contents

A. Supplementary Figures
B. Synthetic procedures and analytical data
C. NMR spectra
D. Uncropped gels/blots from supplementary figures

A. Supplementary Figures

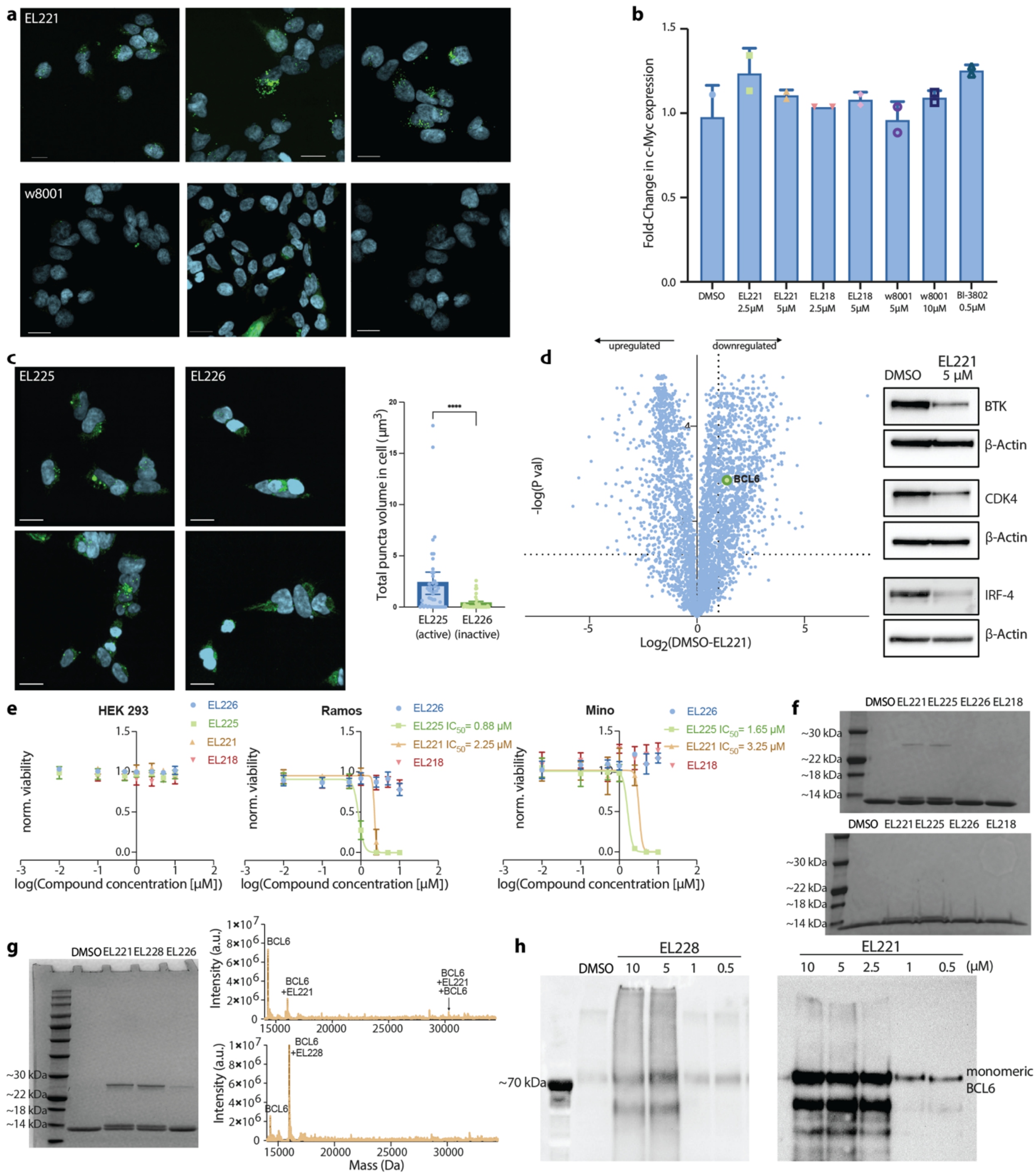

Supplementary Figure 1. a. Representative images of HEK 293 cells overexpressing BCL6-GFP(1-275), treated with w8001 (top) or EL221 (bottom), both 5 µM. Scale bars are 15 µM. **b.** rt-PCR analysis of c-Myc expression after a 20h treatment of Mino cells with compound (representative results following two independent experimental replicates with two technical replicates each). Data shows the mean and standard deviation. **c.** HEK 293 cells overexpressing with BCL6-GFP(1-275) show significantly higher ‘puncta’ formation following treatment with active PINCH EL225 (left) when compared to inactive

EL226 (right). Quantification performed on n=83 cells from a single experiment, $p<0.0001$ in a two-tailed Mann-Whitney test. Data shown as mean and 95% confidence interval. **d.** Global proteomics analysis of the cell's soluble lysate following treatment with 5 µM EL221, compared to DMSO after a 20 h treatment, showing BCL6 as one of the significantly downregulated targets. The dotted lines mark a 2-fold reduction in protein level (vertical) and p=0.05 significance in a double-sided student's t-test (horizontal). Proteins identified as downregulated in proteomics experiments were validated one time by WB under the same treatment conditions. **e.** Viability of different cell lines after 72 h treatment with active BCL6-PINCHs EL221 and EL225, and inactive PINCHs EL226 and EL218 (representative results from two independent experimental replicates; n=6 technical replicates each). Data shown as mean and standard deviation. **f.** SDS-PAGE gel showing samples of 10 µM recombinant BCL6-BTB incubated overnight at RT with indicated compounds at a concentration of 100 µM. Upper gel shows samples that were prepared in sample buffer and mildly heated to 37°C, bottom gel shows the same samples prepped with boiling to 95°C for 5 min prior to gel loading. Representative results from 2 independent experiments. **g.** SDS-PAGE gel showing samples of 10 µM recombinant BCL6-BTB, incubated for 3 days at RT with indicated compounds at a concentration of 100 µM (left). Samples in similar conditions show minimal covalent dimer detected by intact LC-MS (right). Representative results from 2 independent experiments. **h.** WB of insoluble cell lysates following BCL6-PINCHs treatment, stained with anti-BCL6 antibody, showing an intense band correlated with the mass of monomeric BCL6, and a 'smear' at higher MWs. Representative results from 3 independent experiments.

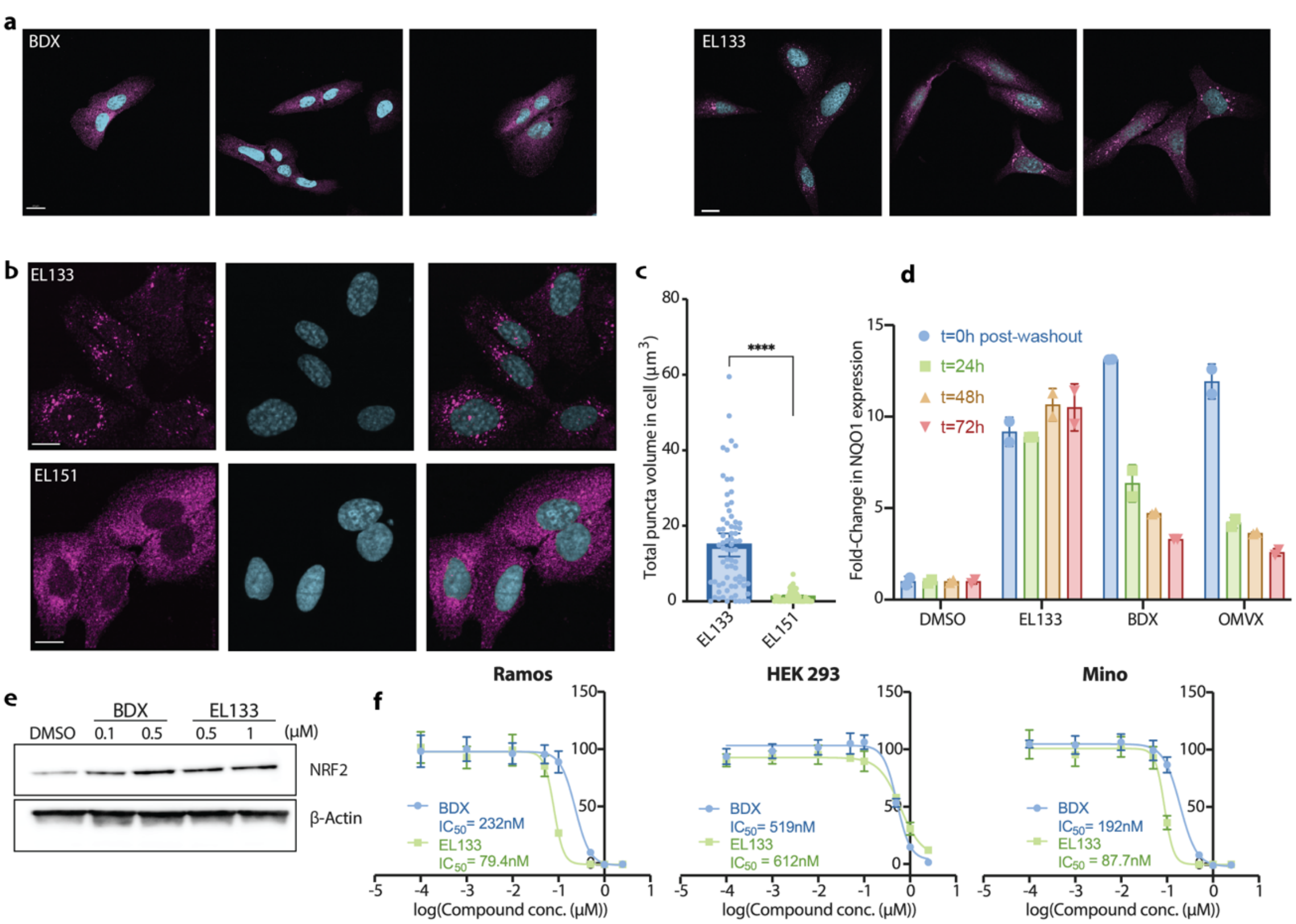

Supplementary Figure 2. a. Representative images of U-2OS cells treated with Bardoxolone (left) or EL133 (right), both at a concentration of 250 nM, immunostained with Keap1 antibody (Magenta). Scale bars are 10 µm. **b.** Immunostained U2OS cells show significantly higher 'puncta' formation following treatment with active PINCH EL133 (top) than with inactive EL151 (bottom) **c.** Quantification performed

on n=135 cells from two independent experiments, $p<0.0001$ in a two-tailed Mann-Whitney test. Data shown as mean and 95% confidence interval. **d.** qPCR analysis of NQO1 expression after a 20h treatment of OCI-AML2 cells with DMSO, EL133, BDX, or OMVX at 250 nM, followed by washing of the cells. Data shown as mean and standard deviation. Representative results following two independent experimental replicates. **e.** WB in U2OS cells against Nrf2 following 20 h compound treatment. Nrf2 levels increase following Keap1 inhibition by BDX, and Keap1 'PINCHing' by EL133. Representative results from 2 independent experiments. **f.** Viability of different cell lines after 72 h treatment with EL133 or BDX (n=6 technical replicates each). Data shown as mean and standard deviation.

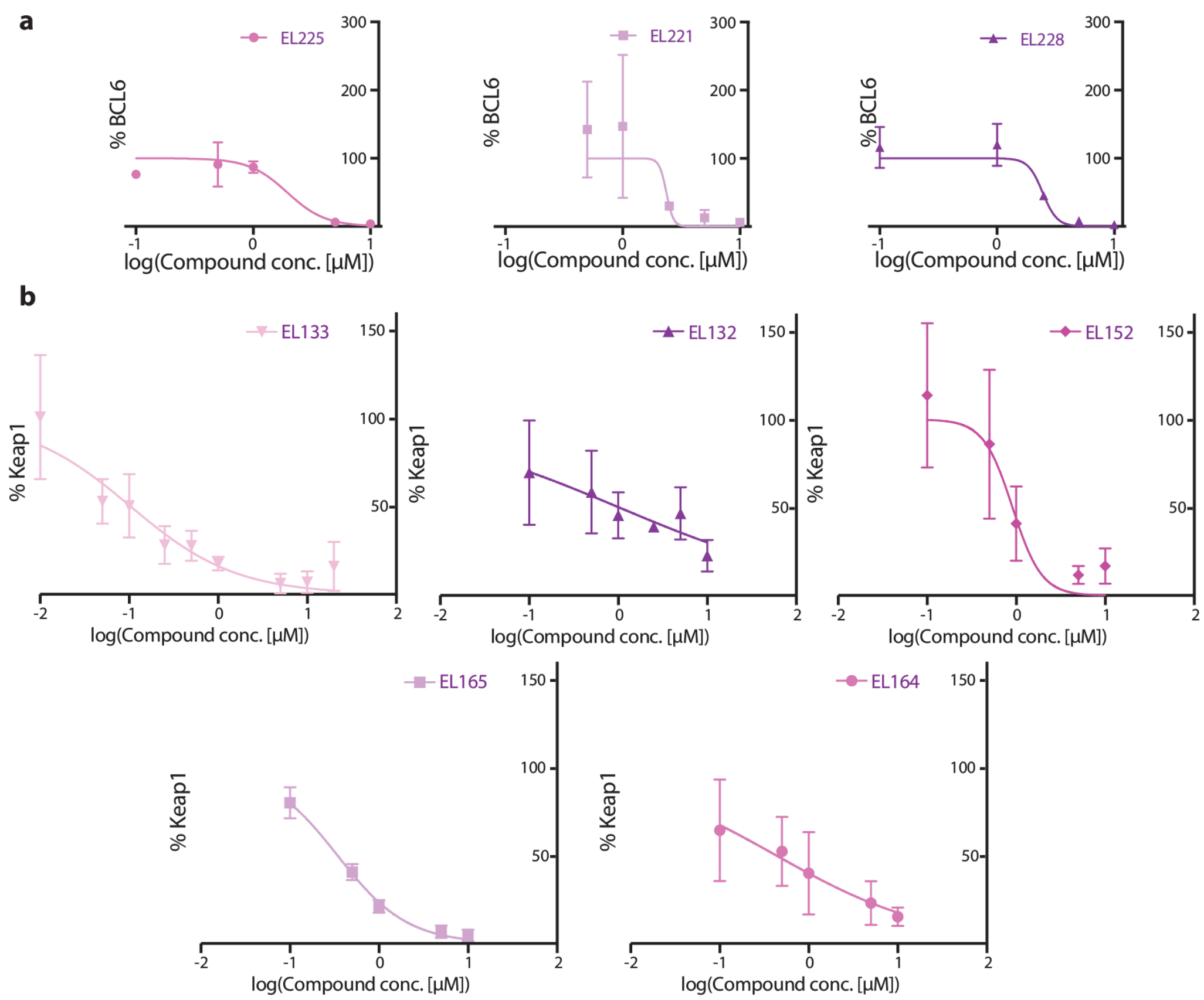

Supplementary Figure 3. WB primary band quantification and EC_{50} values of **a.** BCL6 PICNHs presented in Fig. 1 and **b.** Keap1 PINCHs of WBs presented in Fig. 2. data depicting mean and standard deviation values.

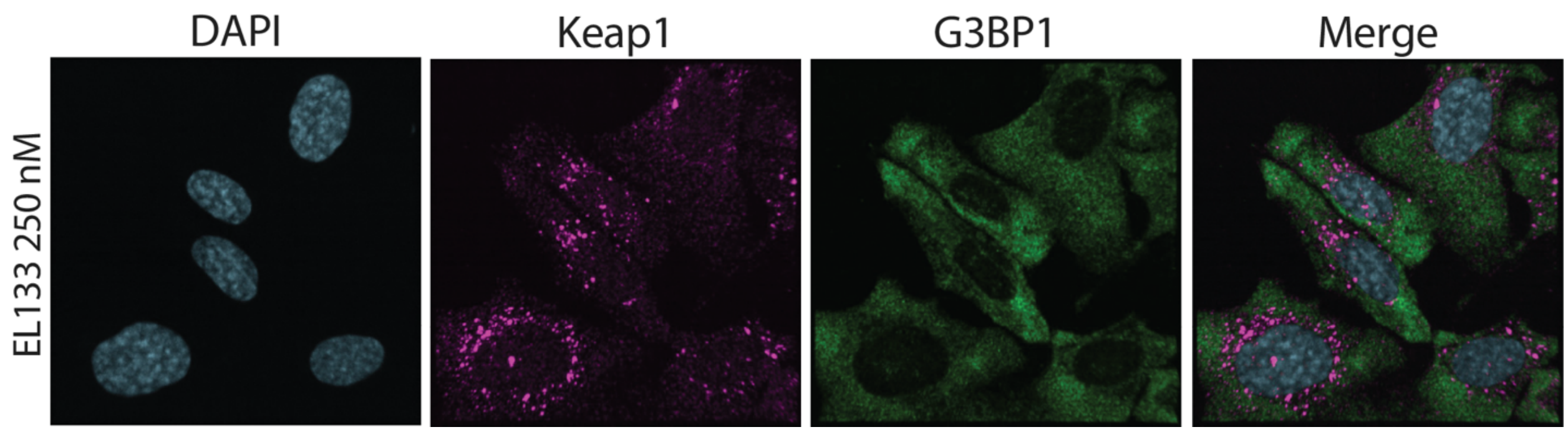

Supplementary Figure 4. Representative images of U2OS cells immunostained with Keap1 and G3BP1 (Stress granule formation marker), following a 20 h treatment with 250 nM EL133, showing expected Keap1 'puncta' but no local accumulation of G3BP1.

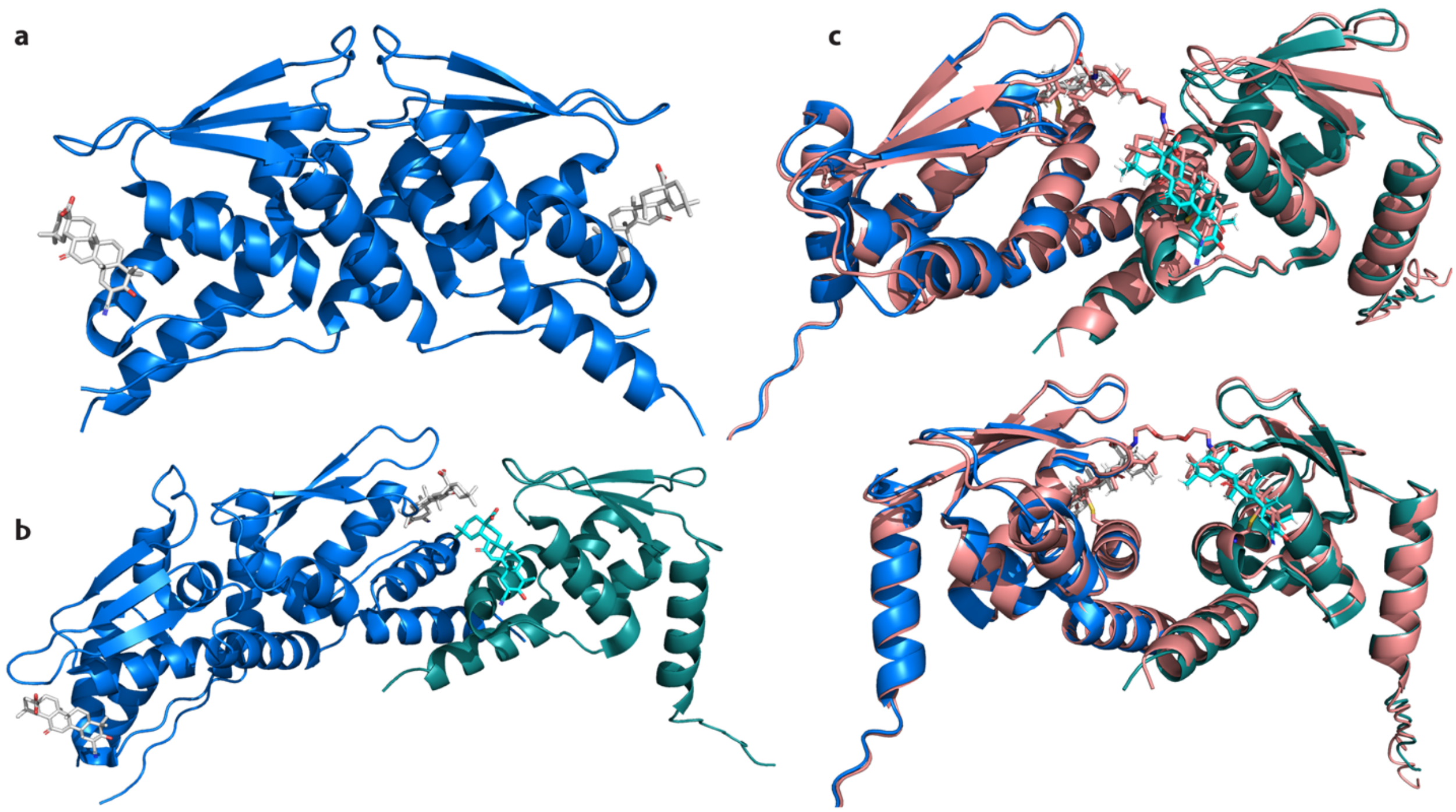

Supplementary Figure 5. a. Keap1-BTB biological homodimeric unit, in complex with Bardoxolone (in white sticks). **b.** Keap1-BTB biological homodimeric unit (blue), and an additional crystallographic symmetry mate monomeric Keap1-BTB (teal). **c.** AlphaFold3-generated structure of two Keap1 monomers bound covalently by EL133 (pink), overlaid onto the crystallographic non-native dimer (in blue and teal).

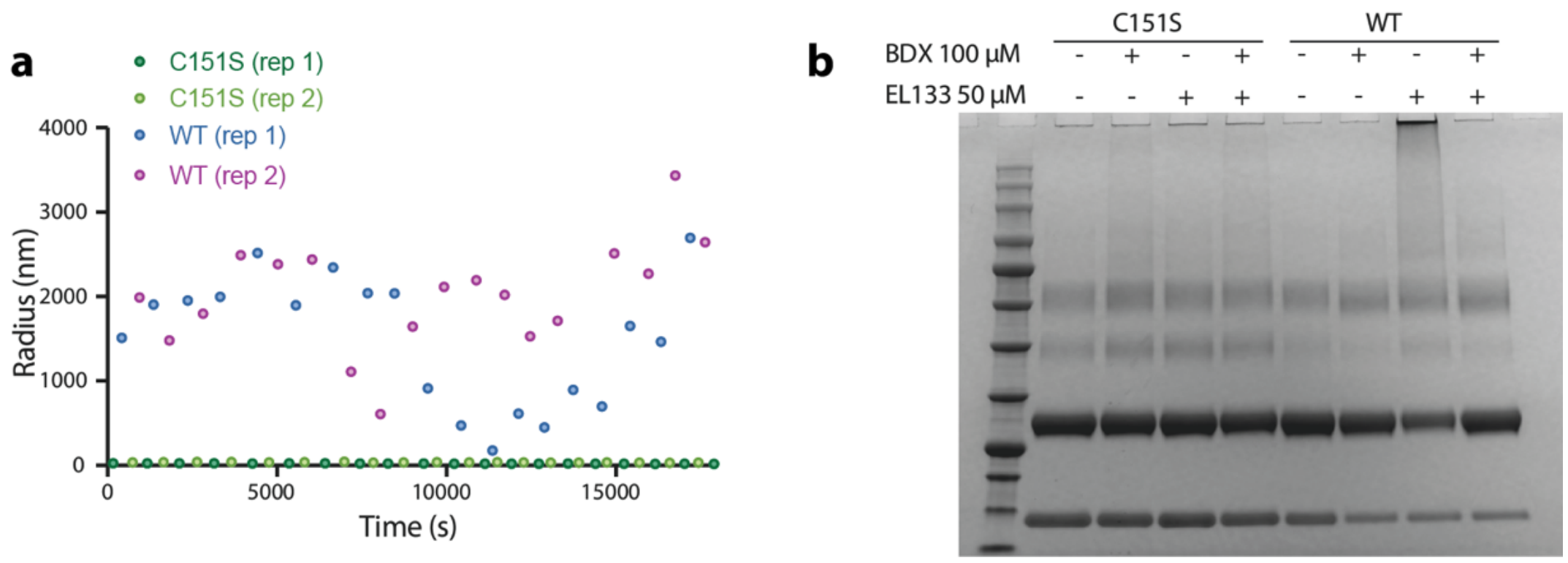

Supplementary Figure 6. a. Dynamic light scattering experiments (two independent repeats) following samples of 50 μM recombinant Keap1-BTB WT or C151S mutant, incubated with 50 μM EL133 over time at room temperature. **b.** Similar samples of 50 μM Keap1 WT/mutant incubated for 1 h at room temperature, with and without pre-incubation with excess BDX. Samples were crosslinked with GA, ran on an SDS-PAGE gel and stained with Coomassie blue. Representative results from 3 independent experiments.

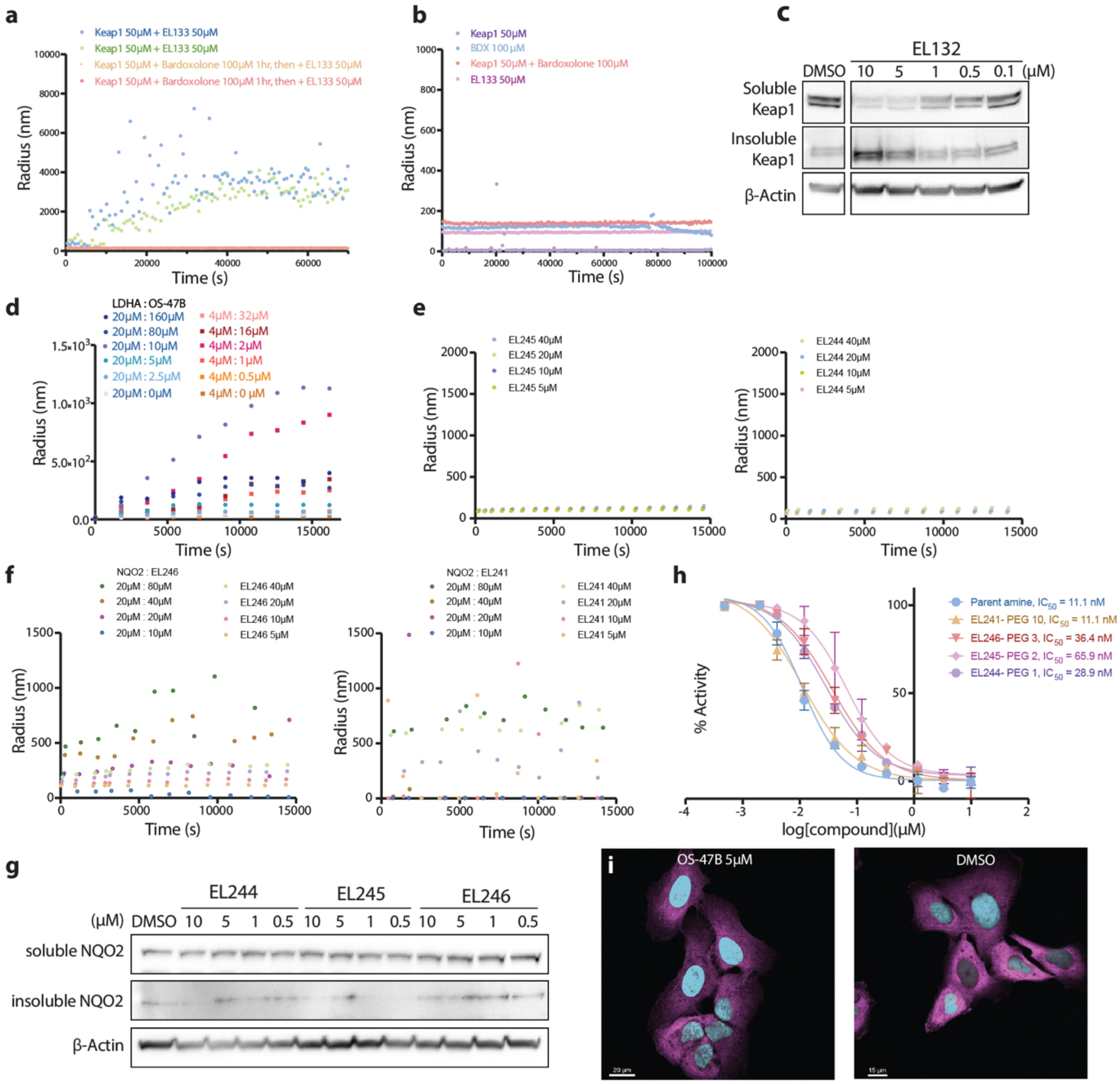

Supplementary Figure 7. a. Dynamic light scattering experiments following recombinant Keap1-BTB and EL133 over time at room temperature, with and without pre-incubation of the protein with excess BDX. **b.** Control DLS samples of Keap1/EL133/BDX alone in buffer, and of Keap1+BDX, showing minimal baseline signal. **c.** WB of EL132's effect on Keap1 in OCI-AML2 cells' soluble and insoluble phases; 20 h treatment. Representative results from 3 independent experiments. **d.** Dynamic light scattering experiments following recombinant LDHA and its PINCH at different concentration ratios over time at room temperature. Legends show concentrations indicate binding sites:binding moieties, similarly to Fig. 3. **e.** Dynamic light scattering experiments following EL245 (left) or EL244 (right) over time at room temperature, showing the compounds alone exhibit minimal baseline signal. **f.** Dynamic light scattering experiments following recombinant NQO2 and PINCHs EL246 and EL241 at different ratios over time at room temperature. Legends show concentrations indicate binding sites:binding moieties **g.** NQO2 PINCHs show no activity in U2OS cells; 3 h treatment, experiment performed one time. **h.** NQO2 PINCHs and parent amine inhibitor tested in an enzymatic activity assay, reflecting similar ability of target engagement for all compounds. Data shows the mean and standard deviation **i.** Representative immunostaining images of U2OS cells following 5μM OS-47B treatment for 20 hours, LDHA in Magenta.

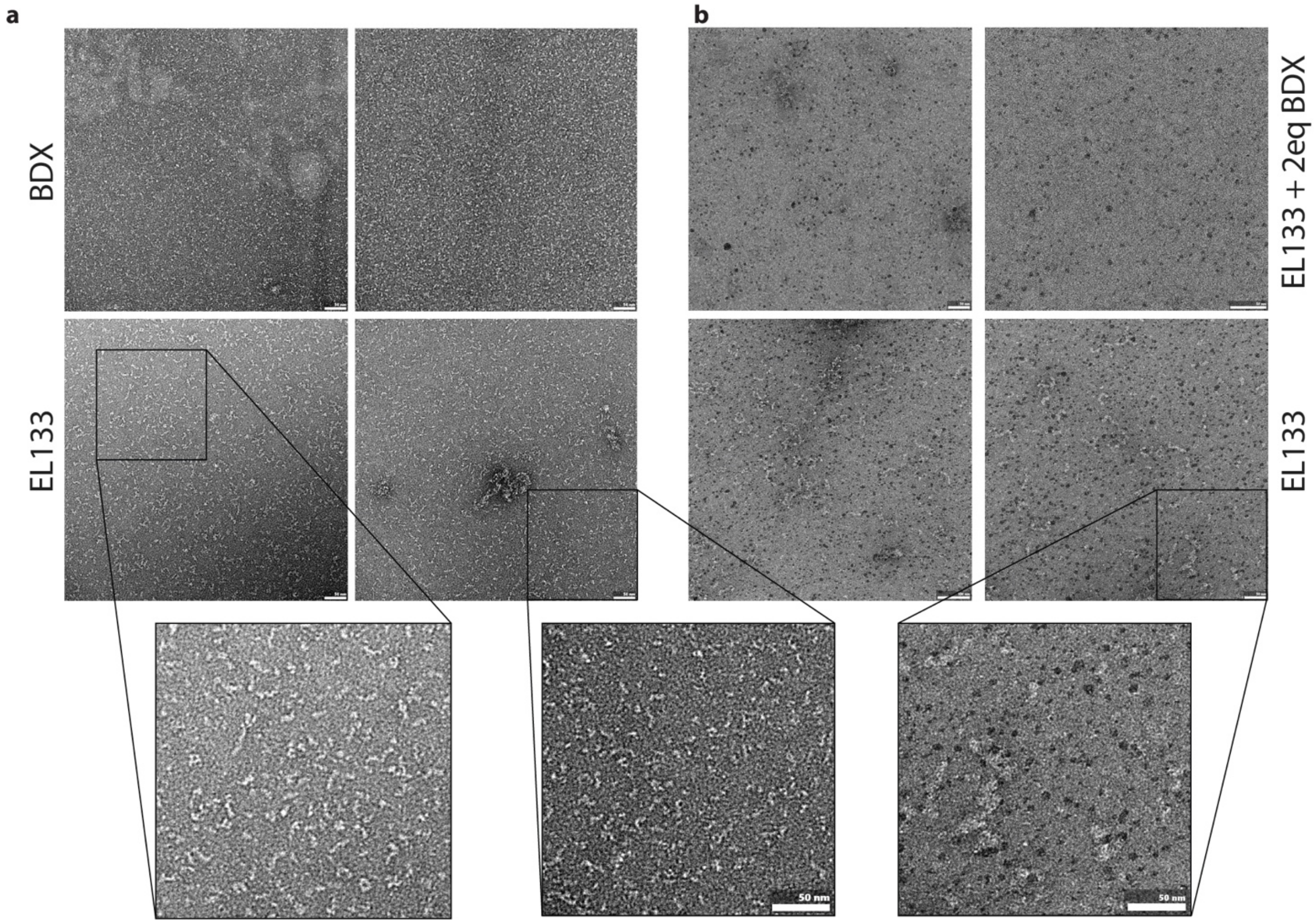

Supplementary Figure 8. a. Negative Stain images of 50 µM Keap1 incubated with 50 µM BDX/EL133 overnight at room temperature. **b.** Negative Stain images of 50 µM Keap1 incubated with 50 µM EL133 for 5 hours at room temperature, with and without pre-incubation of Keap1 with 100 µM BDX. Scale bars are 50 nm.

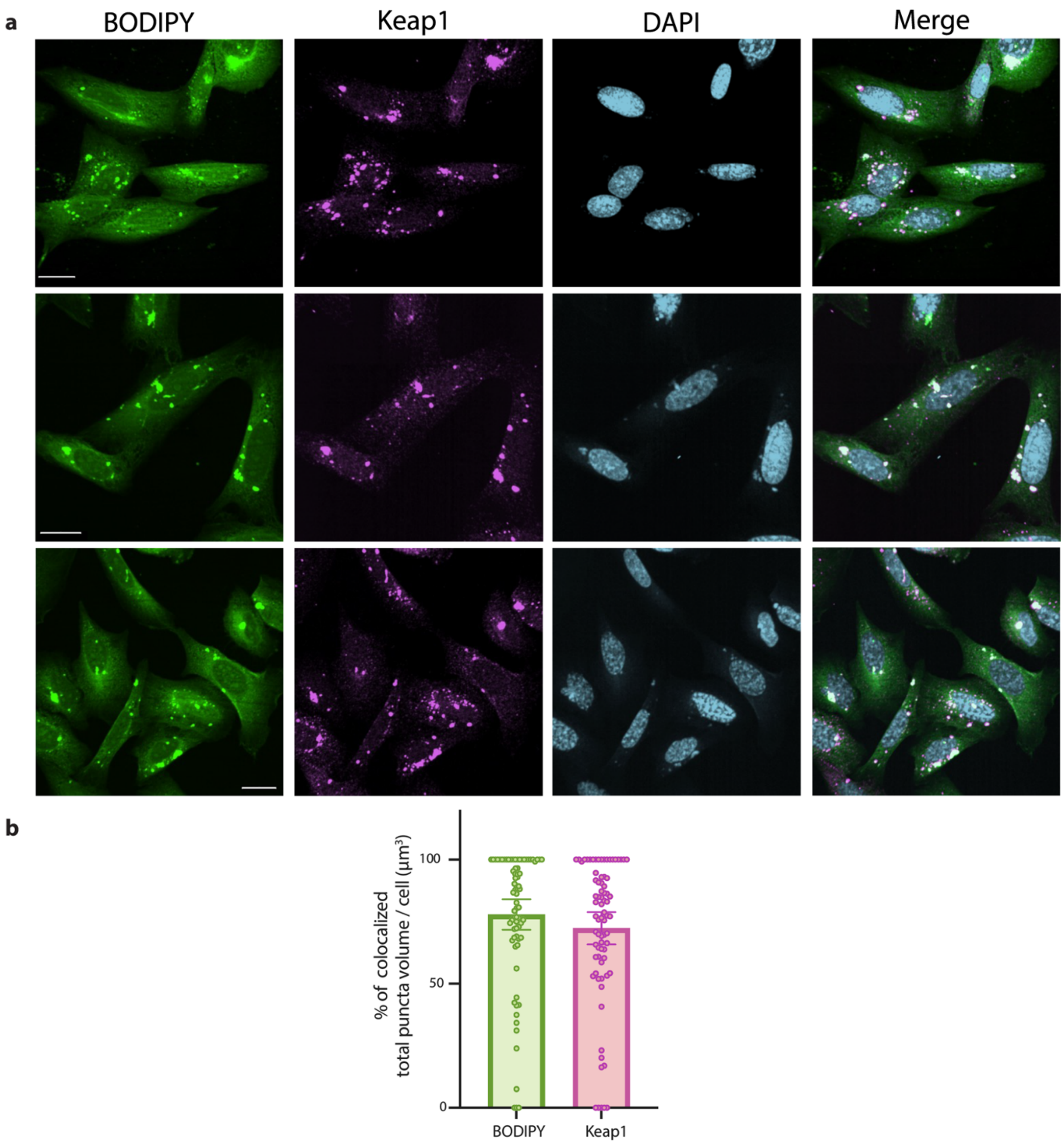

Supplementary Figure 9. a. Representative images of U2OS cells treated with 2.5 µM EL256 for 20 h, immunostained with Keap1 antibody (Magenta). The green signal is from the BODIPY-conjugated EL256. Scale bars = 20 µM. **b.** Quantification of co-localized puncta. In green is the % of BODIPY puncta that co-localized to Keap1 puncta and in pink is the % of Keap1 puncta that colocalized to BODIPY puncta (colocalized puncta/total puncta, for each signal), per cell. The bar chart indicates median values over n=75 cells total from two independent experiments, and the error bars represent a 95% confidence interval.

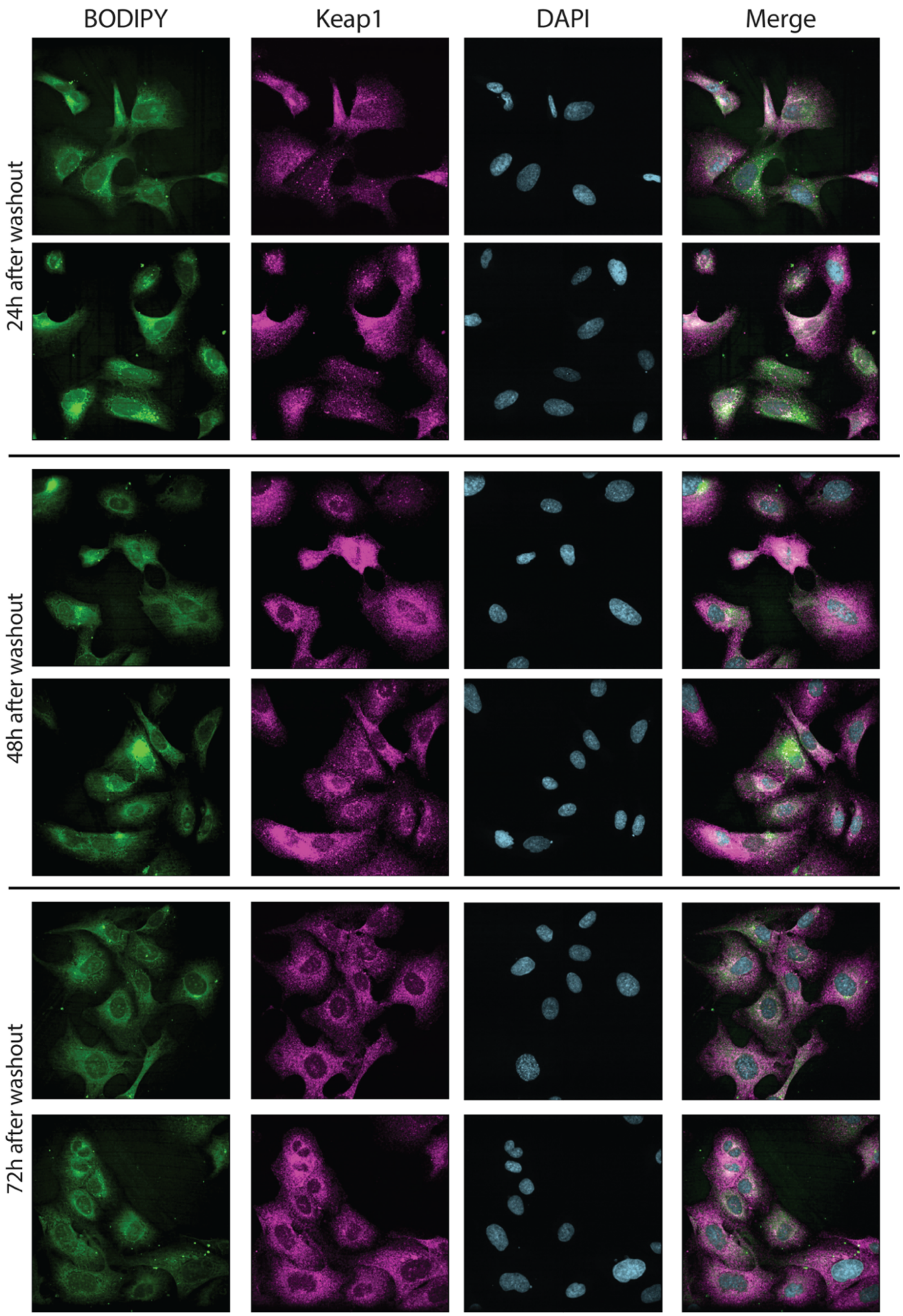

Supplementary Figure 10. Representative images of U2OS cells treated with 2.5 μM EL256 for 20h, then washed, fixed and stained at different times post-washout. Compare with Fig. 2h for puncta formation without washout.

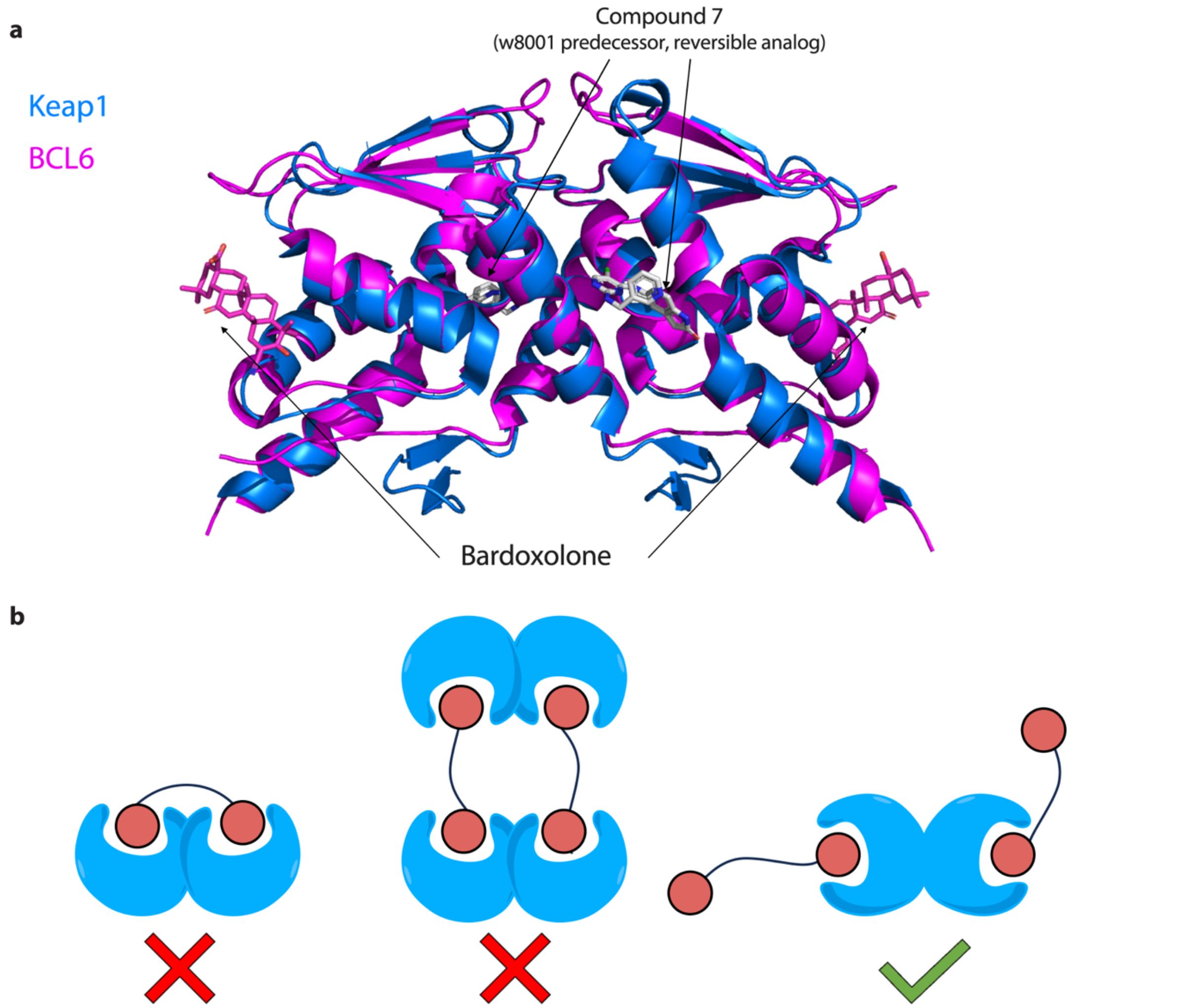

Supplementary Figure 11. a. Overlay of BTB domains of Keap1 (blue, PDB: 4CXT) and BCL6 (magenta, PDB: 5X4Q) and their respective ligands. Compound 7[1] is an early reversible BCL6 binder from which w8001's covalent analog was later optimized, and reflects our BCL6-PINCH binding site. This shows the ligands bind distinct pockets on the BTB domain. However, in both cases, the pockets are on opposite sides of the dimer (left and right for Keap1, front and back for BCL6) **b.** Geometric constraints for effective polymerization by PINCHs. If the binding sites are too close to one another (left panel) or facing the same side of the multimer (middle panel) they may result in a closed oligomer formation. If the binding sites are on opposite sides (right panel) as they are in Keap1 and BCL6 (panel A), they should lead to effective polymerization.

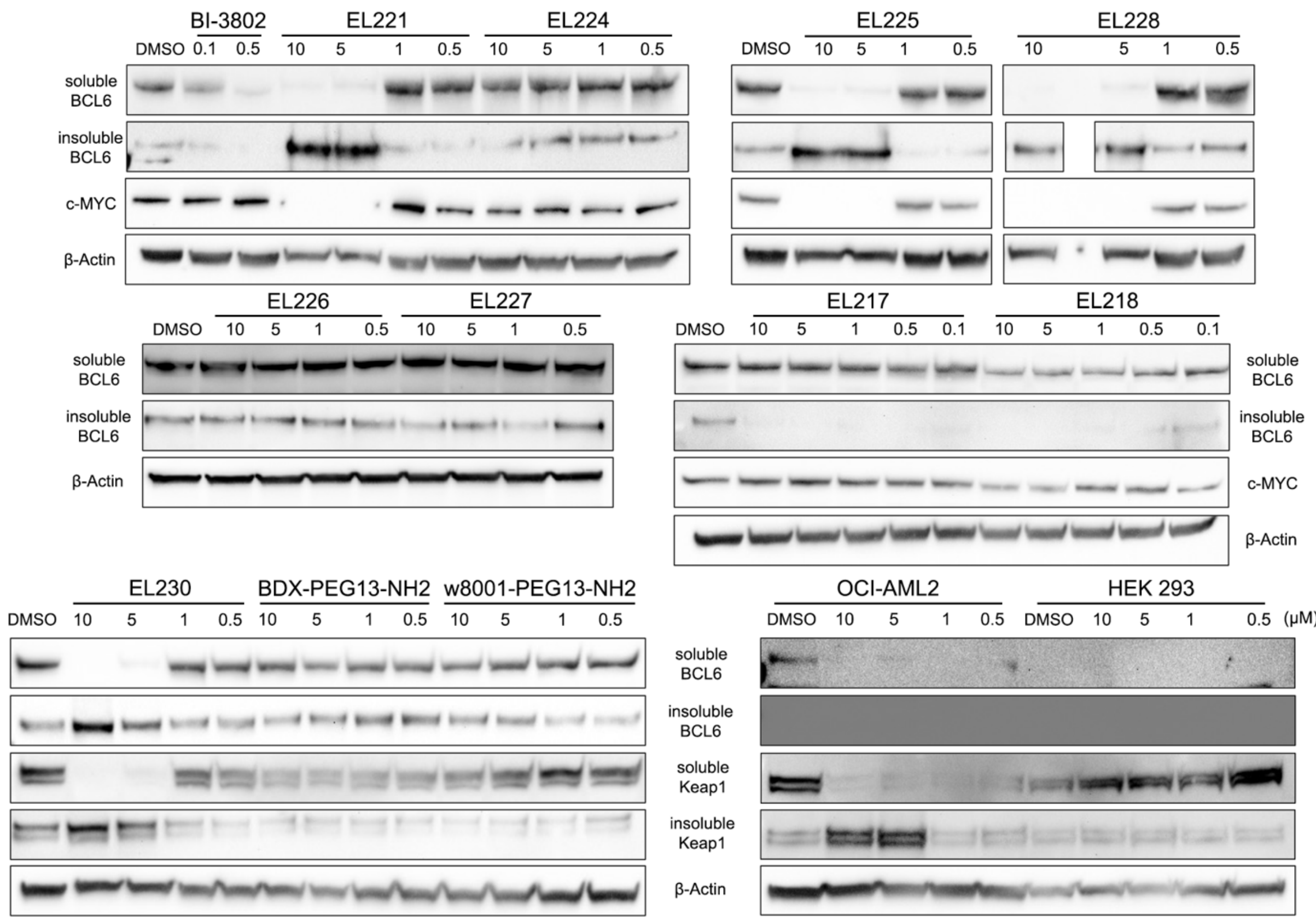

Supplementary Figure 12. Representative repeats of the WBs shown in figures 1, 5 and extended data figure 1

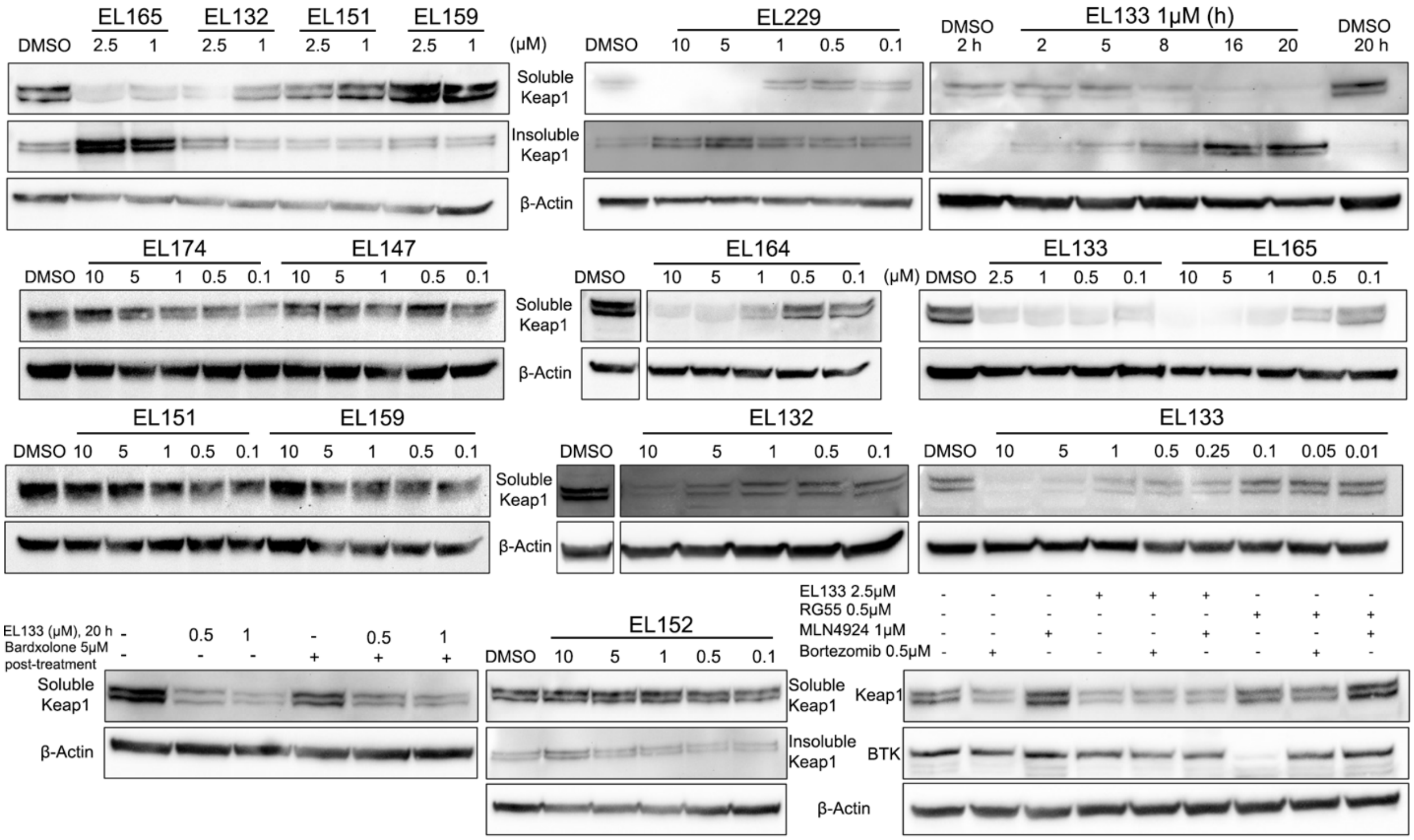

Supplementary Figure 13. Representative repeats of the WBs shown in figures 2, 4 and extended data figure 2.

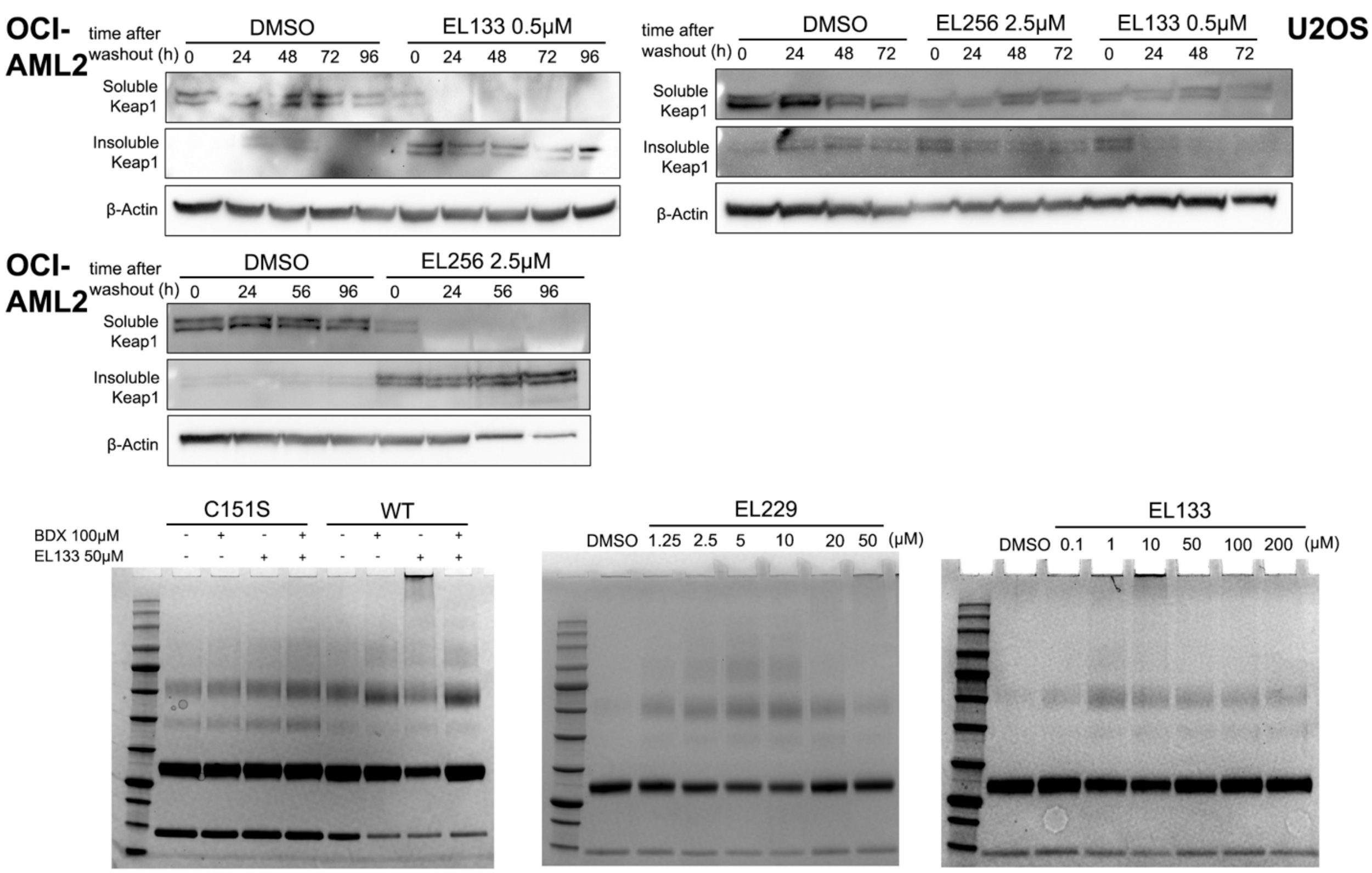

Supplementary Figure 14. Representative repeats of the WBs and gels shown in extended data figure 3 and Fig. S6

B. Synthetic procedures and analytical data

Synthetic procedures and analytical data

w8001 (1)- 3-(((4-chloro-3-((8-methoxy-1-methyl-3-(2-(methylamino)-2-oxoethoxy)-2-oxo-1,2-dihydroquinolin-6-yl)amino)phenyl)amino)methyl)benzenesulfonyl fluoride

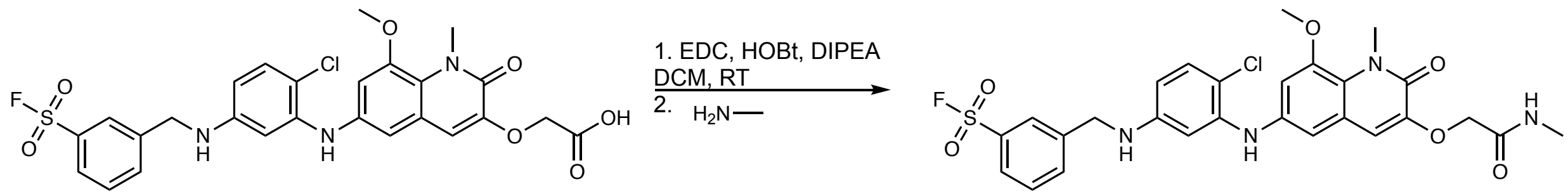

w8002 free acid (synthesized by WuXi) was reacted with 2 eq EDC and 2 eq HOBt, along with 1.1 eq DIPEA in DCM for 15 min in room temperature. Next, 1 eq of methylamine was added to the reaction mixture and allowed to react for 3 h in room temperature. The crude reaction was evaporated, dissolved in 30% ACN in H_2O, and purified by HPLC.

HR-MS (m/z): Calculated: 588.12; Found: 611.1154 $[M+Na]^+$

^{1}H NMR of starting material, as measured by WuXi:

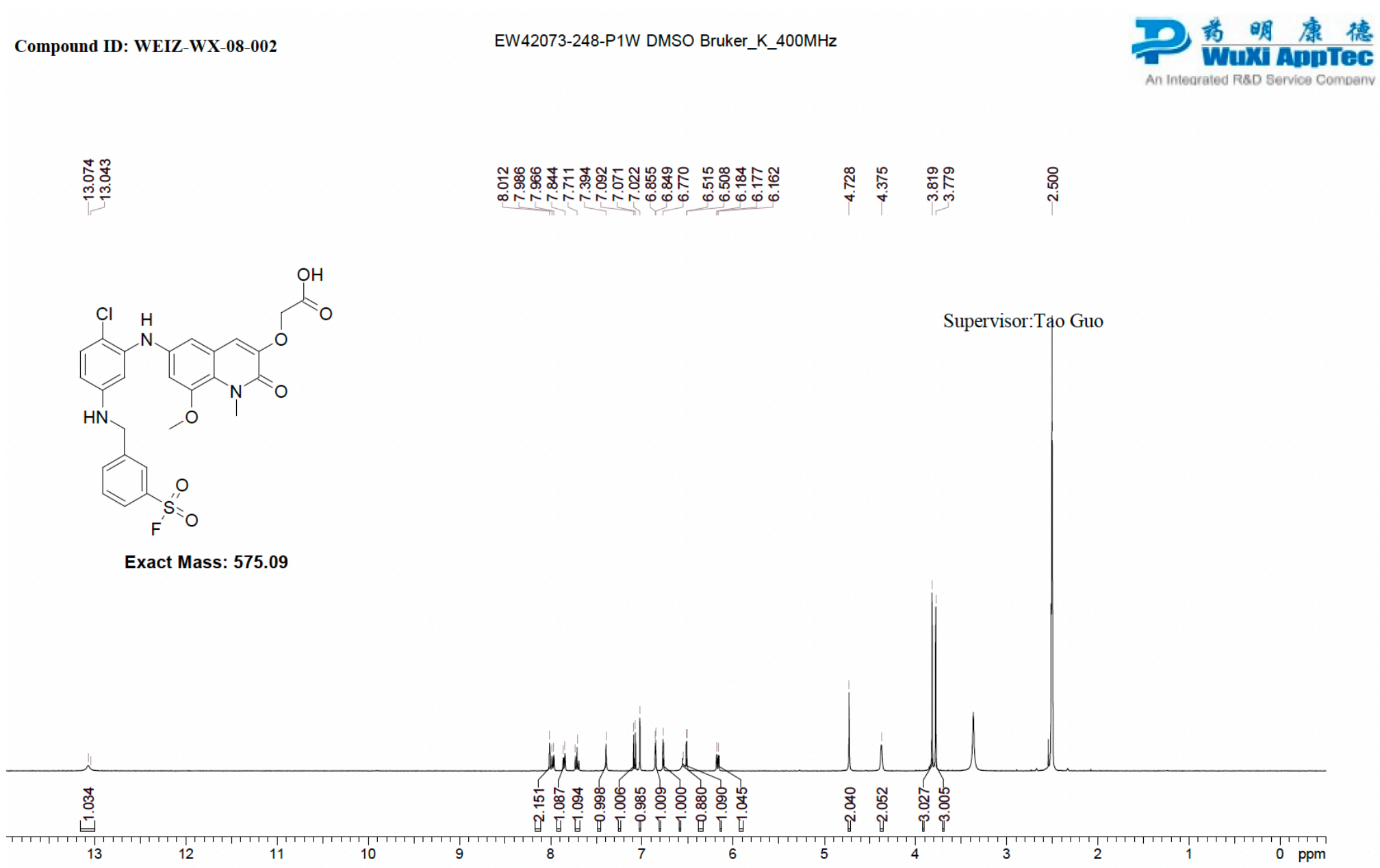

EL221 (2)- 3,3'-(((((((2,46-dioxo-6,9,12,15,18,21,24,27,30,33,36,39,42-tridecaoxa-3,45-diazaheptatetracontane-1,47-diyl)bis(oxy))bis(8-methoxy-1-methyl-2-oxo-1,2-dihydroquinoline-3,6-diyl))bis(azanediyl))bis(4-chloro-3,1-phenylene))bis(azanediyl))bis(methylene))dibenzenesulfonyl fluoride

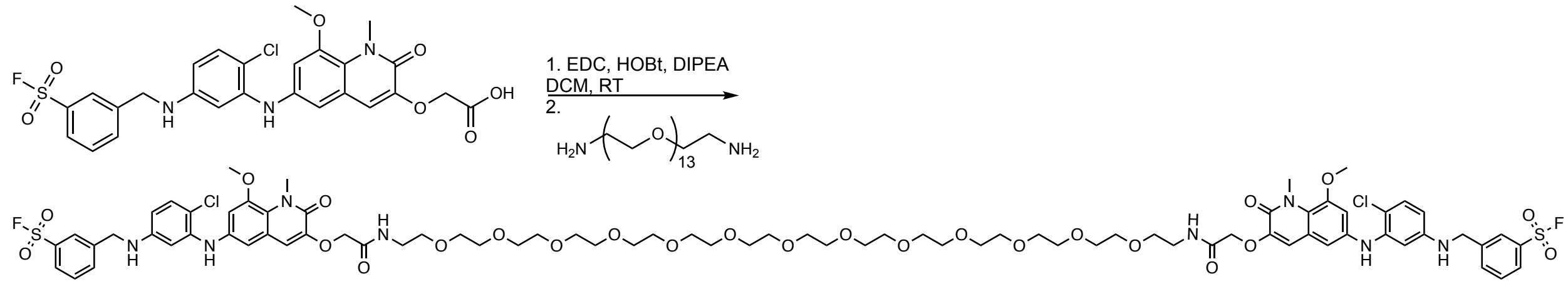

w8002 free acid was reacted with 2 eq EDC and 2 eq HOBt, along with 1.1 eq DIPEA in DCM for 15 min in room temperature. Next, 3,6,9,12,15,18,21,24,27,30,33,36,39-tridecaoxahentetracontane-1,41-diamine was dissolved in 100μL DMF, then added to the reaction mixture and allowed to react for 3 h in room temperature. The crude reaction was evaporated, dissolved in 40% ACN in H_2O, and purified by HPLC.

^{1}H NMR (500 MHz, DMSO) δ 8.45 (s, 1H), 8.03 – 7.95 (m, 1H), 7.86 (d, *J* = 7.9 Hz, 1H), 7.74 – 7.69 (m, 1H), 7.41 (s, 1H), 7.10 – 7.06 (m, 2H), 7.05 (s, 1H), 6.87 (s, 1H), 6.75 (s, 1H), 6.53 (d, *J* = 10.4 Hz, 2H), 6.18 (d, *J* = 6.7 Hz, 2H), 4.54 (s, 3H), 4.38 (d, *J* = 5.5 Hz, 2H), 3.83 (s, 3H), 3.78 (s, 3H), 3.48 (s, 28H).

^{13}C NMR (126 MHz, DMSO) δ 167.62, 158.06, 158.06, 148.93, 148.93, 148.35, 146.86, 146.86, 143.87, 141.00, 139.10, 135.58, 135.58, 130.92, 130.49, 127.14, 126.83, 122.77, 121.37, 114.42, 110.70, 108.60, 106.67, 105.29, 102.76, 70.22, 70.03, 69.30, 56.94, 46.16, 38.78, 35.44.

HR-MS (m/z): Calculated: 1746.57; Found: 1747.5796 $[M+H]^+$, 1769.5701 $[M+Na]^+$

EL228 (3)- 3,3'-(((((((2,43-dioxo-6,9,12,15,18,21,24,27,30,33,36,39-dodecaoxa-3,42-diazatetratetracontane-1,44-diyl)bis(oxy))bis(8-methoxy-1-methyl-2-oxo-1,2-dihydroquinoline-3,6-diyl))bis(azanediyl))bis(4-chloro-3,1-phenylene))bis(azanediyl))bis(methylene))dibenzenesulfonyl fluoride

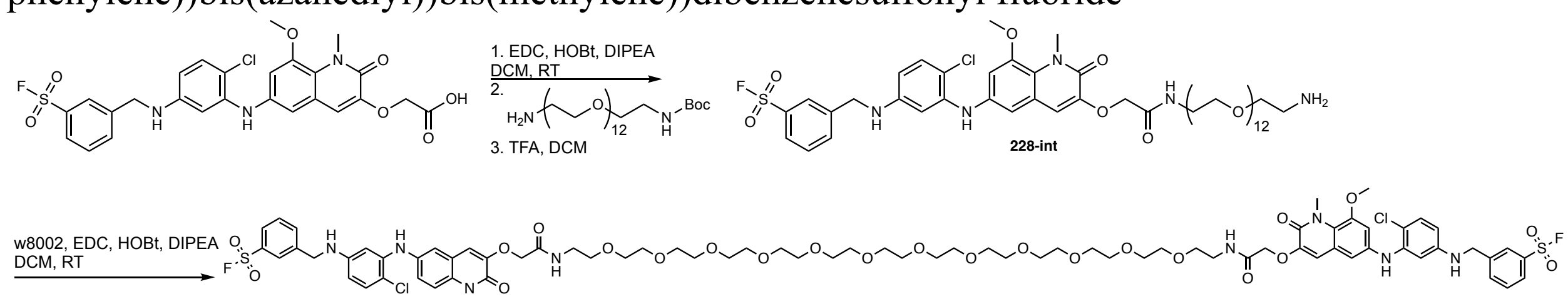

w8002 free acid was reacted with 2 eq EDC and 2 eq HOBt, along with 1.1 eq DIPEA in DCM for 15 min in room temperature. Next, *tert-butyl (38-amino-3,6,9,12,15,18,21,24,27,30,33,36-dodecaoxaoctatriacontyl)carbamate* was added to the reaction mixture and allowed to react for 3 h in room temperature. TFA was then added to double the solvent volume and left to deprotect overnight. The deprotected intermediate **228-int** was then evaporated and purified by HPLC, lyophilized and was then used directly in the following reaction. w8002 was reacted with EDC, HOBt and DIPEA as described in the first step. The intermediate amine was dissolved in 100 μL

DMF and added to the reaction mixture. After 2 h, the crude reaction was evaporated, dissolved in 40% ACN in H_2O, and purified by HPLC.

HR-MS (m/z): Calculated: 1702.55; Found: 1725.5461 $[M+Na]^+$

EL225 (4)- 3,3'-(((((((2,40-dioxo-6,9,12,15,18,21,24,27,30,33,36-undecaoxa-3,39-diazahentetracontane-1,41-diyl)bis(oxy))bis(8-methoxy-1-methyl-2-oxo-1,2-dihydroquinoline-3,6-diyl))bis(azanediyl))bis(4-chloro-3,1-phenylene))bis(azanediyl))bis(methylene))dibenzenesulfonyl fluoride

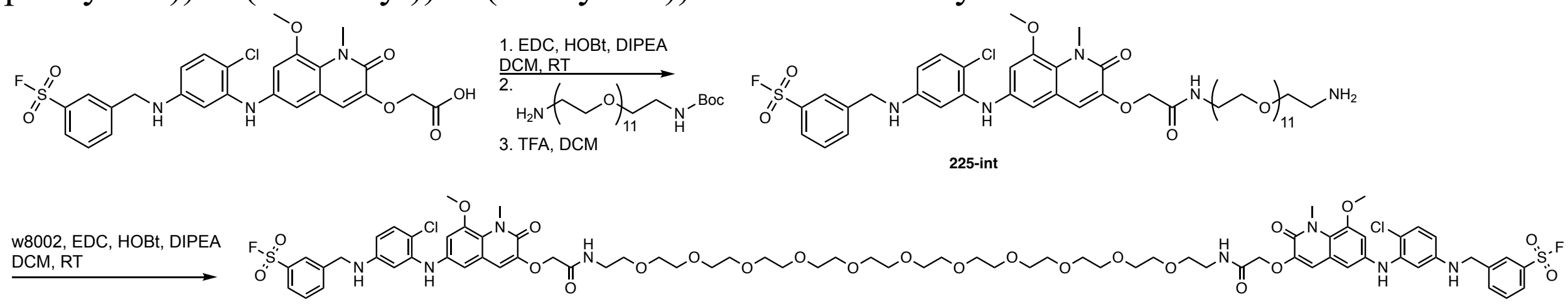

Synthetic procedure performed similar to the one described for EL228, using *tert-butyl (35-amino-3,6,9,12,15,18,21,24,27,30,33-undecaoxapentatriacontyl)carbamate* as the linker in the second step.

^{1}H NMR (500 MHz, DMSO) δ 8.47 (s, 1H), 8.01 (s, 1H), 7.99 – 7.96 (m, 1H), 7.86 (d, J = 7.7 Hz, 1H), 7.72 (t, J = 7.8 Hz, 1H), 7.41 (s, 1H), 7.08 (d, J = 8.7 Hz, 2H), 7.05 (s, 1H), 6.87 (d, J = 1.9 Hz, 1H), 6.75 (d, J = 1.9 Hz, 1H), 6.56 – 6.53 (m, 1H), 6.52 (d, J = 2.4 Hz, 1H), 6.18 (dd, J = 8.7, 2.3 Hz, 1H), 4.54 (s, 2H), 4.38 (d, J = 5.8 Hz, 2H), 3.82 (d, J = 5.4 Hz, 2H), 3.78 (s, 3H), 3.48 (s, 22H).

^{13}C NMR (126 MHz, DMSO) δ 167.63, 158.06, 148.94, 148.40, 146.86, 143.88, 141.00, 139.11, 135.58, 130.92, 130.52, 127.14, 126.83, 122.77, 121.36, 114.42, 110.70, 108.59, 106.67, 105.29, 102.77, 70.21, 70.15, 70.03, 69.30, 68.24, 56.94, 46.16, 38.78, 35.44.

HR-MS (m/z): Calculated: 1658.52; Found: 1681.5098 $[M+Na]^+$

EL229 (5)- (4a*S*,4a'*S*,6a*R*,6b*S*,6a'*R*,6b'*S*,8a*R*,8a'*R*,12a*S*,12a'*S*,14a*R*,14b*S*,14a'*R*,14b'*S*)-*N*a,*N*a'-(3,6,9,12,15,18,21,24,27,30,33,36,39-tridecaoxahentetracontane-1,41-diyl)bis(11-cyano-2,2,6a,6b,9,9,12a-heptamethyl-10,14-dioxo-1,3,4,5,6,6a,6b,7,8,8a,9,10,12a,14,14a,14b-hexadecahydropicene-4a(2*H*)-carboxamide)

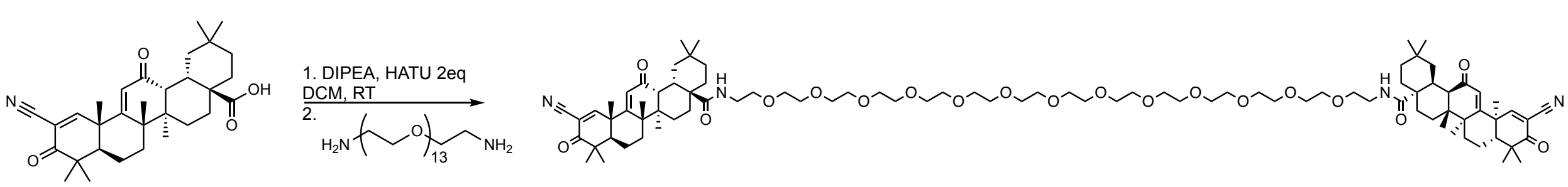

HATU-activation of Bardoxolone: 5 mg of Bardoxolone-COOH (CAS 218600-44-3, Labseeker) were dissolved in DCM, followed by 4eq of DIPEA and 2eq of HATU, and left stirring overnight at room temperature. Then, saturated NH_4Cl solutions was added to the reaction vile to approximately double the volume, followed by 3 times extraction of the activated Bardoxolone acid using DCM. The DCM was then evaporated, and the product dissolved in 200 µL DMF.

To the activated Bardoxolone, 5 eq of DIPEA were added, followed by addition of 3,6,9,12,15,18,21,24,27,30,33,36,39-tridecaoxahentetracontane-1,41-diamine (PEG13-diamine), and dissolved in DCM. After overnight reaction in room temperature, the solvent was evaporated, the crude reaction was then dissolved in 50% ACN in H_2O and purified using HPLC.

^{1}H NMR (500 MHz, DMSO) δ 8.65 (s, 1H), 7.79 – 7.69 (m, 1H), 6.20 (s, 1H), 3.53 – 3.47 (m, 30H), 3.43 – 3.39 (m, 3H), 3.17 – 3.09 (m, 2H), 3.02 (dd, *J* = 22.9, 4.5 Hz, 1H), 2.89 – 2.81 (m, 1H), 1.91 – 1.79 (m, 2H), 1.65 (d, *J* = 7.6 Hz, 3H), 1.58 – 1.52 (m, 3H), 1.43 (s, 3H), 1.24 (d, *J* = 6.9 Hz, 3H), 1.17 (d, *J* = 5.4 Hz, 3H), 1.06 (d, *J* = 7.6 Hz, 3H), 0.93 (s, 3H), 0.89 (s, 3H), 0.86 (s, 3H).

^{13}C NMR (126 MHz, DMSO) δ 200.00, 197.66, 177.15, 169.25, 168.65, 123.87, 115.61, 113.27, 70.25, 70.00, 69.50, 48.93, 45.96, 45.86, 45.81, 45.54, 44.84, 43.02, 41.99, 41.68, 39.23, 36.01, 34.69, 33.75, 31.24, 30.73, 30.70, 27.92, 26.58, 26.33, 24.71, 24.25, 23.61, 23.29, 22.33, 21.77, 21.58.

HR-MS (m/z): Calculated: 1579.00; Found: 1601.9918 [M+Na]+.

EL147 (6)- (4a*S*,4a'*S*,6a*R*,6b*S*,6a'*R*,6b'*S*,8a*R*,8a'*R*,12a*S*,12a'*S*,14a*R*,14b*S*,14a'*R*,14b'*S*)-*N*a,*N*a'-(oxybis(ethane-2,1-diyl))bis(11-cyano-2,2,6a,6b,9,9,12a-heptamethyl-10,14-dioxo-1,3,4,5,6,6a,6b,7,8,8a,9,10,12a,14,14a,14b-hexadecahydropicene-4a(2*H*)-carboxamide)

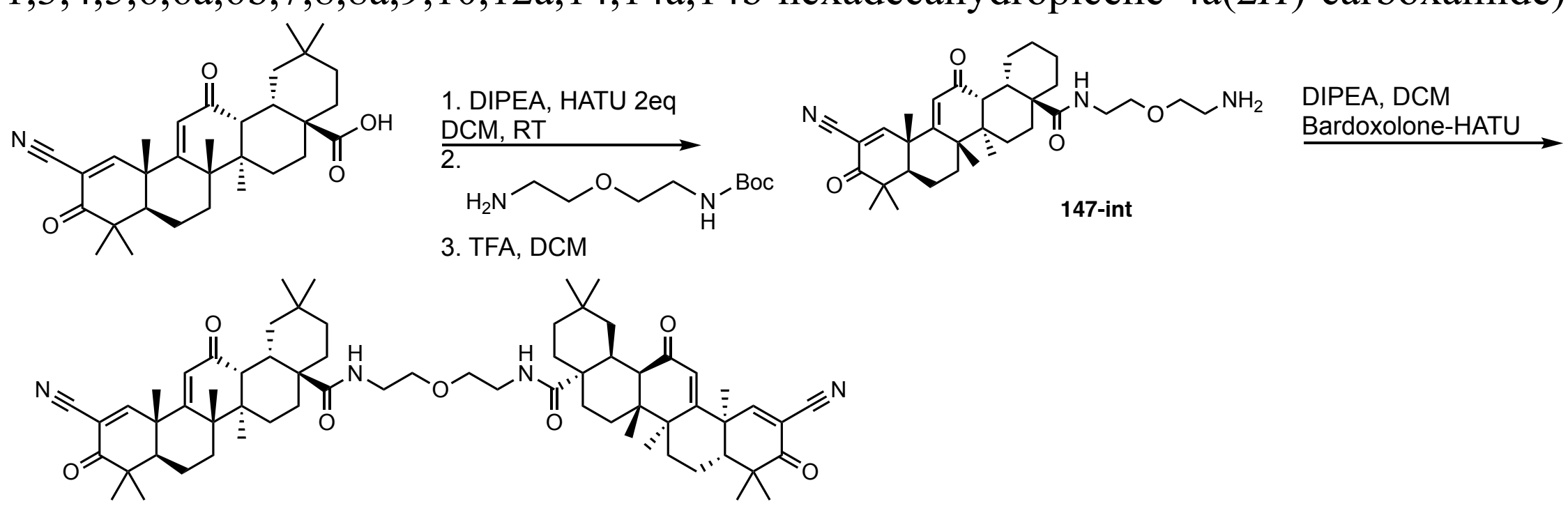

Bardoxolone-COOH was activated using HATU according to the synthetic procedure described for compound EL229, and it was reacted with *tert-butyl (2-(2-aminoethoxy)ethyl)carbamate* in DCM overnight. TFA was added to the reaction to obtain a final solvent ratio of approximately 1:2 (TFA:DCM) and the reaction was left overnight at room temperature. The deprotected intermediate **147-int** was then evaporated and purified by HPLC, lyophilized and was then used directly in the following reaction. Bardoxolone-HATU was added to the intermediate amine with DIPEA in DCM. After 6h, the reaction was evaporated and the final product was purified using HPLC.

HR-MS (m/z): Calculated: 1050.68; Found: 1051.6923 [M+H]$^+$, 1073.6742 [M+Na]$^+$

EL151 (7)- (4a*S*,6a*R*,6b*S*,8a*R*,12a*S*,14a*R*,14b*S*)-11-cyano-*N*-(1-((4a*S*,6a*R*,6b*S*,8a*R*,12a*S*,14a*R*,14b*S*)-11-cyano-2,2,6a,6b,9,9,12a-heptamethyl-10,14-dioxo-1,3,4,5,6,6a,6b,7,8,8a,9,10,12a,14,14a,14b-hexadecahydropicen-4a(2*H*)-yl)-1-oxo-5,8,12-trioxa-

2-azatetradecan-14-yl)-2,2,6a,6b,9,9,12a-heptamethyl-10,14-dioxo-1,3,4,5,6,6a,6b,7,8,8a,9,10,12a,14,14a,14b-hexadecahydropicene-4a(2*H*)-carboxamide

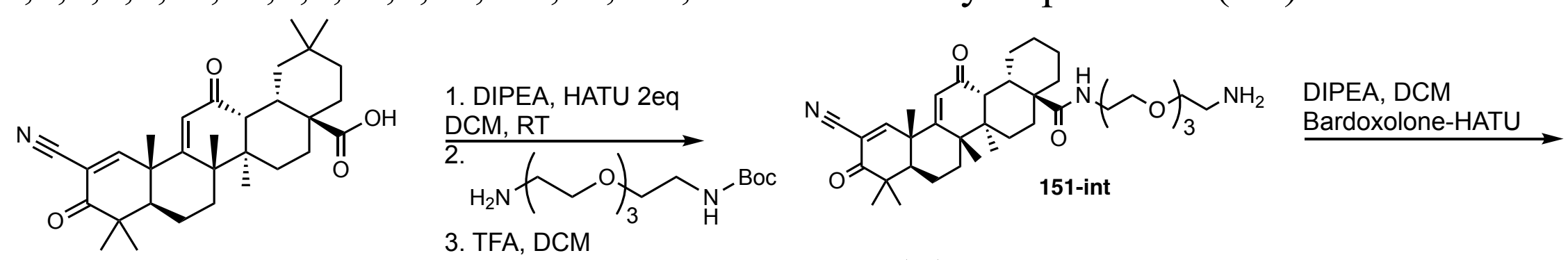

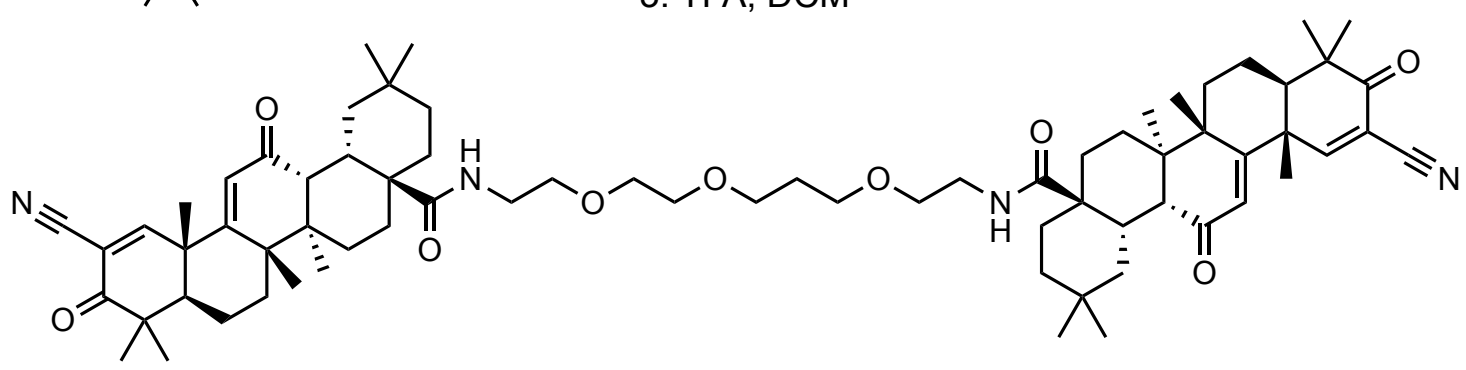

Synthetic procedure performed similar to the one described for EL147, using tert-butyl (2-(2-(3-(2-aminoethoxy)propoxy)ethoxy)ethyl)carbamate as the linker in the second step.

HR-MS (m/z): Calculated: 1138.73; Found: 1139.7429 $[M+H]^+$, 1161.7247 $[M+Na]^+$

EL133 (8)- (4a*S*,4a'*S*,6a*R*,6b*S*,6a'*R*,6b'*S*,8a*R*,8a'*R*,12a*S*,12a'*S*,14a*R*,14b*S*,14a'*R*,14b'*S*)-*N*a,*N*a'-((ethane-1,2-diylbis(oxy))bis(ethane-2,1-diyl))bis(11-cyano-2,2,6a,6b,9,9,12a-heptamethyl-10,14-dioxo-1,3,4,5,6,6a,6b,7,8,8a,9,10,12a,14,14a,14b-hexadecahydropicene-4a(2*H*)-carboxamide)

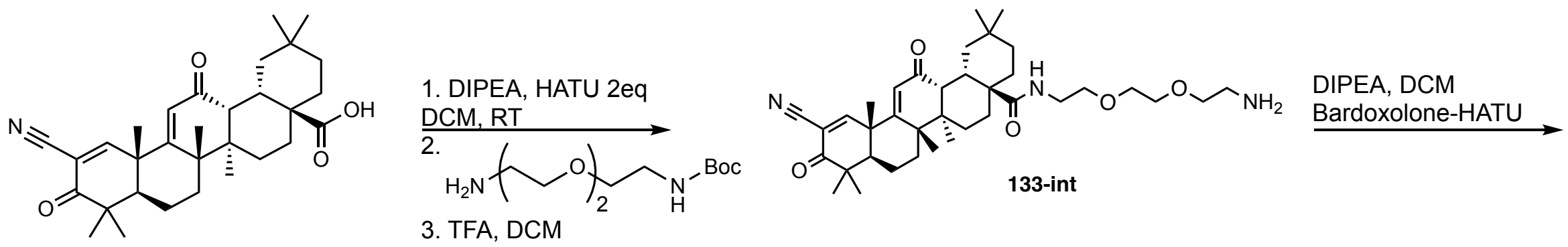

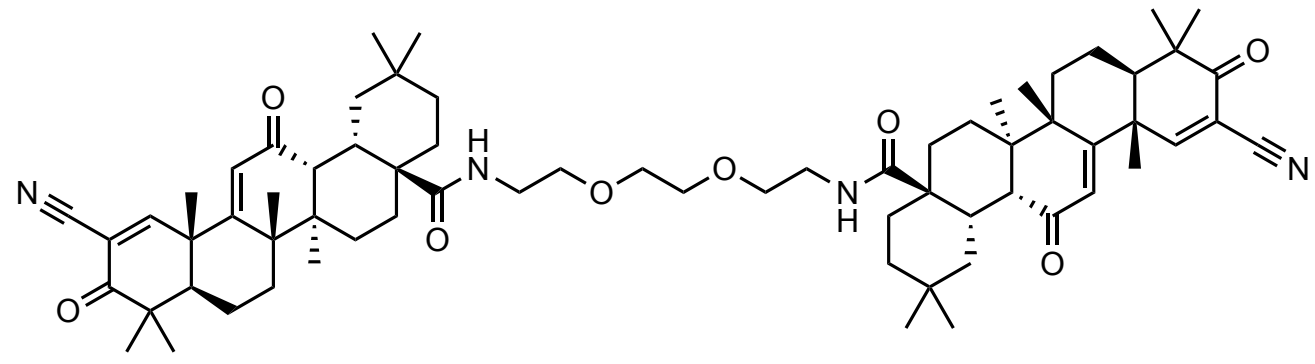

Synthetic procedure performed similar to the one described for EL147, using *tert*-butyl (2-(2-(2-aminoethoxy)ethoxy)ethyl)carbamate as the linker in the second step.

^{1}H NMR (500 MHz, $CDCl_3$) δ 8.27 (s, 1H), 6.64 (s, 1H), 6.17 (s, 1H), 3.60 – 3.48 (m, 5H), 3.03 (s, 1H), 2.91 (d, *J* = 10.3 Hz, 1H), 1.94 – 1.71 (m, 15H), 1.56 (s, 1H), 1.52 (s, 3H), 1.37 (s, 3H), 1.28 (s, 3H), 1.19 (s, 3H), 1.05 (s, 3H), 1.00 (s, 3H), 0.93 (s, 3H).

^{13}C NMR (126 MHz, $CDCl_3$) δ 199.41, 196.56, 177.44, 169.40, 166.12, 124.14, 114.57, 114.46, 70.38, 69.65, 49.72, 47.66, 46.71, 45.91, 45.02, 42.65, 42.15, 39.39, 36.20, 34.63, 33.98, 33.32, 32.11, 31.75, 30.69, 29.70, 27.71, 26.97, 26.54, 24.88, 23.09, 22.58, 21.81, 21.58, 18.25.

HR-MS (m/z): Calculated: 1094.71.; Found: 1095.7185 $[M+H]^+$, 1117.6956 $[M+Na]^+$

EL152 (9)- (4a*S*,4a'*S*,6a*R*,6b*S*,6a'*R*,6b'*S*,8a*R*,8a'*R*,12a*S*,12a'*S*,14a*R*,14b*S*,14a'*R*,14b'*S*)-*N*a,*N*a'-(3,6,9,12,15,18-hexaoxaicosane-1,20-diyl)bis(11-cyano-2,2,6a,6b,9,9,12a-heptamethyl-10,14-dioxo-1,3,4,5,6,6a,6b,7,8,8a,9,10,12a,14,14a,14b-hexadecahydropicene-4a(2*H*)-carboxamide)

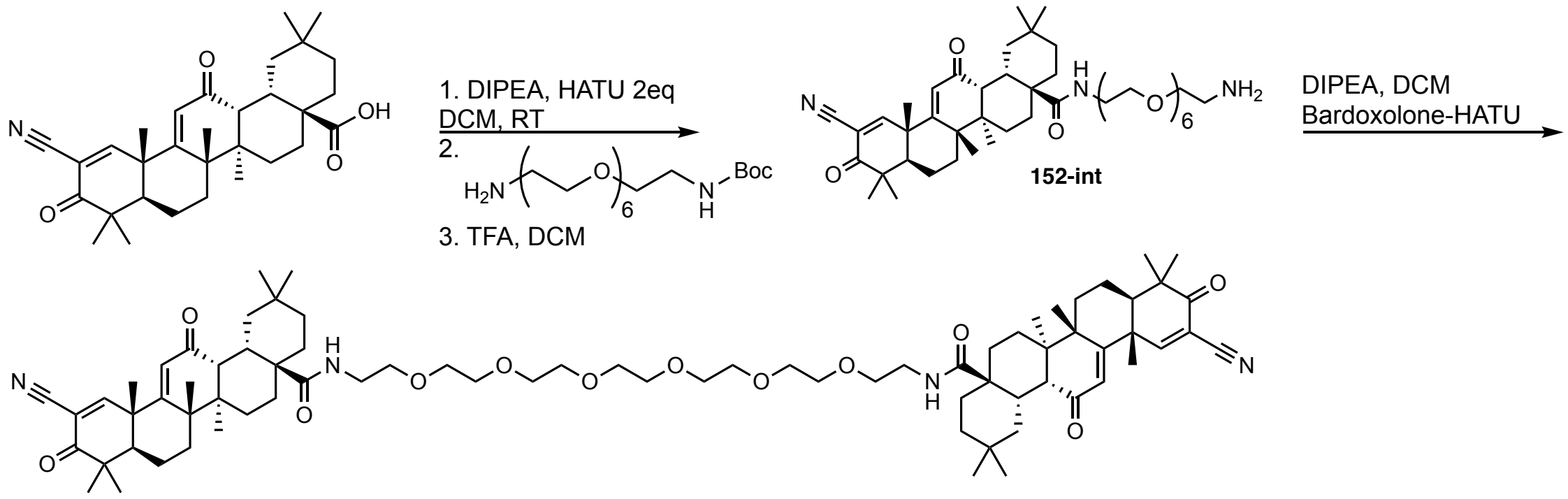

Synthetic procedure performed similar to the one described for EL147, using *tert-butyl (20-amino-3,6,9,12,15,18-hexaoxaicosyl)carbamate* as the linker in the second step.

HR-MS (m/z): Calculated: 1270.81.; Found: 1271.8190 $[M+H]^+$, 1293.8079 $[M+Na]^+$

EL165 (10)- (4a*S*,4a'*S*,6a*R*,6b*S*,6a'*R*,6b'*S*,8a*R*,8a'*R*,12a*S*,12a'*S*,14a*R*,14b*S*,14a'*R*,14b'*S*)-*N*a,*N*a'-(piperazine-1,4-diylbis(ethane-2,1-diyl))bis(11-cyano-2,2,6a,6b,9,9,12a-heptamethyl-10,14-dioxo-1,3,4,5,6,6a,6b,7,8,8a,9,10,12a,14,14a,14b-hexadecahydropicene-4a(2*H*)-carboxamide)

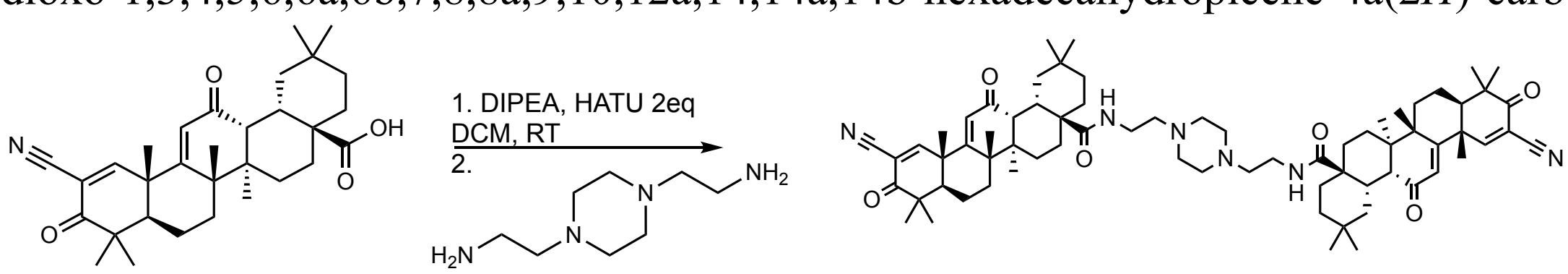

Synthetic procedure performed similar to the one described for EL229, using 2,2'-(piperazine-1,4-diyl)bis(ethan-1-amine) as the linker in the second step.

^{1}H NMR (500 MHz, $CDCl_3$) δ 8.08 (d, *J* = 10.8 Hz, 1H), 8.03 (s, 1H), 6.01 (s, 1H), 3.83 (s, 1H), 3.55 (d, *J* = 4.6 Hz, 4H), 3.15 (d, *J* = 16.1 Hz, 2H), 3.03 (d, *J* = 3.7 Hz, 1H), 2.96 (d, *J* = 12.8 Hz, 1H), 2.03 (t, *J* = 12.7 Hz, 2H), 1.70 (ddd, *J* = 54.5, 26.4, 15.0 Hz, 9H), 1.52 (s, 3H), 1.38 (dd, *J* = 10.7, 8.3 Hz, 2H), 1.33 (s, 3H), 1.28 (s, 3H), 1.19 (s, 3H), 1.04 (d, *J* = 6.6 Hz, 3H), 1.00 (s, 3H), 0.93 (s, 3H).

^{13}C NMR (126 MHz, $CDCl_3$) δ 199.31, 196.57, 178.45, 165.79, 161.95, 124.08, 114.57, 114.46, 57.93, 49.86, 49.47, 47.68, 46.64, 45.83, 45.02, 42.59, 42.02, 35.90, 34.59, 33.56, 33.26, 31.70, 31.62, 30.59, 29.71, 27.80, 26.96, 26.55, 24.77, 23.16, 23.06, 22.61, 21.69, 21.57, 18.22.

HR-MS (m/z): Calculated: 1658.52; Found: 1119.7657 $[M+H]^+$.

EL164 (11)- (4a*S*,4a'*S*,6a*R*,6b*S*,6a'*R*,6b'*S*,8a*R*,8a'*R*,12a*S*,12a'*S*,14a*R*,14b*S*,14a'*R*,14b'*S*)-*N*a,*N*a'-((ethane-1,2-diylbis(sulfanediyl))bis(ethane-2,1-diyl))bis(11-cyano-2,2,6a,6b,9,9,12a-heptamethyl-10,14-dioxo-1,3,4,5,6,6a,6b,7,8,8a,9,10,12a,14,14a,14b-hexadecahydropicene-4a(2*H*)-carboxamide)

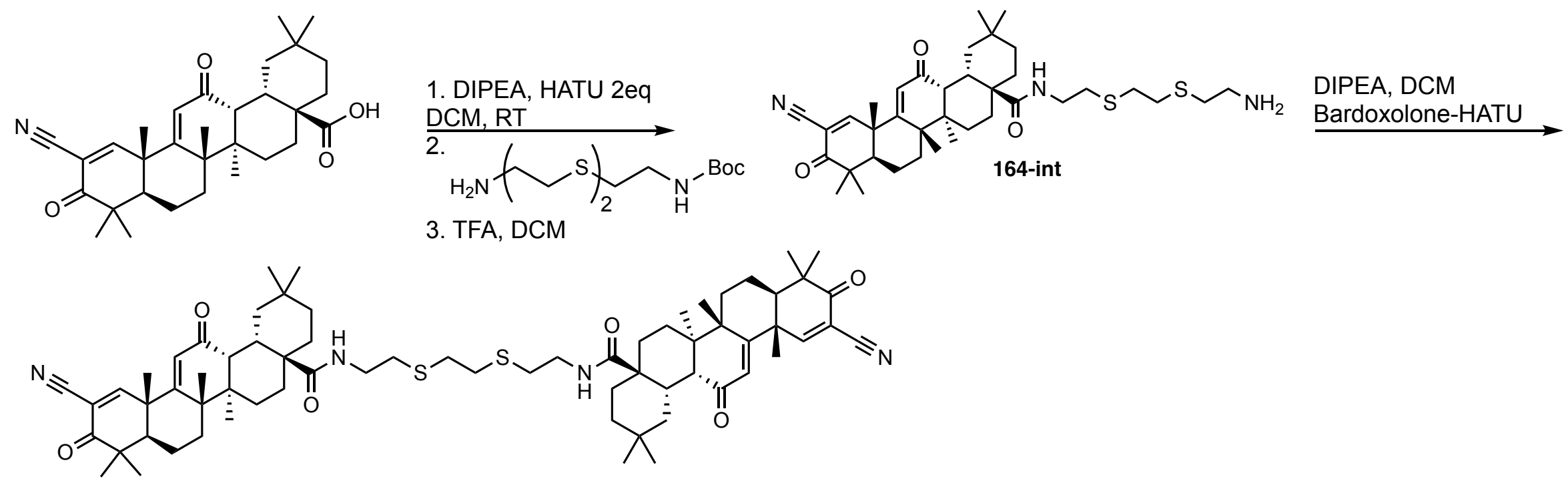

Synthetic procedure performed similar to the one described for EL147, using *tert-butyl (2-((2-((2-aminoethyl)thio)ethyl)thio)ethyl)carbamate* as the linker in the second step.

^{1}H NMR (500 MHz, DMSO) δ 8.65 (s, 1H), 7.84 (t, J = 5.2 Hz, 1H), 6.20 (s, 1H), 3.36 (dd, J = 12.3, 5.7 Hz, 1H), 3.30 (s, 1H), 3.21 – 3.13 (m, 1H), 3.05 (d, J = 4.4 Hz, 1H), 2.85 (d, J = 13.3 Hz, 1H), 2.71 (s, 2H), 2.66 – 2.57 (m, 2H), 1.94 – 1.76 (m, 3H), 1.61 (ddd, J = 21.3, 17.4, 8.2 Hz, 5H), 1.44 (d, J = 3.1 Hz, 1H), 1.43 (s, 3H), 1.25 (d, J = 6.0 Hz, 3H), 1.17 (s, 3H), 1.06 (s, 3H), 0.94 (s, 3H), 0.88 (d, J = 8.8 Hz, 3H), 0.86 (s, 3H).

^{13}C NMR (126 MHz, DMSO) δ 199.67, 197.79, 177.13, 169.45, 168.83, 123.86, 115.58, 113.27, 49.16, 46.68, 45.89, 45.87, 44.84, 43.02, 42.02, 40.59, 39.17, 35.81, 34.61, 33.71, 33.60, 31.66, 31.37, 31.22, 31.02, 30.69, 27.79, 26.58, 26.35, 24.84, 23.63, 22.36, 21.77, 21.61, 17.92.

HR-MS (m/z): Calculated: 1126.66; Found: 1127.6669 $[M+H]^{+}$, 1149.6544 $[M+Na]^{+}$

EL132 (12)- ((4a*S*,4a'*S*,6a*R*,6b*S*,6a'*R*,6b'*S*,8a*R*,8a'*R*,12a*S*,12a'*S*,14a*R*,14b*S*,14a'*R*,14b'*S*)-*N*a,*N*a'-(hexane-1,6-diyl)bis(11-cyano-2,2,6a,6b,9,9,12a-heptamethyl-10,14-dioxo-1,3,4,5,6,6a,6b,7,8,8a,9,10,12a,14,14a,14b-hexadecahydropicene-4a(2*H*)-carboxamide)

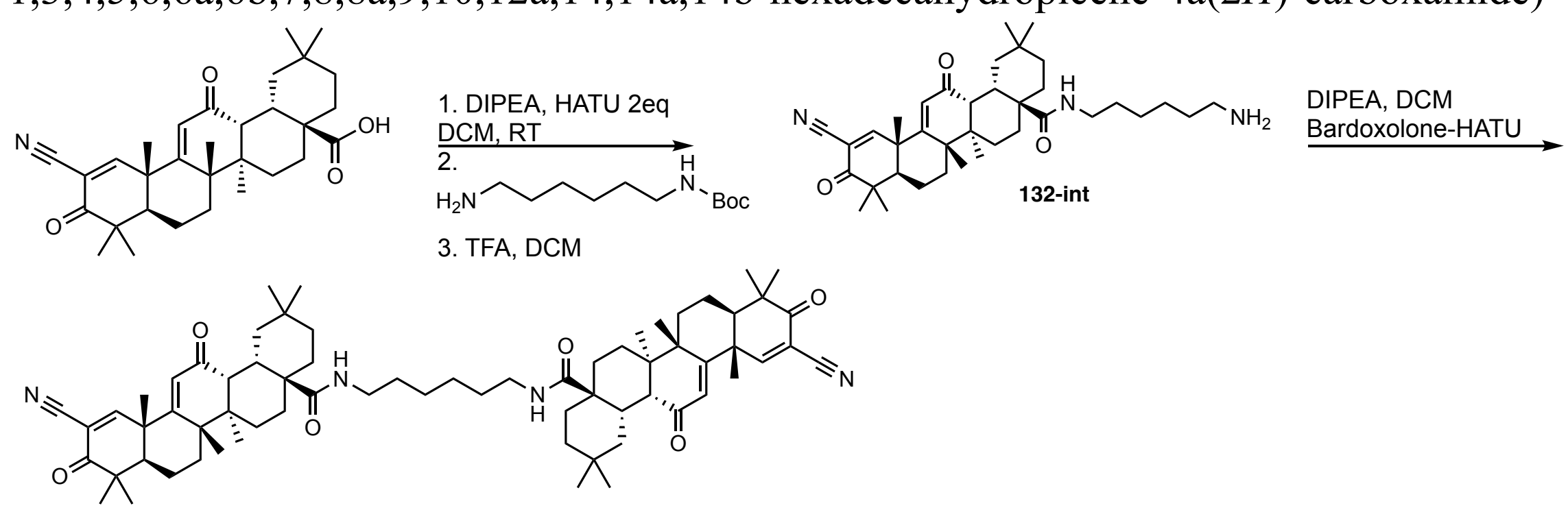

Synthetic procedure performed similar to the one described for EL147, using *tert-butyl (6-aminohexyl)carbamate* as the linker in the second step.

^{1}H NMR (500 MHz, $CDCl_3$) δ 8.07 (s, 1H), 5.99 (s, 1H), 5.97 (t, J = 5.9 Hz, 1H), 3.26 (qd, J = 13.3, 6.4 Hz, 2H), 3.05 (d, J = 4.4 Hz, 1H), 2.92 – 2.85 (m, 1H), 1.82 – 1.78 (m, 4H), 1.60 (s, 14H), 1.51 (s, 3H), 1.36 – 1.33 (m, J = 9.7 Hz, 4H), 1.28 (s, 3H), 1.19 (s, 3H), 1.04 (s, 3H), 1.02 (s, 3H), 0.93 (s, 3H).

^{13}C NMR (126 MHz, $CDCl_3$) δ 199.04, 196.52, 177.13, 168.91, 165.63, 123.97, 114.65, 114.37, 49.58, 47.71, 46.51, 45.92, 45.03, 42.56, 42.10, 39.16, 36.12, 34.61, 34.14, 33.27, 32.05, 31.69, 30.64, 29.73, 27.77, 27.00, 26.64, 26.14, 24.91, 23.13, 22.92, 21.78, 21.57, 18.25.

HR-MS (m/z): Calculated: 1062.72; Found: 1085.7102 $[M+H]^+$

EL163 (13)- (4a*S*,4a'*S*,6a*R*,6b*S*,6a'*R*,6b'*S*,8a*R*,8a'*R*,12a*S*,12a'*S*,14a*R*,14b*S*,14a'*R*,14b'*S*)-*N*a,*N*a'-(octane-1,8-diyl)bis(11-cyano-2,2,6a,6b,9,9,12a-heptamethyl-10,14-dioxo-1,3,4,5,6,6a,6b,7,8,8a,9,10,12a,14,14a,14b-hexadecahydropicene-4a(2*H*)-carboxamide)

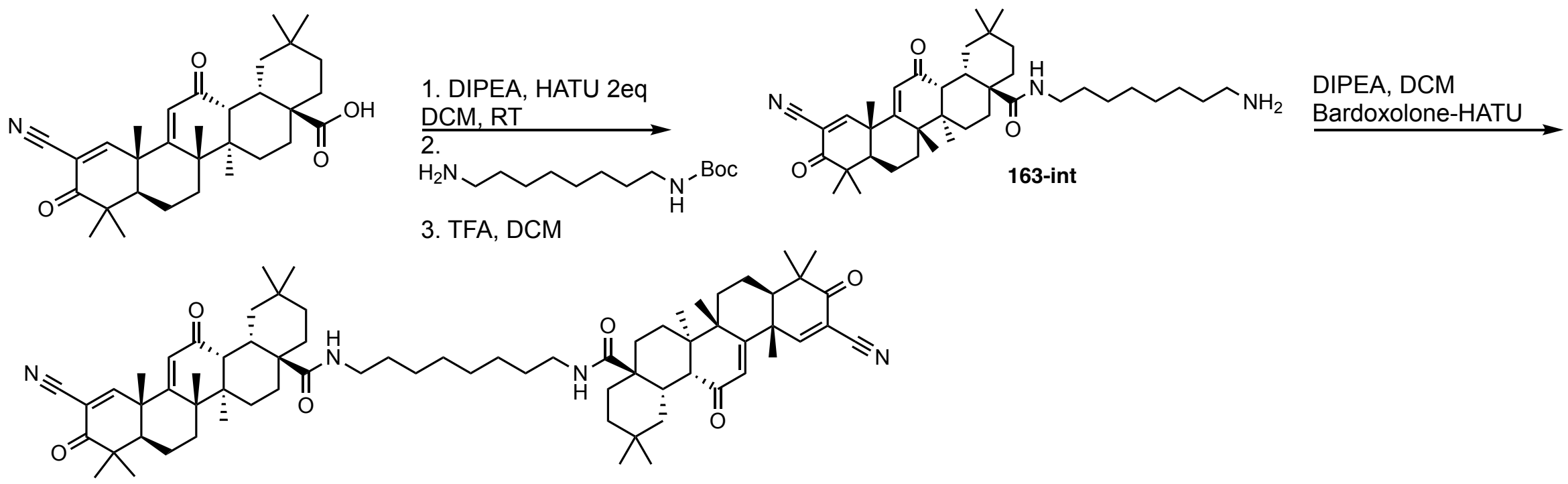

Synthetic procedure performed similar to the one described for EL147, using *tert-butyl (8-aminooctyl)carbamate* as the linker in the second step.

HR-MS (m/z): Calculated: 1090.75; Found: 1113.7347 $[M+Na]^+$

EL159 (14)- (4a*S*,4a'*S*,6a*R*,6b*S*,6a'*R*,6b'*S*,8a*R*,8a'*R*,12a*S*,12a'*S*,14a*R*,14b*S*,14a'*R*,14b'*S*)-*N*a,*N*a'-(cyclohexane-1,4-diylbis(methylene))bis(11-cyano-2,2,6a,6b,9,9,12a-heptamethyl-10,14-dioxo-1,3,4,5,6,6a,6b,7,8,8a,9,10,12a,14,14a,14b-hexadecahydropicene-4a(2*H*)-carboxamide)

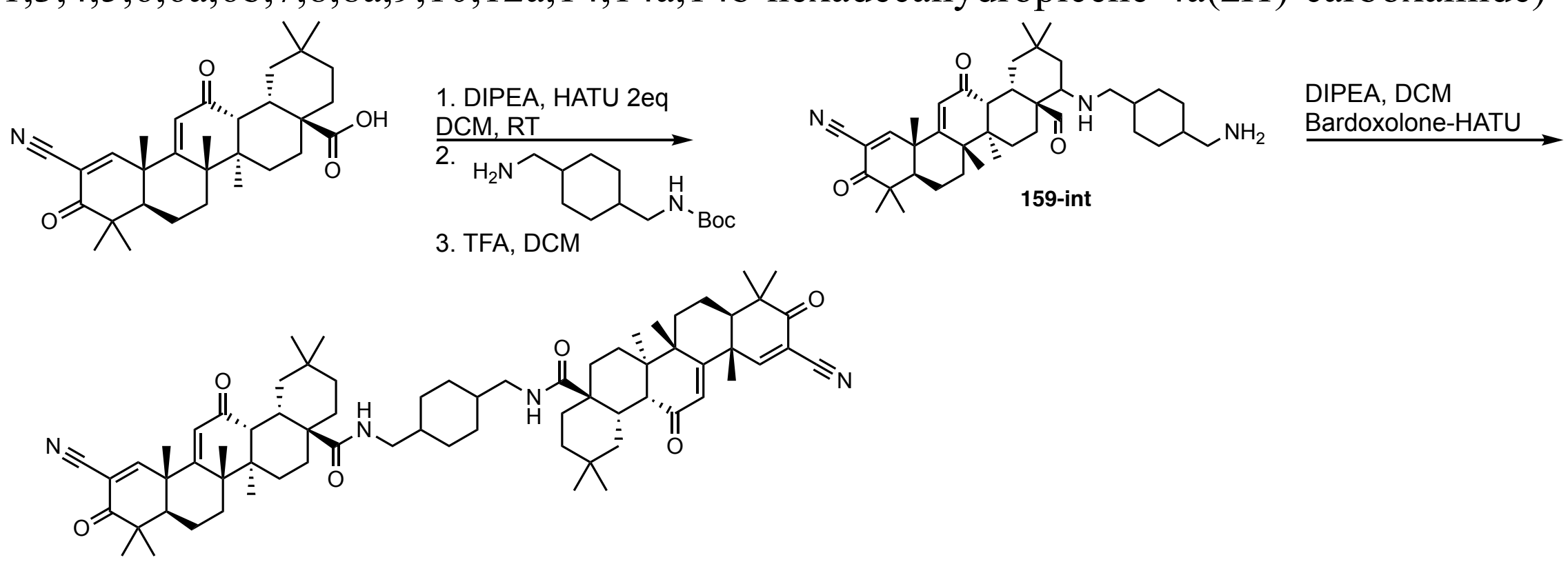

Synthetic procedure performed similar to the one described for EL147, using *tert*-butyl ((4-(aminomethyl)cyclohexyl)methyl)carbamate as the linker in the second step.

HR-MS (m/z): Calculated: 1088.73; Found: 1111.7249 $[M+Na]^+$.

EL174 (15)- (4a*S*,4a'*S*,6a*R*,6b*S*,6a'*R*,6b'*S*,8a*R*,8a'*R*,12a*S*,12a'*S*,14a*R*,14b*S*,14a'*R*,14b'*S*)-*N*a,*N*a'-(piperazine-1,4-diylbis(2-oxoethane-2,1-diyl))bis(11-cyano-2,2,6a,6b,9,9,12a-heptamethyl-

10,14-dioxo-1,3,4,5,6,6a,6b,7,8,8a,9,10,12a,14,14a,14b-hexadecahydropicene-4a(2*H*)-carboxamide)

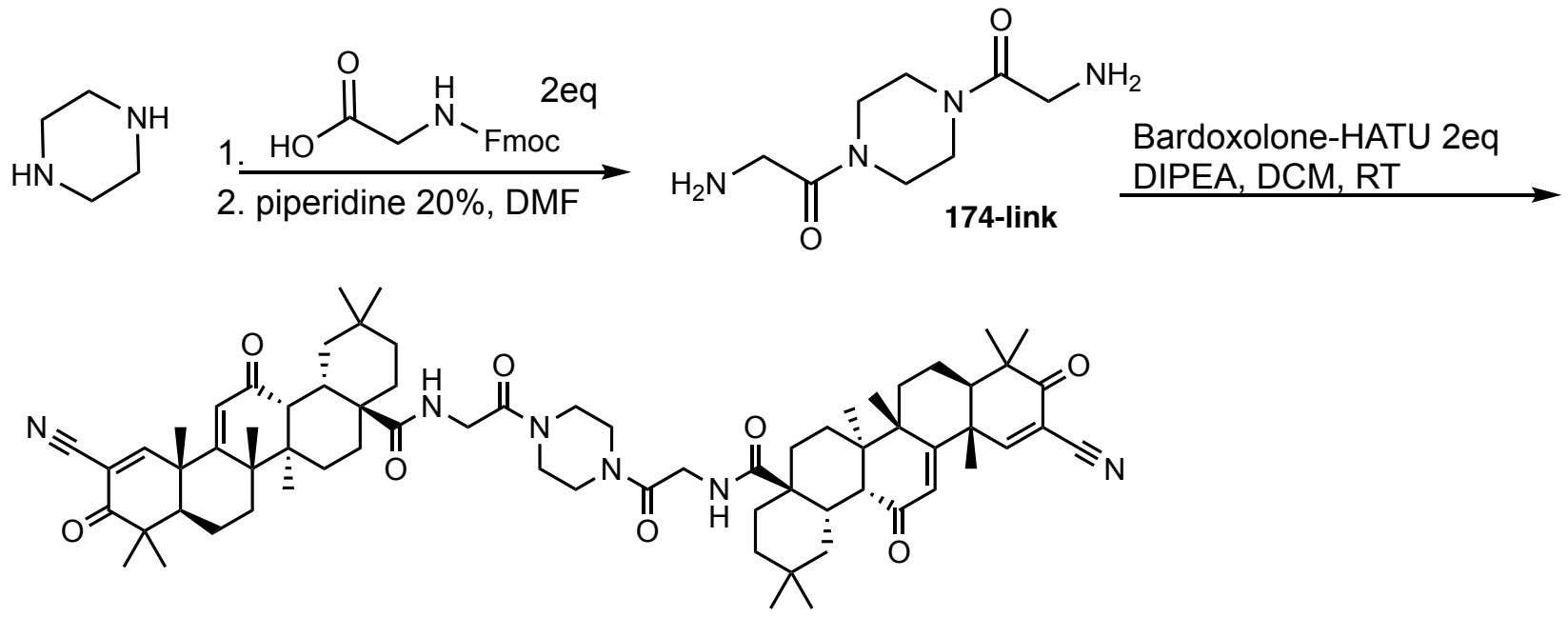

Fmoc-Gly-OH was reacted with 2.5 eq of EDC, 2eq of HOBt and 4 eq of TEA in DCM for 15 min on ice. Next, 30 mg of piperazine was added to the reaction which was allowed to warm to room temperature and kept overnight. The solvent was evaporated, and the crude product was dissolved in 20% piperidine in DMF, to allow Fmoc deprotection for 2 h in room temperature. The crude deprotected product (**174-link**) was directly used for the next reaction, where it was reacted with HATU-Bardoxolone as described in the procedure for compound EL229.

HR-MS (m/z): Calculated: 1046.71; Found: 1169.7006 $[M+Na]^+$

EL256 (16)- (4aS,4a'S,6aR,6bS,6a'R,6b'S,8aR,8a'R,12aS,12a'S,14aR,14bS,14a'R,14b'S)-Na,Na'-(25-(2-(3-(5,5-difluoro-7,9-dimethyl-5H-5l4,6l4-dipyrrolo[1,2-c:2',1'-f][1,3,2]diazaborinin-3-yl)propanamido)ethyl)-21,29-dioxo-3,6,9,12,15,18,32,35,38,41,44,47-dodecaoxa-22,25,28-triazanonatetracontane-1,49-diyl)bis(11-cyano-2,2,6a,6b,9,9,12a-heptamethyl-10,14-dioxo-1,3,4,5,6,6a,6b,7,8,8a,9,10,12a,14,14a,14b-hexadecahydropicene-4a(2H)-carboxamide)

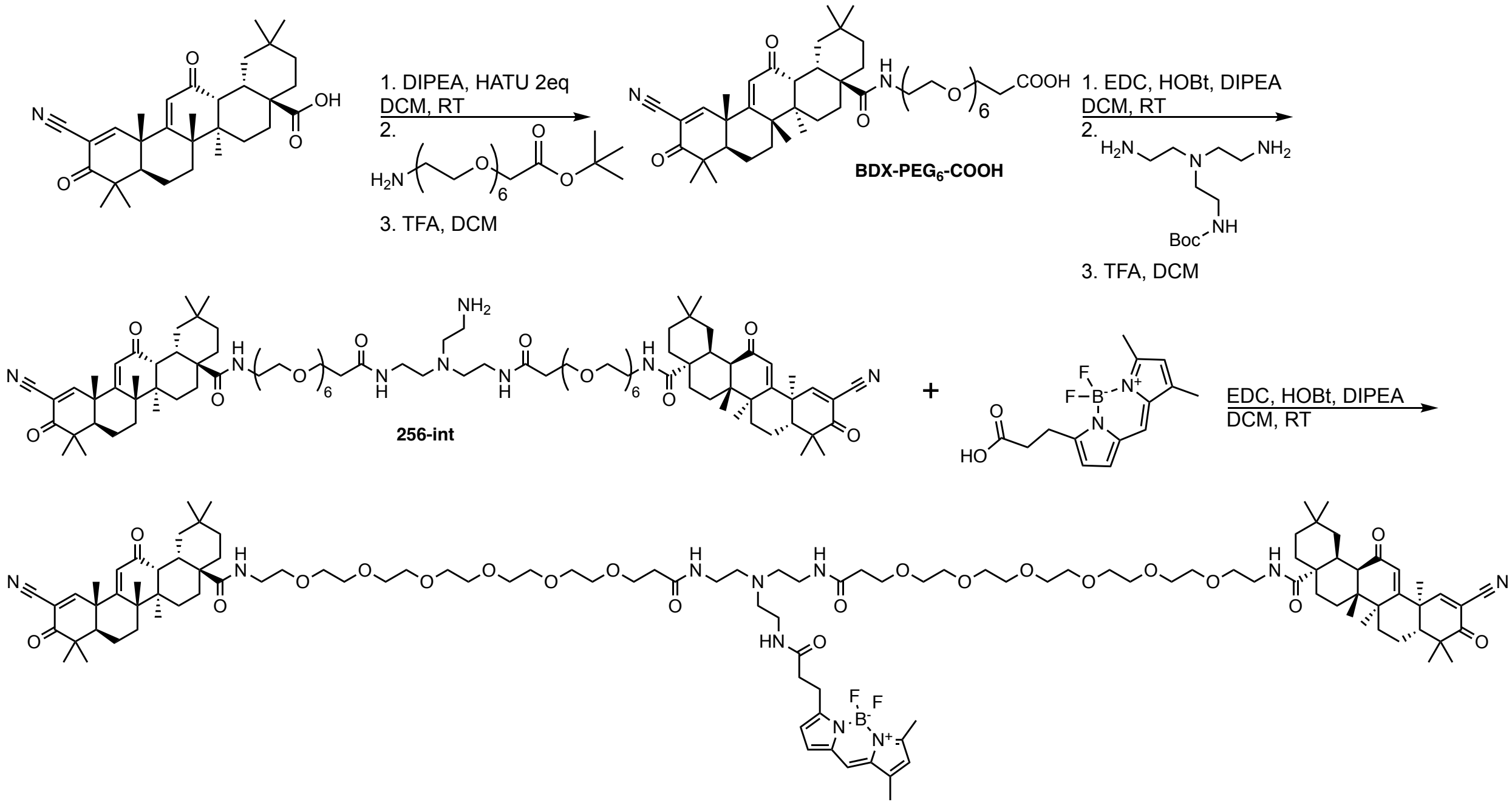

Bardoxolone-COOH was activated using HATU according to the synthetic procedure described for compound EL229, and it was reacted with *tert*-butyl 1-amino-3,6,9,12,15,18-

hexaoxahenicosan-21-oate in DCM overnight. TFA was added to the reaction to obtain a final solvent ratio of approximately 1:2 (TFA:DCM) and the reaction was left overnight at room temperature. The deprotected intermediate (**BDX-PEG$_6$-COOH**) was then evaporated and purified by HPLC, lyophilized, and then used directly in the following reaction. The purified deprotected acid was activated using 2eq EDC and HOBt, as well as 3eq DIPEA in DCM. 0.5eq of *tert*-butyl (2-(bis(2-aminoethyl)amino)ethyl)carbamate was then added and let react for 3-4 hours. TFA was added to the reaction to obtain a final solvent ratio of approximately 1:2 (TFA:DCM) for overnight at room temperature. The deprotected amine (**256-int**) was then purified by HPLC and lyophilized, to be used directly in the last step. An acid-BODIPY derivative (3-(5,5-difluoro-7,9-dimethyl-5H-5l4,6l4-dipyrrolo[1,2-c:2',1'-f][1,3,2]diazaborinin-3-yl)propanoic acid) was activated with 2eq EDC, HOBt, and 3eq DIPEA in DCM in a covered vial to protect from light. After 30 minutes in RT, the amine intermediate was added, the reaction was left in room temperature for 4 hours, and the final product was purified by HPLC.

^{1}H NMR (500 MHz, DMSO-d6) δ 8.64 (s, 1H), 7.90 – 7.71 (m, 3H), 7.68 (s, 1H), 7.09 (d, *J* = 4.1 Hz, 1H), 6.55 (s, 1H), 6.36 (d, *J* = 4.0 Hz, 1H), 6.30 (s, 1H), 6.19 (s, 1H), 5.66 (s, 1H), 3.58 (t, *J* = 6.4 Hz, 2H), 3.54 – 3.48 (m, 21H), 3.47 – 3.45 (m, 5H), 3.43 – 3.40 (m, 3H), 3.16 – 3.06 (m, 7H), 3.04 (d, *J* = 4.7 Hz, 1H), 3.01 (d, *J* = 4.6 Hz, 1H), 2.90 – 2.80 (m, 2H), 2.49 – 2.45 (m, 5H), 2.32 (t, *J* = 6.5 Hz, 2H), 2.26 (s, 2H), 1.93 – 1.82 (m, 2H), 1.69 – 1.64 (m, 2H), 1.62 (d, *J* = 11.2 Hz, 2H), 1.55 (dd, *J* = 13.9, 4.7 Hz, 2H), 1.42 (s, 3H), 1.24 (s, 3H), 1.17 (s, 3H), 1.06 (s, 3H), 0.93 (s, 3H), 0.88 (s, 3H), 0.85 (s, 3H).

^{13}C NMR (126 MHz, DMSO) δ 199.88, 199.87, 177.13, 170.55, 170.53, 125.79, 120.68, 117.04, 70.35, 70.33, 70.31, 70.28, 70.24, 70.21, 70.16, 70.11, 69.98, 69.95, 69.47, 67.33, 67.29, 53.86, 49.14, 48.97, 45.94, 45.79, 45.50, 45.42, 44.83, 44.52, 43.01, 42.25, 41.98, 41.69, 41.00, 39.23, 37.42, 36.58, 35.80, 34.66, 33.73, 33.50, 31.23, 30.72, 30.70, 30.66, 28.11, 27.86, 26.57, 26.32, 24.75, 24.70, 24.49, 23.70, 23.61, 23.55, 23.26, 22.32, 22.13, 21.85, 21.76, 21.68, 21.65, 21.55, 11.67, 11.45.

HR-MS (m/z): Calculated: 2038.2447; Found: 2038.2531 [M], 2039.2559 $[M+H]^+$, 2060.2356 $[M+Na]^+$

OS-47B (17)- 2,2'-(((6,25-bis(2-((2-methylbenzo[*d*]thiazol-6-yl)amino)-2-oxoethyl)-4,7,24,27-tetraoxo-11,14,17,20-tetraoxa-5,8,23,26-tetraazatriacontane-1,30-diyl)bis(4,1-phenylene))bis(methylene))dimalonic acid

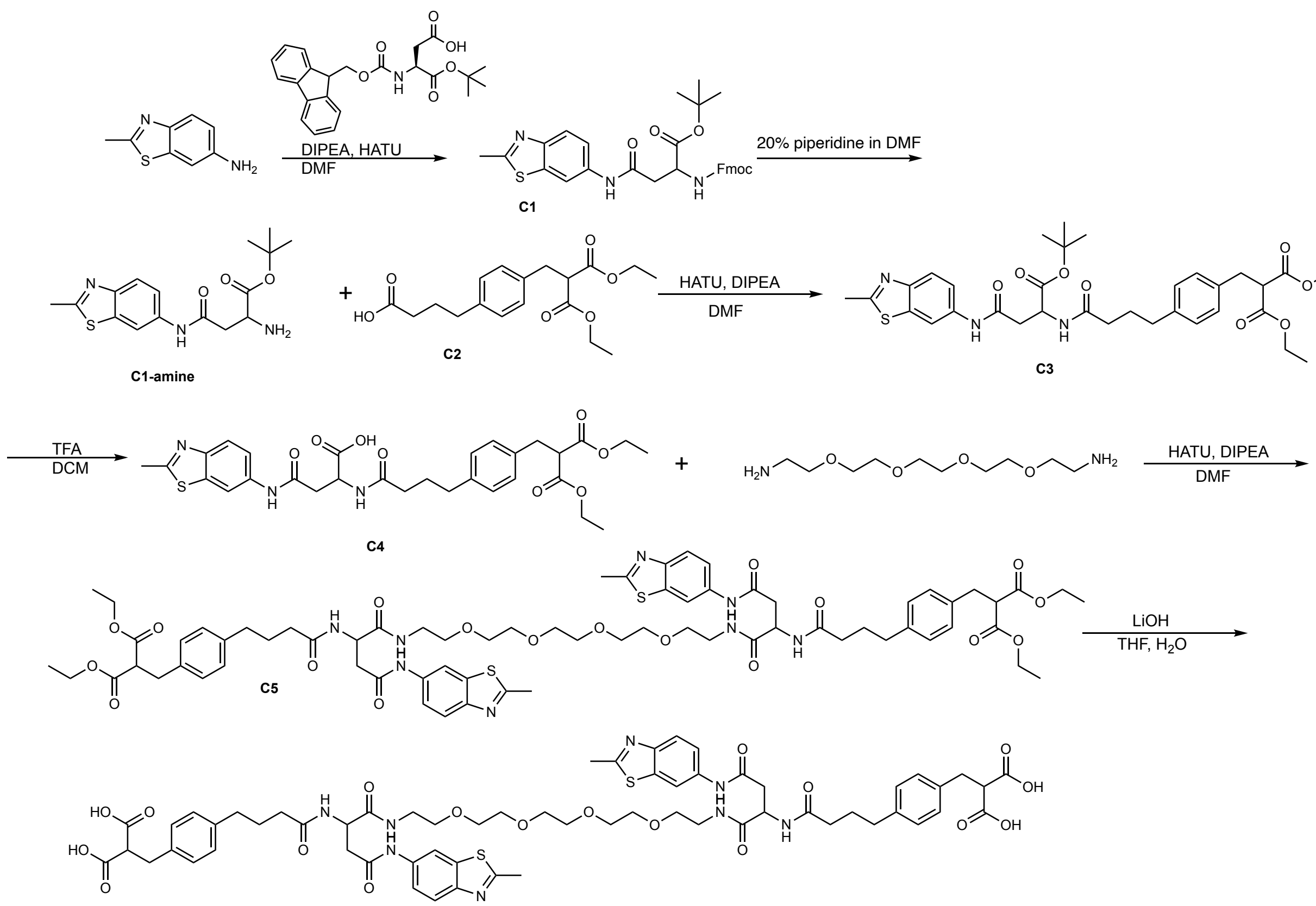

2-methylbenzo[d]thiazol-6-amine was coupled to Fmoc-L-aspartic acid-α-tert-butylester in the presence of DIPEA and HATU, in DMF for overnight at room temperature. The mixture was evaporated to dryness and the product was extracted using EtOAc, followed by sequential washes with water and brine. Finally, the organic phase was dried over Na_2SO_4 and concentrated under reduced pressure, and the crude was purified using flash chromatography (DCM:MeOH). Next, the Fmoc protecting group was removed using 20% piperidine in DMF for 1 h at room temperature, and the product was purified using flash chromatography. The resulting amine was then reacted with compound **C2** which was synthesized according to the previously reported procedure[1], using DIPEA and HATU, in DMF for overnight at room temperature. The solvent was evaporated to dryness, and the crude **C3** was redissolved in DCM and washed with water, 0.5 M HCl, and brine. The organic phase was dried over Na_2SO_4, evaporated under reduced pressure, and purified using flash chromatography. Deprotection of Boc group was performed in 1:1 TFA/DCM to afford compound **C4**. The free acid was then coupled on both ends of the diamine linker 3,6,9,12-tetraoxatetradecane-1,14-diamine in a single step using DIPEA and HATU, in DMF for overnight at room temperature. Resulting compound **C5** was purified by HPLC, then reacted with 1 M LiOH in a 1:1 THF/H2O solution to afford the final compound, which was purified by HPLC.

^{1}H NMR (500 MHz, DMSO) δ 12.72 (s, 2H), 10.11 (s, 1H), 8.39 (d, J = 1.8 Hz, 1H), 8.08 (d, J = 8.0 Hz, 1H), 7.83 (t, J = 5.6 Hz, 1H), 7.80 (d, J = 8.8 Hz, 1H), 7.47 (dd, J = 8.8, 1.9 Hz, 1H), 7.03 (dd, J = 25.5, 8.0 Hz, 4H), 4.69 (dd, J = 14.0, 7.9 Hz, 1H), 3.52 (t, J = 7.8 Hz, 2H), 3.46 (d, J = 5.2 Hz, 6H), 3.38 (t, J = 6.1 Hz, 2H), 3.20 (qd, J = 13.5, 7.0 Hz, 2H), 2.97 (d, J = 7.7 Hz, 2H),

2.79 (dt, J = 10.0, 5.1 Hz, 1H), 2.75 (s, 3H), 2.62 (dd, J = 15.2, 8.1 Hz, 1H), 2.49 – 2.43 (m, 2H), 2.12 (t, J = 7.3 Hz, 2H), 1.73 (dt, J = 13.9, 7.0 Hz, 2H), 1.29 – 1.23 (m, 1H).

^{13}C NMR (126 MHz, DMSO) δ 172.42, 171.44, 170.71, 168.90, 165.84, 149.37, 140.20, 136.66, 136.23, 136.20, 129.00, 128.64, 122.27, 118.69, 111.69, 70.20, 70.14, 70.03, 69.31, 53.82, 50.12, 39.17, 39.14, 35.24, 34.58, 34.26, 27.41, 20.08.

HR-MS (m/z): Calculated: 1281.4460; Found: 1281.4456 $[M+H]^+$

EL244 (18)- 1-(4-((2-methyl-5-(8-methylimidazo[1,2-a]pyridin-2-yl)phenyl)sulfonyl)piperazin-1-yl)-2-(2-(4-((2-methyl-5-(8-methylimidazo[1,2-a]pyridin-2-yl)phenyl)sulfonyl)piperazin-1-yl)-2-oxoethoxy)ethan-1-one

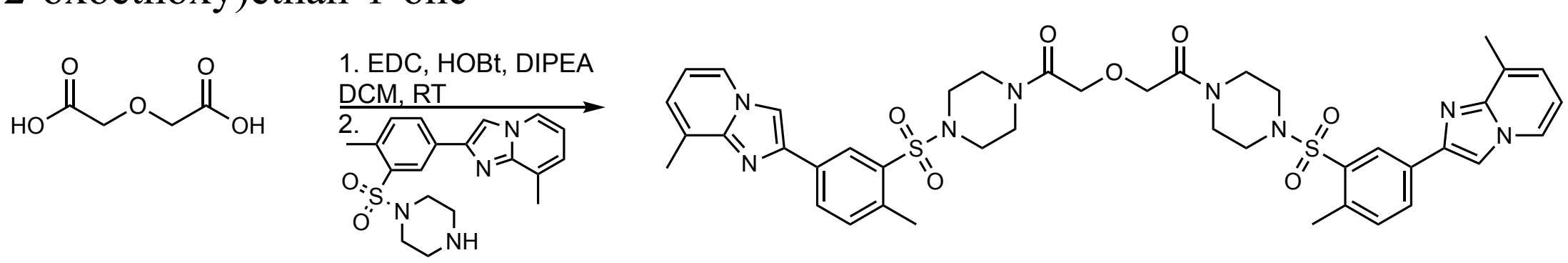

2,2'-oxydiacetic acid was activated using 4 eq EDC and 4eq HOBt, along with 6 eq DIPEA in DCM for 15 min at room temperature. Next, amine ligand 8-methyl-2-(4-methyl-3-(piperazin-1-ylsulfonyl)phenyl)imidazo[1,2-a]pyridine was added to the reaction mixture and allowed to react overnight in room temperature. The crude reaction was evaporated, dissolved in 30% ACN in H2O, and purified by HPLC.

^{1}H NMR (500 MHz, DMSO) δ 8.77 (s, 2H), 8.60 (s, 2H), 8.43 (d, J = 1.2 Hz, 2H), 8.26 (t, J = 6.8 Hz, 2H), 8.23 (d, J = 7.2 Hz, 2H), 7.63 (dd, J = 13.6, 6.3 Hz, 2H), 7.11 – 7.01 (m, 2H), 4.16 (s, 4H), 3.13 – 3.06 (m, 16H), 2.64 (s, 6H), 2.62 (s, 6H).

^{13}C NMR (126 MHz, DMSO) δ 167.55, 135.84, 134.40, 134.11, 131.19, 128.21, 127.19, 125.27, 121.89, 114.34, 69.38, 45.94, 45.41, 44.28, 41.06, 20.67, 20.64, 17.10, 16.50, 8.97.

HR-MS (m/z): Calculated: 838.29 ; Found: 839.2986 $[M+H]^+$, 861.2801 $[M+Na]^+$

EL245 (19)- 1-(4-((2-methyl-5-(8-methylimidazo[1,2-a]pyridin-2-yl)phenyl)sulfonyl)piperazin-1-yl)-2-(2-(2-(4-((2-methyl-5-(8-methylimidazo[1,2-a]pyridin-2-yl)phenyl)sulfonyl)piperazin-1-yl)-2-oxoethoxy)ethoxy)ethan-1-one

Synthetic procedure performed similar to the one described for EL244, using 2,2'-(ethane-1,2-diylbis(oxy))diacetic acid as the linker.

^{1}H NMR (500 MHz, DMSO) δ 8.51 (d, J = 15.0 Hz, 2H), 8.40 (d, J = 1.4 Hz, 2H), 8.37 (d, J = 6.7 Hz, 2H), 8.12 (dd, J = 7.9, 1.5 Hz, 2H), 7.51 (d, J = 8.0 Hz, 2H), 7.08 (d, J = 6.7 Hz, 2H), 6.82 (t, J = 6.8 Hz, 2H), 4.08 (s, 4H), 3.46 (d, J = 16.5 Hz, 12H), 3.08 (d, J = 20.4 Hz, 8H), 2.58 (s, 6H), 2.53 (d, J = 6.9 Hz, 6H).
^{13}C NMR (126 MHz, DMSO) δ 167.80, 145.92, 142.45, 136.83, 135.77, 134.05, 133.05, 130.41, 126.92, 126.73, 125.20, 124.27, 112.98, 110.76, 69.94, 69.58, 45.62, 45.40, 44.37, 41.04, 20.55, 17.17.

HR-MS (m/z): Calculated: 882.32 ; Found: 883.3245 $[M+H]^+$, 905.3062 $[M+Na]^+$

EL246 (20)- 1-(4-((2-methyl-5-(8-methylimidazo[1,2-a]pyridin-2-yl)phenyl)sulfonyl)piperazin-1-yl)-2-(2-(2-(2-(4-((2-methyl-5-(8-methylimidazo[1,2-a]pyridin-2-yl)phenyl)sulfonyl)piperazin-1-yl)-2-oxoethoxy)ethoxy)ethoxy)ethan-1-one

Synthetic procedure performed similarly to the one described for EL244, using 2,2'-((oxybis(ethane-2,1-diyl))bis(oxy))diacetic acid as the linker.

^{1}H NMR (500 MHz, DMSO) δ 8.49 (d, J = 3.4 Hz, 2H), 8.39 (d, J = 1.5 Hz, 2H), 8.37 (d, J = 6.7 Hz, 2H), 8.12 (dd, J = 7.8, 1.6 Hz, 2H), 7.51 (d, J = 8.0 Hz, 2H), 7.07 (d, J = 6.9 Hz, 2H), 6.81 (d, J = 6.7 Hz, 2H), 4.09 (s, 4H), 3.53 – 3.47 (m, 8H), 3.45 – 3.41 (m, 4H), 3.09 (m, 8H), 2.58 (s, 6H), 2.53 (s, 6H).
^{13}C NMR (126 MHz, DMSO) δ 167.85, 145.91, 142.45, 136.81, 135.73, 134.04, 133.04, 130.40, 126.88, 126.72, 125.19, 124.25, 112.97, 110.75, 70.17, 69.85, 69.72, 20.56, 17.16.

HR-MS (m/z): Calculated: 926.35 ; Found: 949.3311 $[M+Na]^+$

EL241 (21)- 1,34-bis(4-((2-methyl-5-(8-methylimidazo[1,2-a]pyridin-2-yl)phenyl)sulfonyl)piperazin-1-yl)-4,7,10,13,16,19,22,25,28,31-decaoxatetratriacontane-1,34-dione

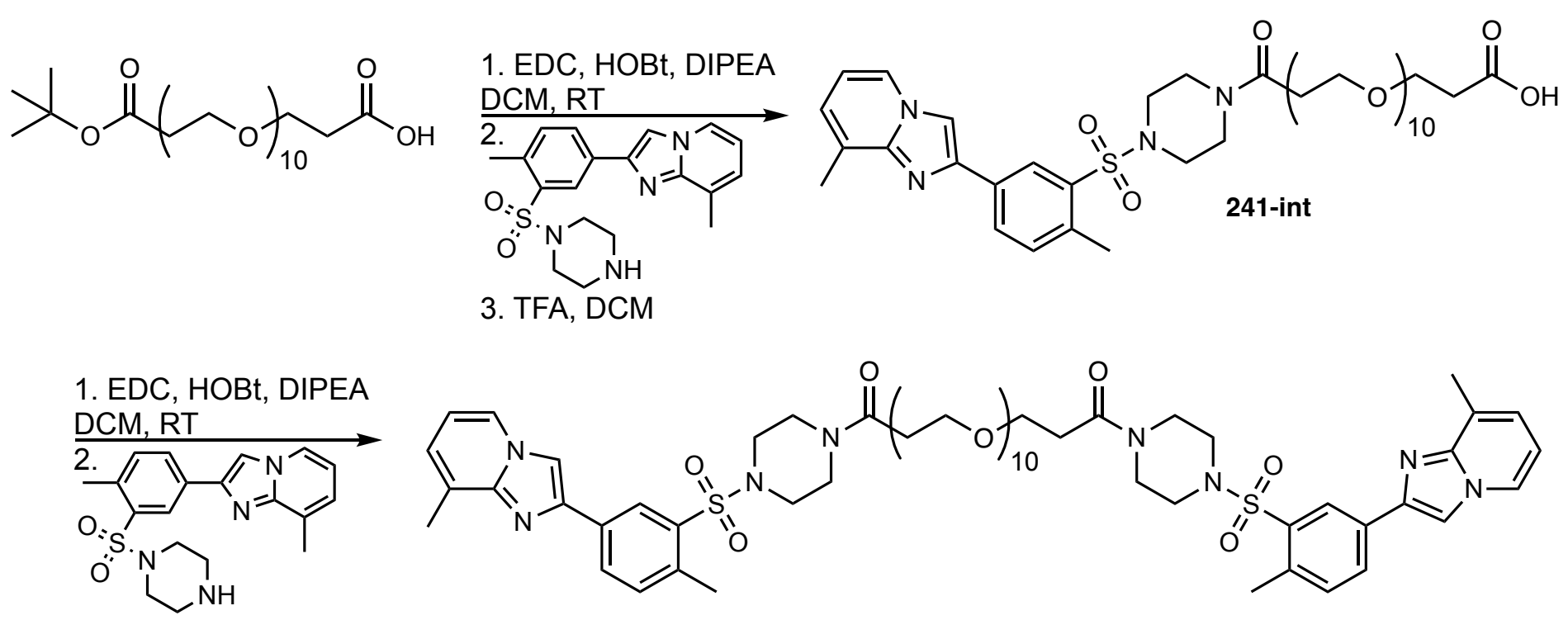

2,2-dimethyl-4-oxo-3,7,10,13,16,19,22,25,28,31,34-undecaoxaheptatriacontan-37-oic acid was activated using 2 eq EDC and 2eq HOBt, along with 6 eq DIPEA in DCM for 15 min in room temperature. Next, amine ligand (synthesis described previously[2]) 8-methyl-2-(4-methyl-3-(piperazin-1-ylsulfonyl)phenyl)imidazo[1,2-a]pyridine was added to the reaction mixture and allowed to react for 2 h in room temperature.
TFA was then added to double the solvent volume and left to deprotect overnight. The deprotected intermediate (**241-int**) was then evaporated and purified by HPLC, lyophilized, and was then used directly in the following reaction. The purified acid was reacted with EDC, HOBt, and DIPEA, as described in the first step, with amine ligand. After 2 h, the crude reaction was evaporated, dissolved in 40% ACN in H2O, and purified by HPLC.

^{1}H NMR (500 MHz, DMSO) δ 8.71 (s, 2H), 8.55 (d, J = 6.5 Hz, 2H), 8.42 (d, J = 1.3 Hz, 2H), 8.16 (dt, J = 5.4, 2.7 Hz, 2H), 7.62 (d, J = 8.0 Hz, 2H), 7.42 (s, 2H), 7.13 (s, 2H), 3.55 (d, J = 6.8 Hz, 8H), 3.46 (m, 40H), 3.10 (d, J = 29.7 Hz, 8H), 2.62 (s, 6H), 2.60 (s, 6H), 2.55 (t, J = 6.6 Hz, 4H).

^{13}C NMR (126 MHz, DMSO) δ 169.50, 163.50, 158.71, 158.44, 138.37, 136.18, 134.34, 130.99, 127.48, 126.11, 125.14, 117.92, 115.57, 115.29, 111.91, 70.21, 70.18, 70.14, 70.08, 67.16, 45.73, 45.46, 45.09, 40.95, 33.16, 20.63, 16.95.

HR-MS (m/z): Calculated: 1262.56 ; Found: 1263.5619 $[M+H]^+$, 1285.5447 $[M+Na]^+$

Amine ligand (synthesized previously as described above) characterization:

^{1}H NMR (500 MHz, DMSO) δ 8.51 (s, 1H), 8.39 (d, *J* = 1.6 Hz, 1H), 8.38 (s, 1H), 8.14 (dd, *J* = 7.8, 1.5 Hz, 1H), 7.54 (d, *J* = 8.0 Hz, 1H), 7.09 (d, *J* = 6.8 Hz, 1H), 6.83 (t, *J* = 6.8 Hz, 1H), 3.06 – 2.99 (m, 4H), 2.82 – 2.76 (m, 4H), 2.60 (s, 3H), 2.55 (s, 3H).

^{13}C NMR (126 MHz, DMSO) δ 145.93, 142.51, 136.82, 135.75, 134.00, 132.99, 130.29, 126.96, 126.73, 125.20, 124.26, 112.98, 110.74, 45.92, 45.07, 20.64, 17.19.
HR-MS (m/z): Calculated: 370.15 ; Found: 371.1538 $[M+H]^+$

EL230 (22)- 3-(((4-chloro-3-((3-((1-((4a*S*,6a*R*,6b*S*,8a*R*,12a*S*,14a*R*,14b*S*)-11-cyano-2,2,6a,6b,9,9,12a-heptamethyl-10,14-dioxo-1,3,4,5,6,6a,6b,7,8,8a,9,10,12a,14,14a,14b-hexadecahydropicen-4a(2*H*)-yl)-1,42-dioxo-5,8,11,14,17,20,23,26,29,32,35,38-dodecaoxa-2,41-diazatritetracontan-43-yl)oxy)-8-methoxy-1-methyl-2-oxo-1,2-dihydroquinolin-6-yl)amino)phenyl)amino)methyl)benzenesulfonyl fluoride

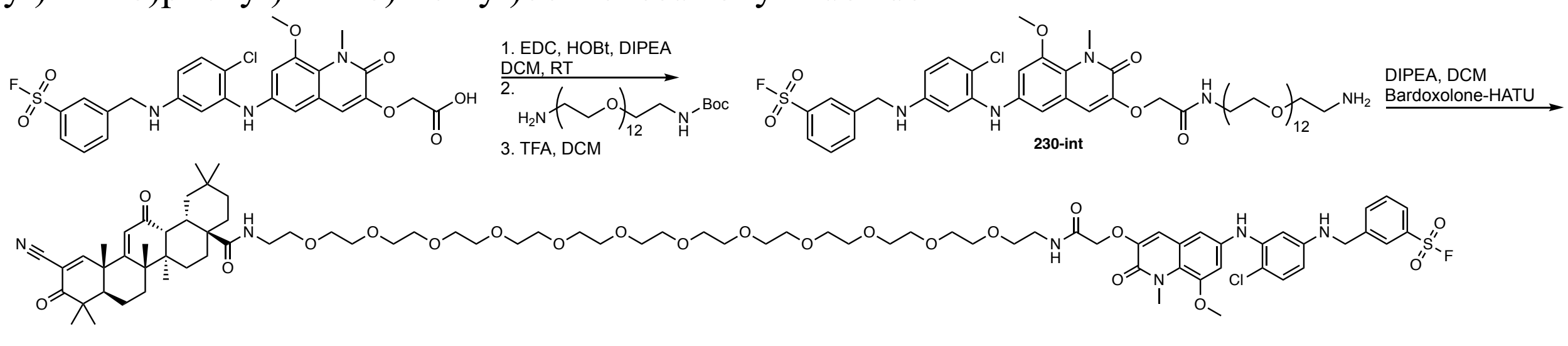

Synthetic procedure performed similar to the one described for EL228, using *tert-butyl (38-amino-3,6,9,12,15,18,21,24,27,30,33,36-dodecaoxaoctatriacontyl)carbamate* as the linker in the second step. The intermediate deprotected amine (**230-int**) was purified by HPLC, lyophilized and was then used directly in the following reaction. Bardoxlone-HATU, prepared as described for compound EL229 was added to the reaction along with 3 eq of DIPEA in DCM. After 2 h in room temperature, the crude reaction was evaporated, dissolved in 50% ACN in H_2O, and purified by HPLC.

^{1}H NMR (500 MHz, DMSO) δ 8.65 (s, 1H), 8.01 (d, *J* = 7.7 Hz, 1H), 7.98 (d, *J* = 6.8 Hz, 1H), 7.86 (d, *J* = 7.7 Hz, 1H), 7.73 (dd, *J* = 14.8, 6.9 Hz, 2H), 7.41 (s, 1H), 7.09 (d, *J* = 8.7 Hz, 1H), 7.05 (s, 1H), 6.87 (d, *J* = 2.0 Hz, 1H), 6.75 (d, *J* = 1.8 Hz, 1H), 6.55 (d, *J* = 5.9 Hz, 1H), 6.54 – 6.49 (m, 2H), 6.23 – 6.15 (m, 2H), 4.54 (s, 2H), 4.38 (d, *J* = 5.8 Hz, 2H), 3.83 (s, 3H), 3.79 (s, 3H), 3.49 (dd, *J* = 5.7, 3.0 Hz, 48H), 3.47 – 3.39 (m, 6H), 3.30 (t, *J* = 5.6 Hz, 4H), 1.92 – 1.75

(m, 3H), 1.65 (dd, J = 27.7, 15.1 Hz, 5H), 1.55 (dd, J = 13.6, 3.9 Hz, 2H), 1.43 (s, 3H), 1.24 (s, 3H), 1.17 (s, 3H), 1.06 (s, 3H), 0.93 (s, 3H), 0.89 (s, 3H), 0.85 (s, 3H).

^{13}C NMR (126 MHz, DMSO) δ 199.73, 197.79, 177.10, 168.82, 167.62, 158.06, 148.94, 148.40, 146.87, 143.88, 141.00, 139.11, 135.58, 132.16, 130.93, 130.52, 127.14, 126.83, 123.83, 122.77, 121.37, 114.43, 113.27, 110.71, 108.58, 106.68, 105.29, 102.77, 70.23, 70.16, 70.04, 69.99, 69.46, 69.30, 68.25, 56.94, 49.13, 46.68, 46.16, 45.86, 45.80, 44.83, 43.01, 41.98, 39.23, 38.78, 35.80, 35.45, 34.62, 33.72, 33.46, 31.22, 30.69, 27.68, 26.57, 26.32, 24.70, 23.60, 22.23, 21.76, 21.59, 17.94.

HR-MS (m/z): Calculated: 1618.76; Found: 1641.7552 $[M+Na]^+$

w8001-PEG13-NH2 (23)- 3-(((3-((3-((44-amino-2-oxo-6,9,12,15,18,21,24,27,30,33,36,39,42-tridecaoxa-3-azatetratetracontyl)oxy)-8-methoxy-1-methyl-2-oxo-1,2-dihydroquinolin-6-yl)amino)-4-chlorophenyl)amino)methyl)benzenesulfonyl fluoride

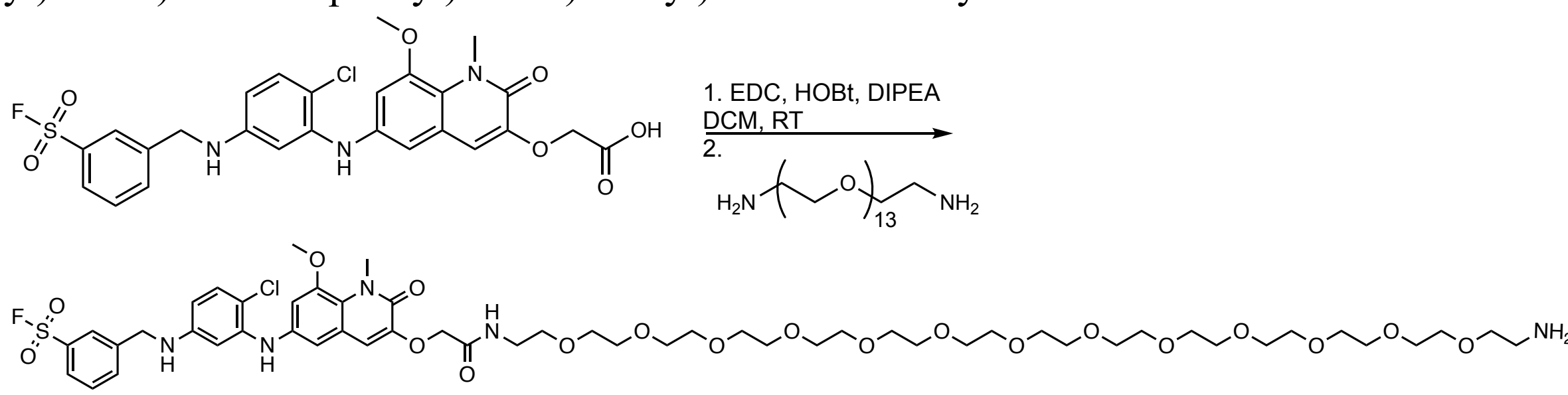

Synthetic procedure performed similar to the one described for EL221, as (23) is a side product of the same reaction. During EL221 HPLC purification, the fraction containing (23) was lyophilized separately.

LC/MS (m/z): Calculated: 1189.49; Found: 1190.99 $[M+H]^+$, 1212.93 $[M+Na]^+$

BDX-PEG13-NH2 (24)- (4aS,6aR,6bS,8aR,12aS,14aR,14bS)-N-(41-amino-3,6,9,12,15,18,21,24,27,30,33,36,39-tridecaoxahentetracontyl)-11-cyano-2,2,6a,6b,9,9,12a-heptamethyl-10,14-dioxo-1,3,4,5,6,6a,6b,7,8,8a,9,10,12a,14,14a,14b-hexadecahydropicene-4a(2H)-carboxamide

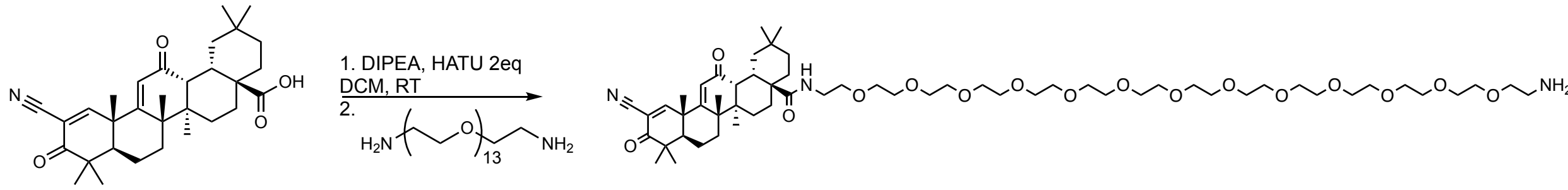

Synthetic procedure performed similar to the one described for EL229, as (24) is a side product of the same reaction. During EL229 HPLC purification, the fraction containing (24) was lyophilized separately.

LC/MS (m/z): Calculated: 1105.70; Found: 1106.78 $[M+H]^+$, 1129.93 $[M+Na]^+$

EL217 (25)- 3,3'-(((((((2,13-dioxo-6,9-dioxa-3,12-diazatetradecane-1,14-diyl)bis(oxy))bis(8-methoxy-1-methyl-2-oxo-1,2-dihydroquinoline-3,6-diyl))bis(azanediyl))bis(4-chloro-3,1-phenylene))bis(azanediyl))bis(methylene))dibenzenesulfonyl fluoride

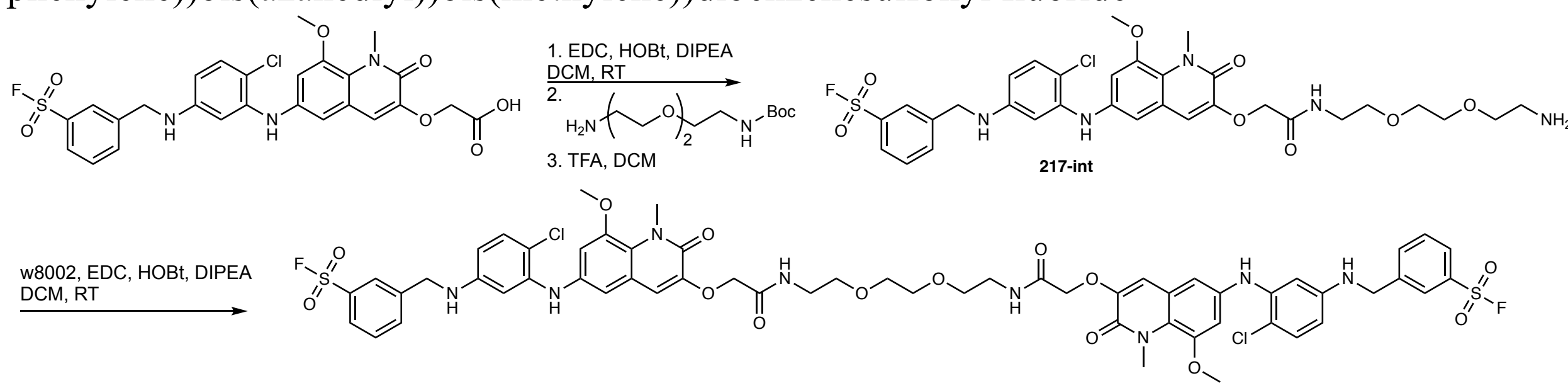

Synthetic procedure performed similar to the one described for EL228, using *tert*-butyl (2-(2-(2-aminoethoxy)ethoxy)ethyl)carbamate as the linker in the second step.

HR-MS (m/z): Calculated: 1262.29; Found: 1285.2777 $[M+Na]^+$

EL218 (26)- 3,3'-(((((((2,19-dioxo-6,9,12,15-tetraoxa-3,18-diazaicosane-1,20-diyl)bis(oxy))bis(8-methoxy-1-methyl-2-oxo-1,2-dihydroquinoline-3,6-diyl))bis(azanediyl))bis(4-chloro-3,1-phenylene))bis(azanediyl))bis(methylene))dibenzenesulfonyl fluoride

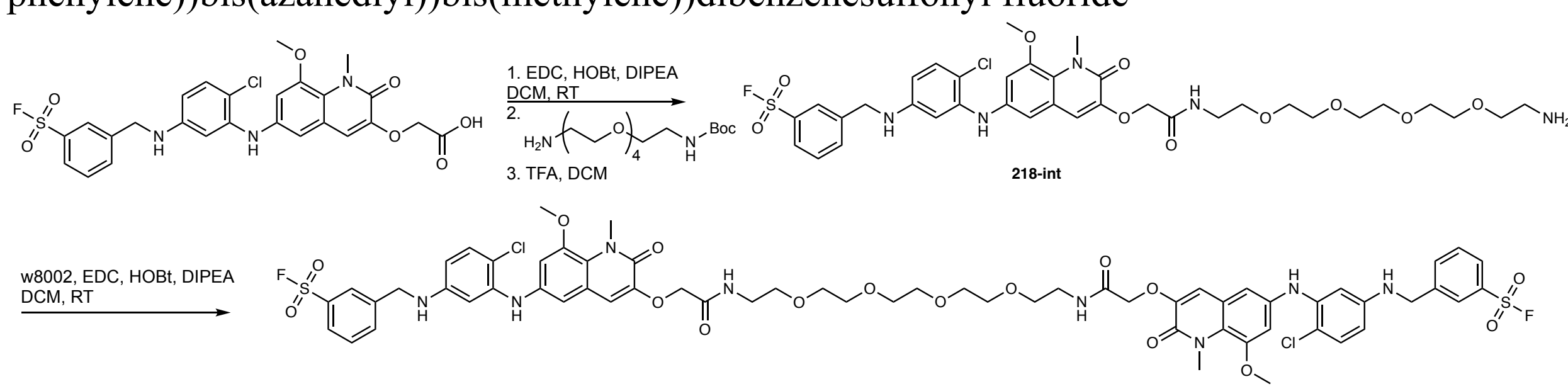

Synthetic procedure performed similar to the one described for EL228, using *tert-butyl (14-amino-3,6,9,12-tetraoxatetradecyl)carbamate* as the linker in the second step.

HR-MS (m/z): Calculated: 1350.34; Found: 1373.3314 $[M+Na]^+$

EL227 (27)- 3,3'-(((((((2,31-dioxo-6,9,12,15,18,21,24,27-octaoxa-3,30-diazadotriacontane-1,32-diyl)bis(oxy))bis(8-methoxy-1-methyl-2-oxo-1,2-dihydroquinoline-3,6-diyl))bis(azanediyl))bis(4-chloro-3,1-phenylene))bis(azanediyl))bis(methylene))dibenzenesulfonyl fluoride

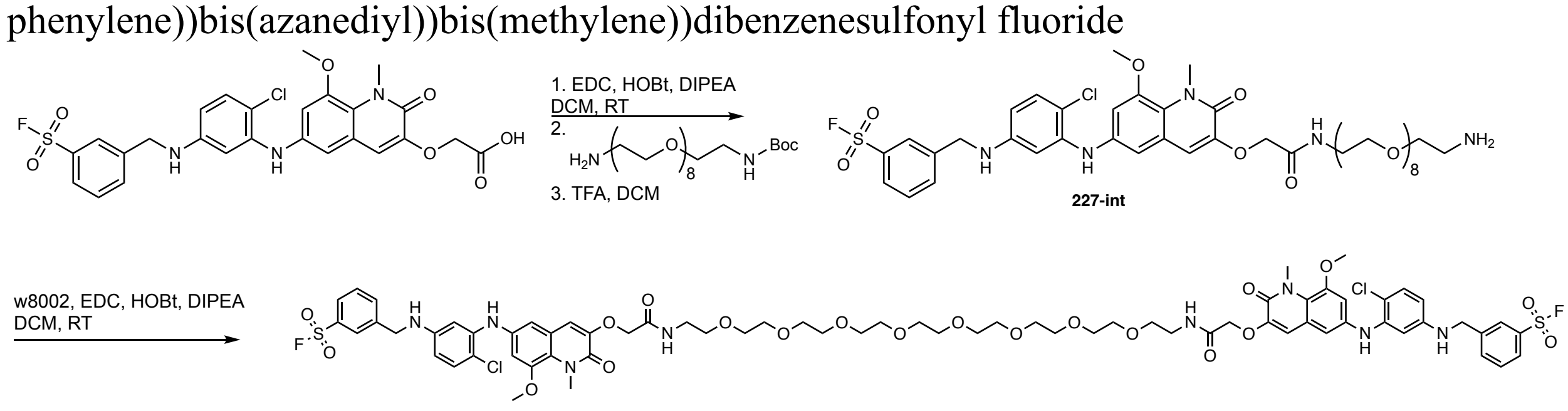

Synthetic procedure performed similar to the one described for EL228, using *tert-butyl (26-amino-3,6,9,12,15,18,21,24-octaoxahexacosyl)carbamate* as the linker in the second step.

HR-MS (m/z): Calculated: 1526.44; Found: 1549.4396 $[M+Na]^+$

EL226 (28)- 3,3'-(((((((2,34-dioxo-6,9,12,15,18,21,24,27,30-nonaoxa-3,33-diazapentatriacontane-1,35-diyl)bis(oxy))bis(8-methoxy-1-methyl-2-oxo-1,2-dihydroquinoline-3,6-diyl))bis(azanediyl))bis(4-chloro-3,1-phenylene))bis(azanediyl))bis(methylene))dibenzenesulfonyl fluoride

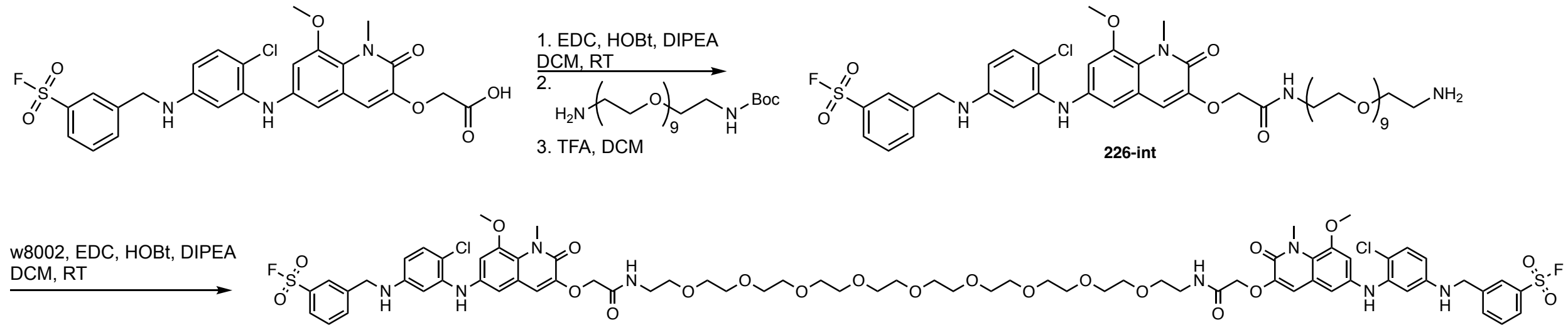

Synthetic procedure performed similar to the one described for EL228, using *tert-butyl (29-amino-3,6,9,12,15,18,21,24,27-nonaoxanonacosyl)carbamate* as the linker in the second step.

HR-MS (m/z): Calculated: 1570.47; Found: 1593.4633 $[M+Na]^+$

EL224 (29)- 3,3'-(((((((2,37-dioxo-6,9,12,15,18,21,24,27,30,33-decaoxa-3,36-diazaoctatriacontane-1,38-diyl)bis(oxy))bis(8-methoxy-1-methyl-2-oxo-1,2-dihydroquinoline-3,6-diyl))bis(azanediyl))bis(4-chloro-3,1-phenylene))bis(azanediyl))bis(methylene))dibenzenesulfonyl fluoride

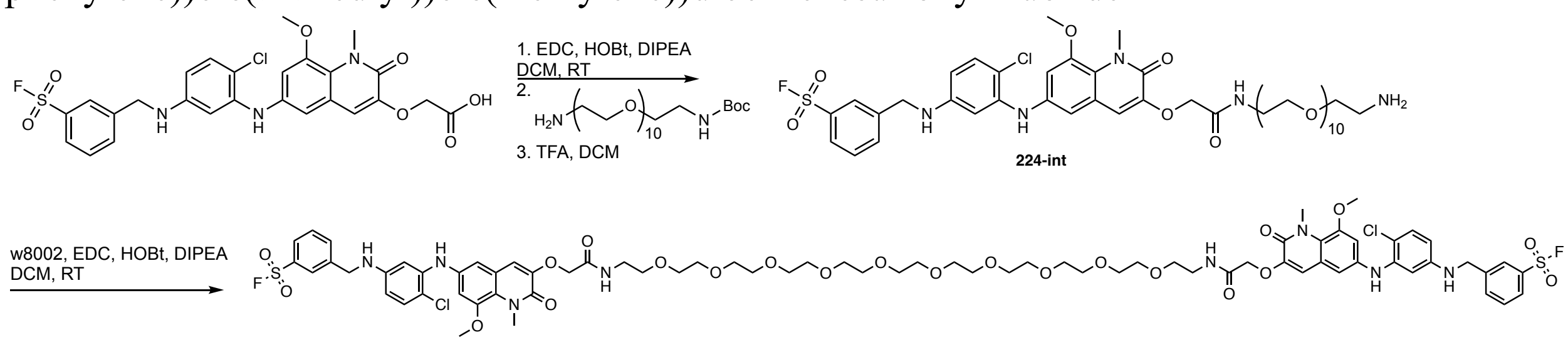

Synthetic procedure performed similar to the one described for EL228, using *tert-butyl (32-amino-3,6,9,12,15,18,21,24,27,30-decaoxadotriacontyl)carbamate* as the linker in the second step.

HR-MS (m/z): Calculated: 1614.50; Found: 1637.4777 $[M+Na]^+$

NMR spectra:

EL133:

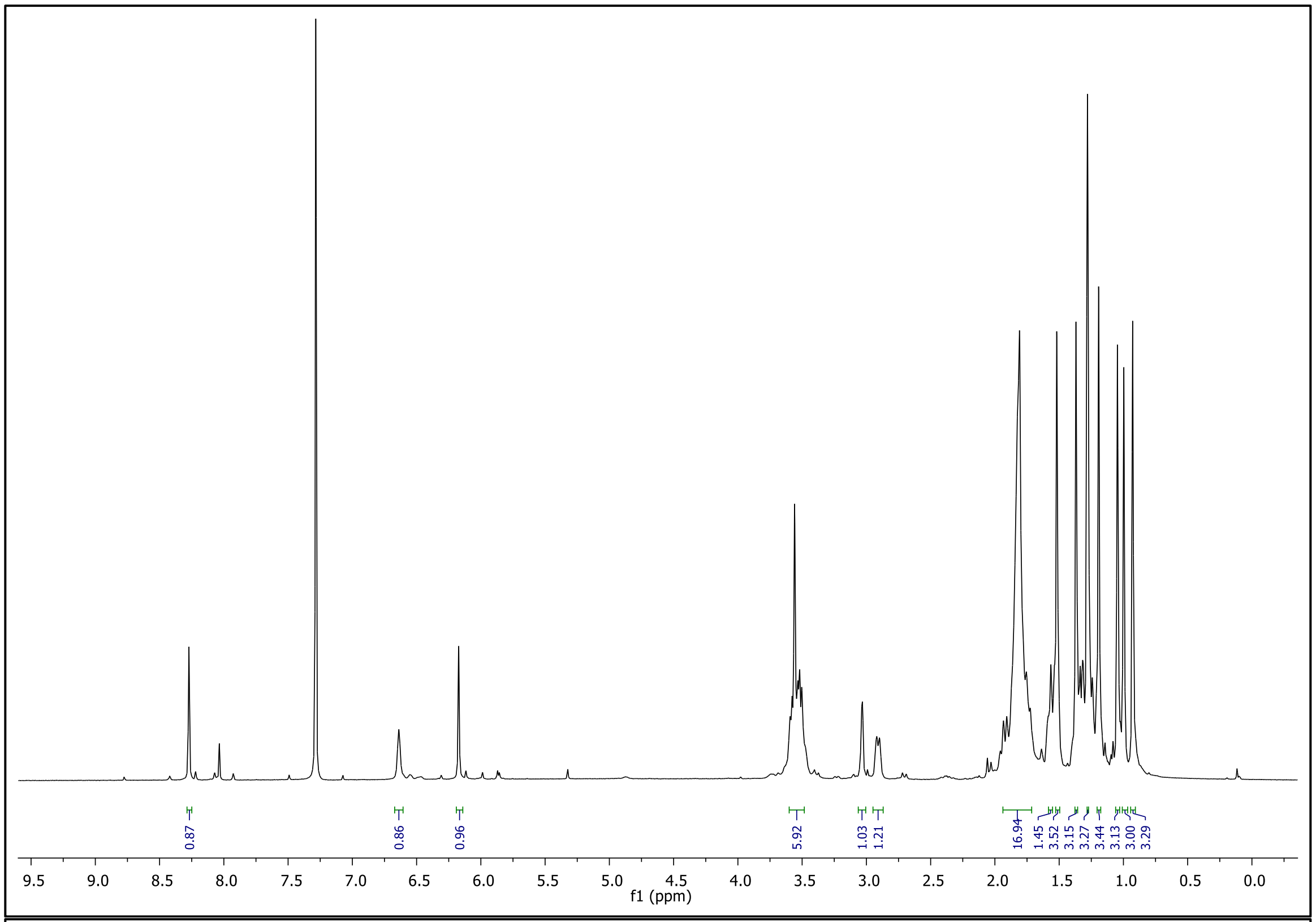

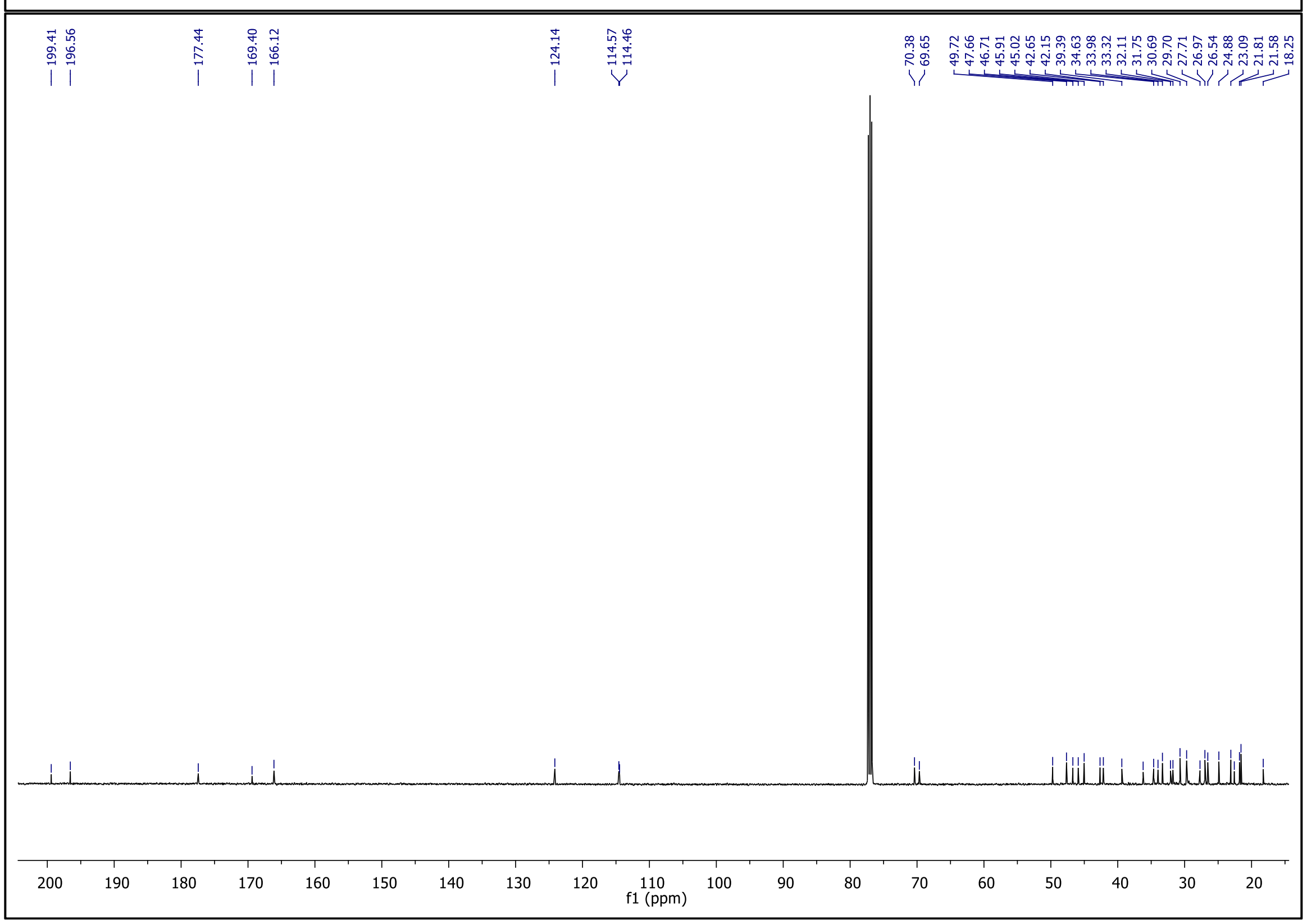

EL164:

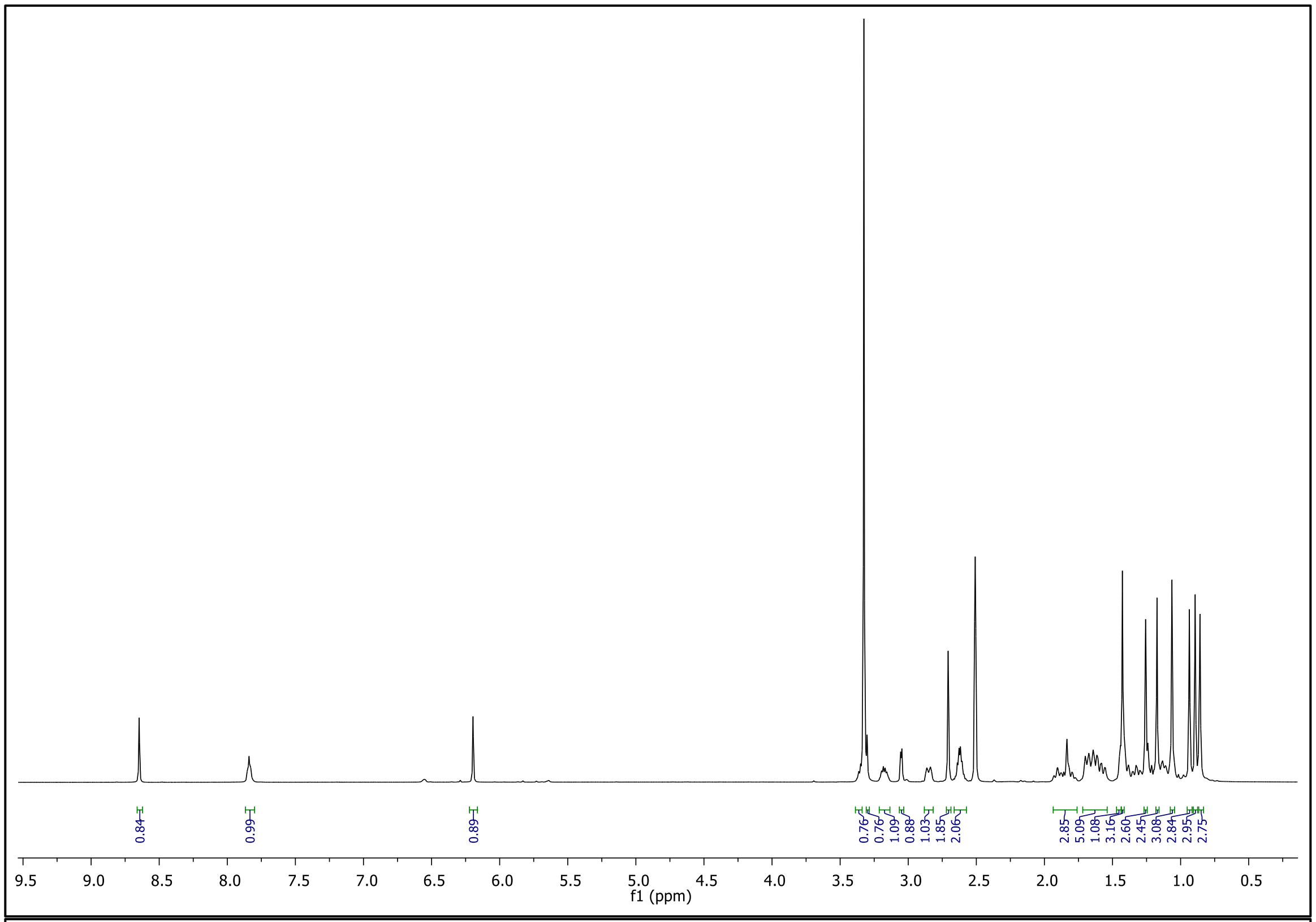

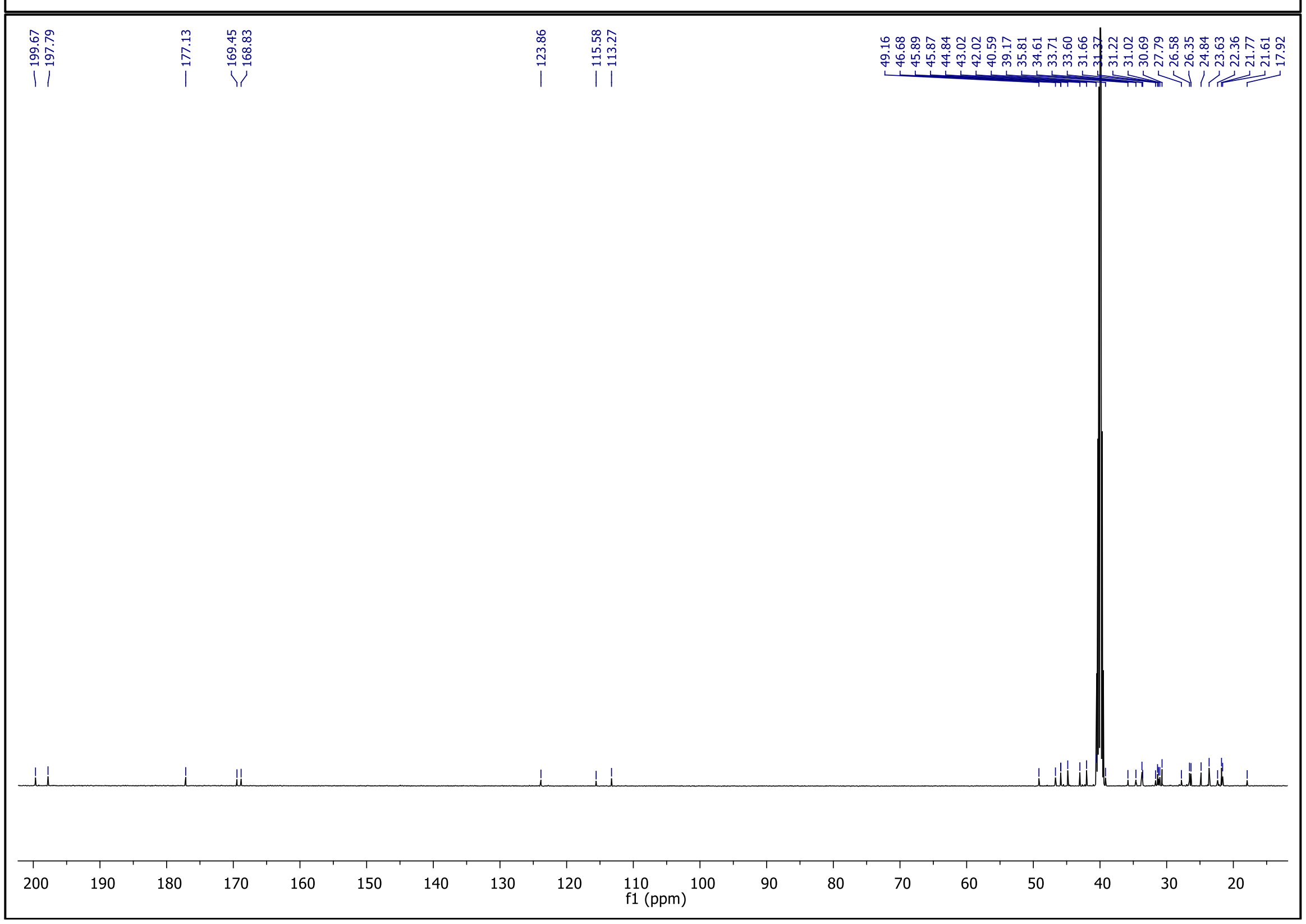

EL165:

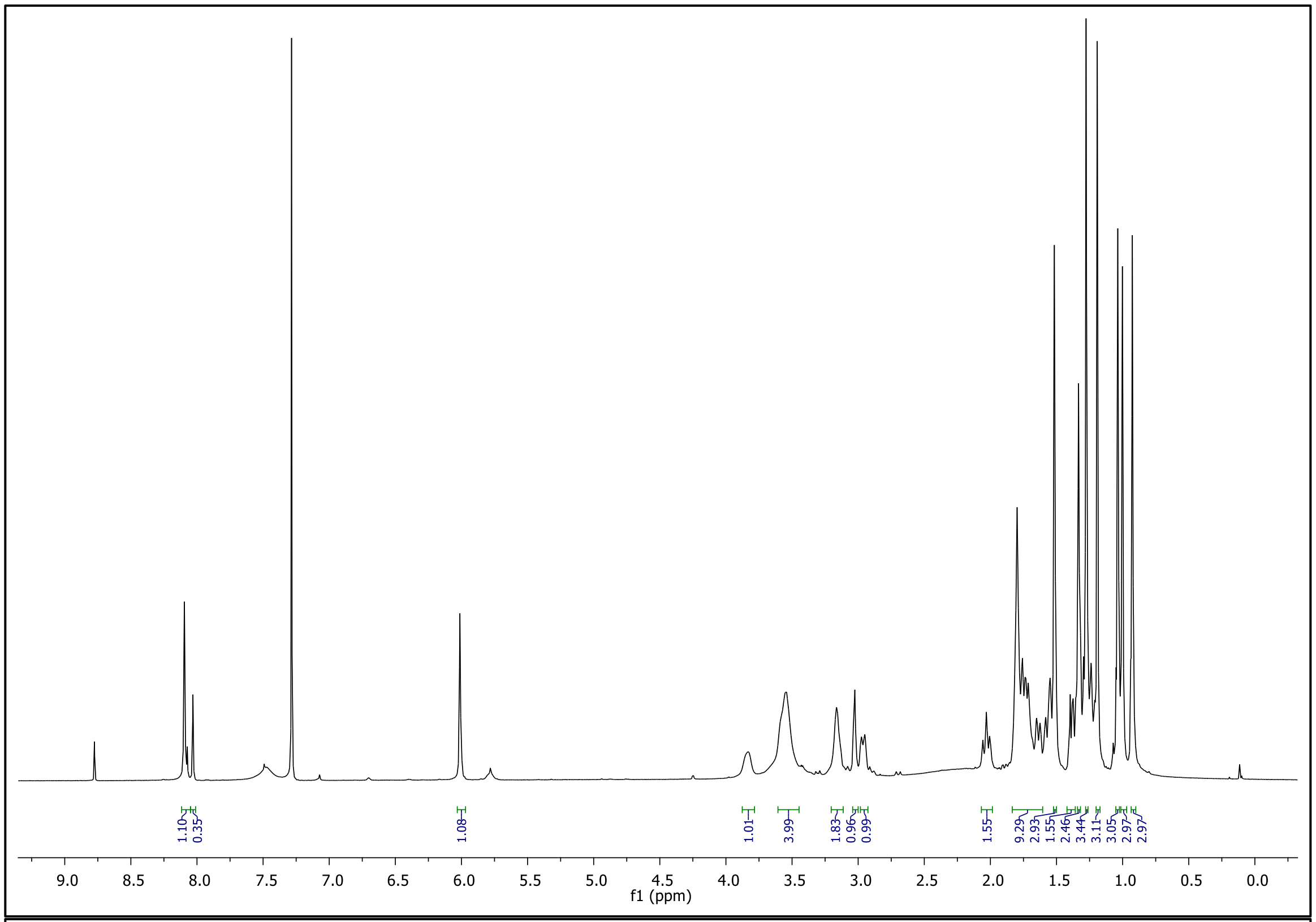
1.10
0.35
1.08
1.01
3.99
1.83
0.96
0.99
1.55
9.29
2.93
1.55
2.46
3.44
3.11
3.05
2.97
2.97
9.0 8.5 8.0 7.5 7.0 6.5 6.0 5.5 5.0 4.5 4.0 3.5 3.0 2.5 2.0 1.5 1.0 0.5 0.0
f1 (ppm)

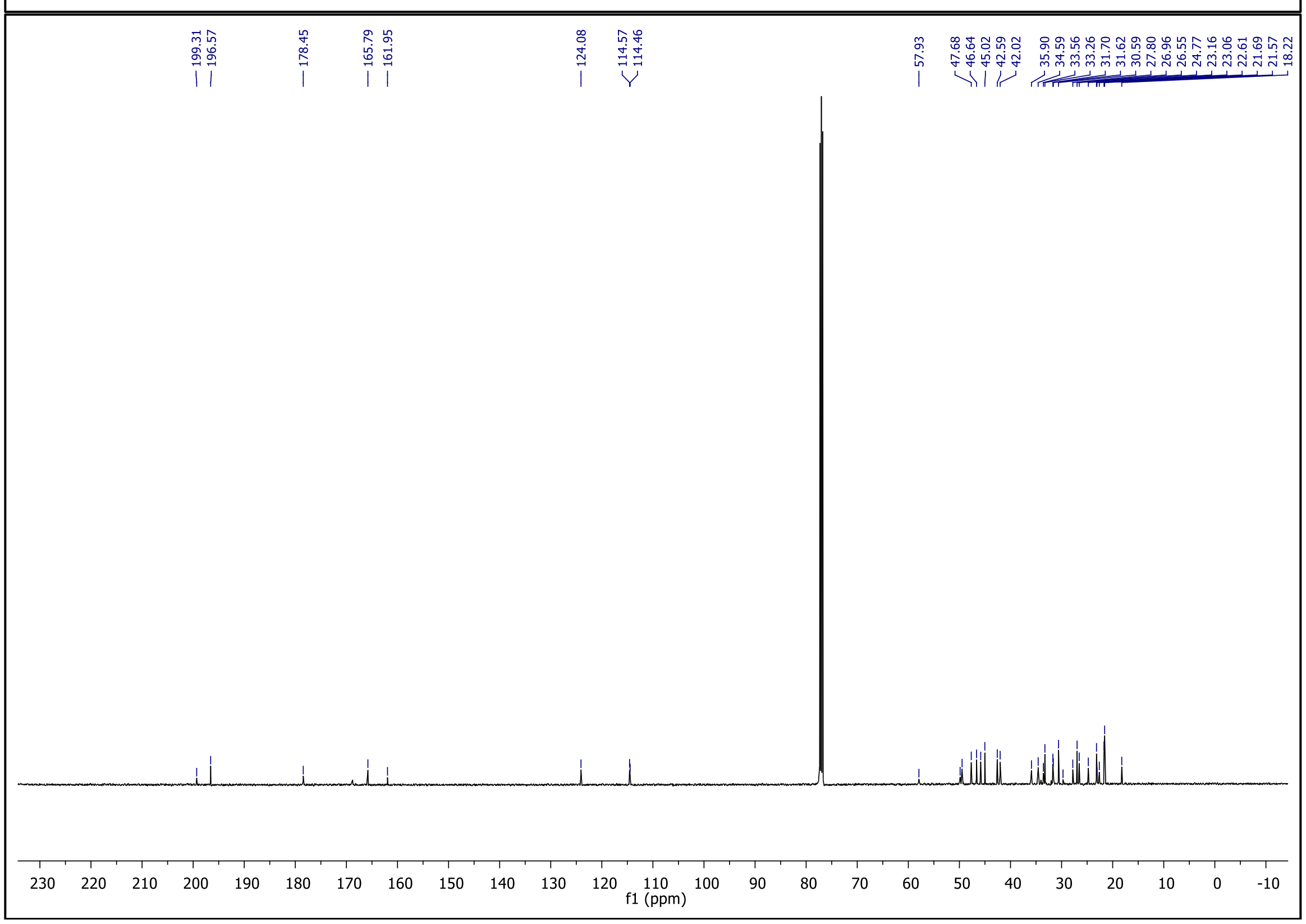
199.31
196.57
178.45
165.79
161.95
124.08
114.57
114.46
57.93
47.68
46.64
45.02
42.59
42.02
35.90
34.59
33.56
33.26
31.70
31.62
30.59
27.80
26.96
26.55
24.77
23.16
23.06
22.61
21.69
21.57
18.22
230 220 210 200 190 180 170 160 150 140 130 120 110 100 90 80 70 60 50 40 30 20 10 0 -10
f1 (ppm)

EL229:

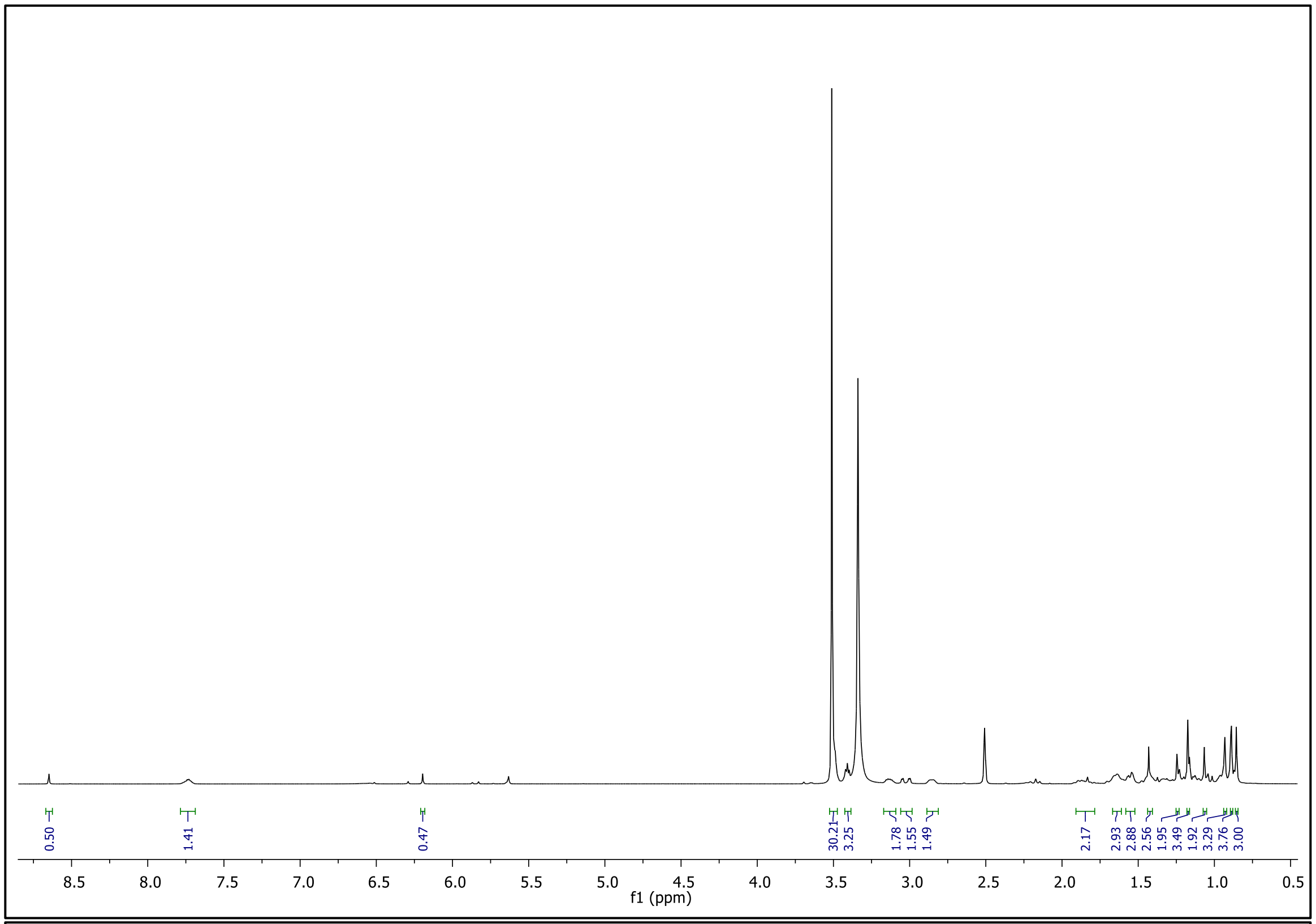
0.50
1.41
0.47
30.21
3.25
1.78
1.55
1.49
2.17
2.93
2.88
2.56
1.95
3.49
1.92
3.29
3.76
3.00
8.5
8.0
7.5
7.0
6.5
6.0
5.5
5.0
4.5
4.0
3.5
3.0
2.5
2.0
1.5
1.0
0.5
f1 (ppm)

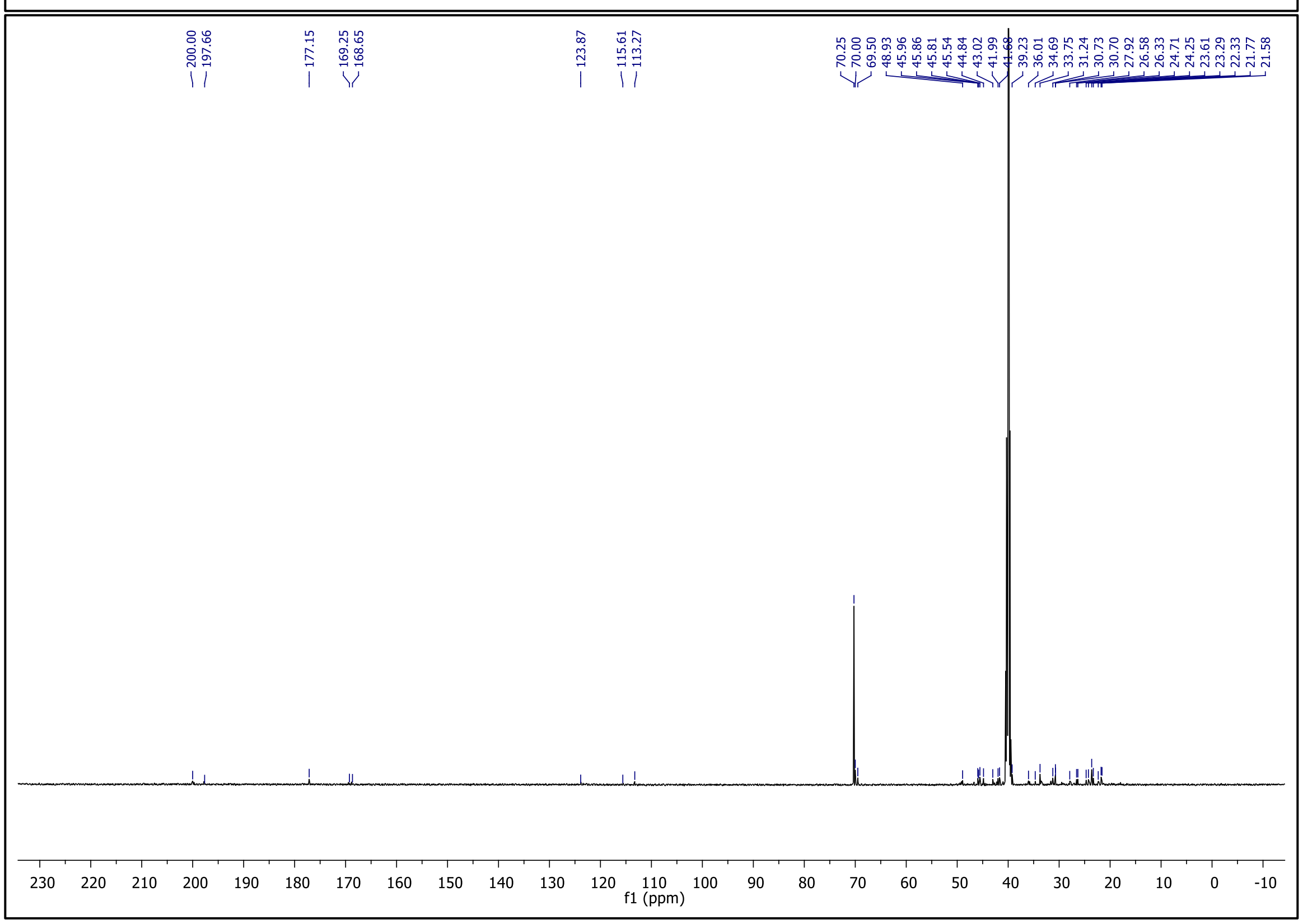
200.00
197.66
177.15
169.25
168.65
123.87
115.61
113.27
70.25
70.00
69.50
48.93
45.96
45.86
45.81
45.54
44.84
43.02
41.99
41.68
39.23
36.01
34.69
33.75
31.24
30.73
30.70
27.92
26.58
26.33
24.71
24.25
23.61
23.29
22.33
21.77
21.58
230
220
210
200
190
180
170
160
150
140
130
120
110
100
90
80
70
60
50
40
30
20
10
0
-10
f1 (ppm)

EL228:

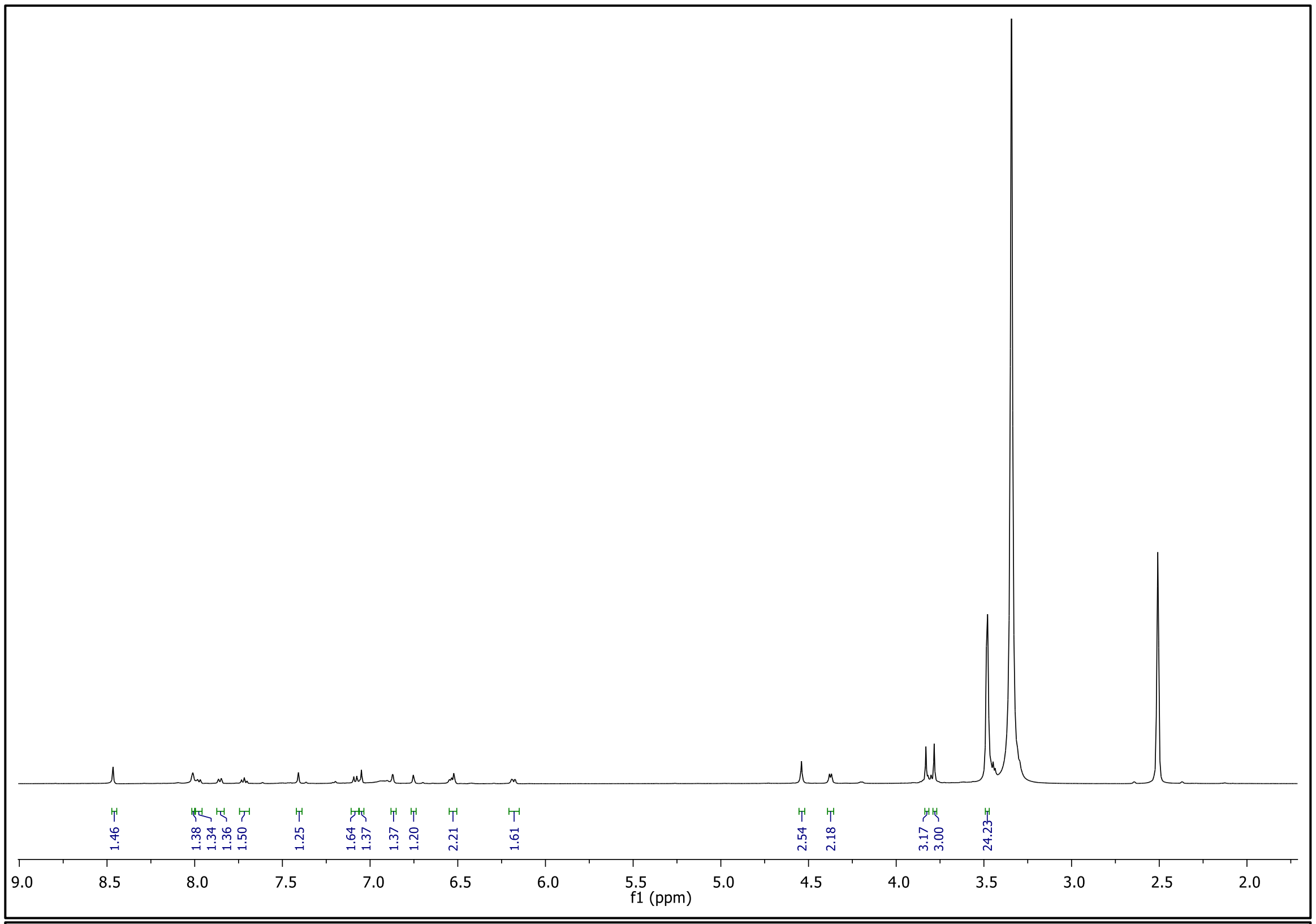

1.46
1.38
1.34
1.36
1.50
1.25
1.64
1.37
1.37
1.20
2.21
1.61
2.54
2.18
3.17
3.00
24.23
f1 (ppm)

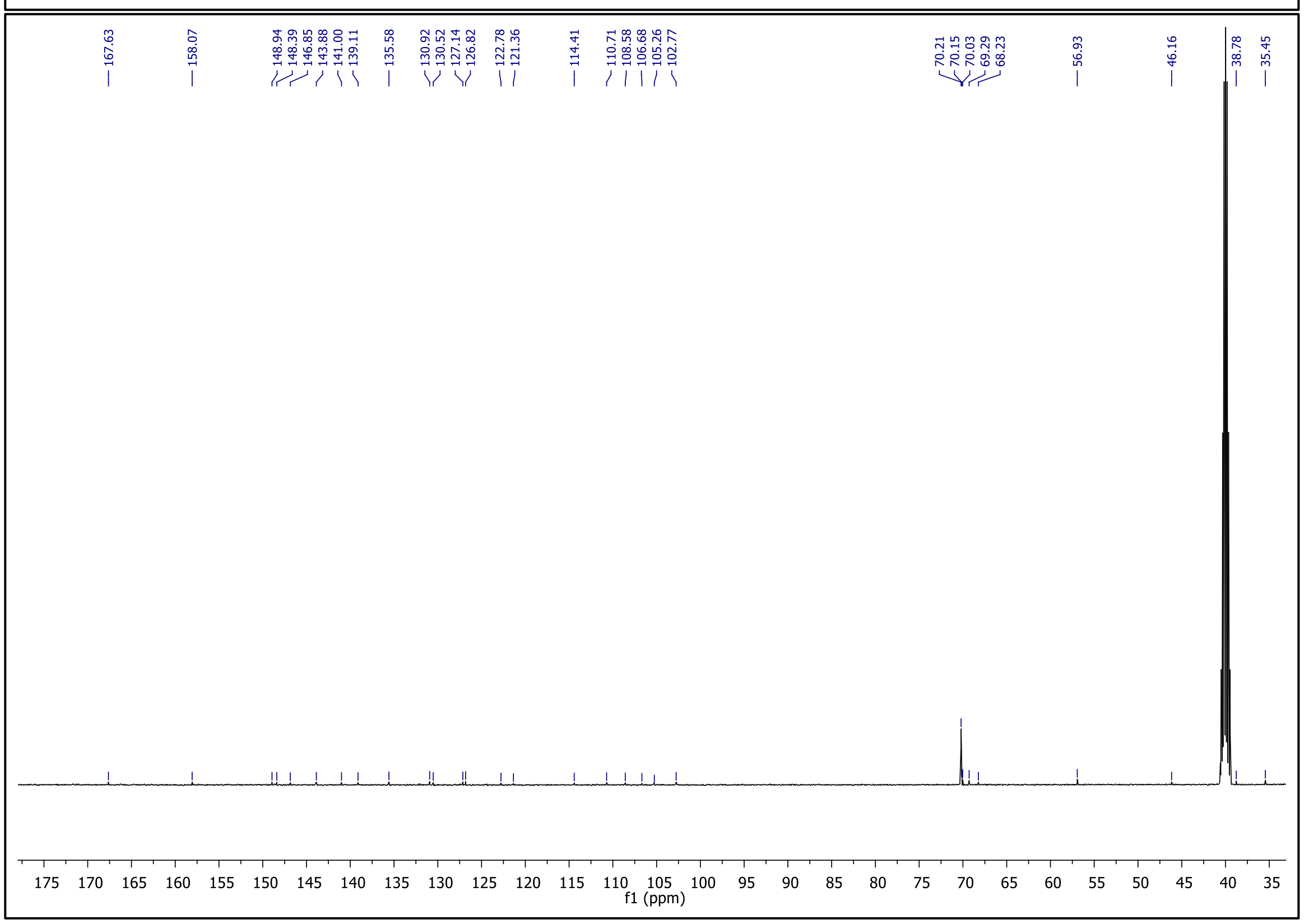

167.63
158.07
148.94
148.39
146.85
143.88
141.00
139.11
135.58
130.92
130.52
127.14
126.82
122.78
121.36
114.41
110.71
108.58
106.68
105.26
102.77
70.21
70.15
70.03
69.29
68.23
56.93
46.16
38.78
35.45
f1 (ppm)

EL221:

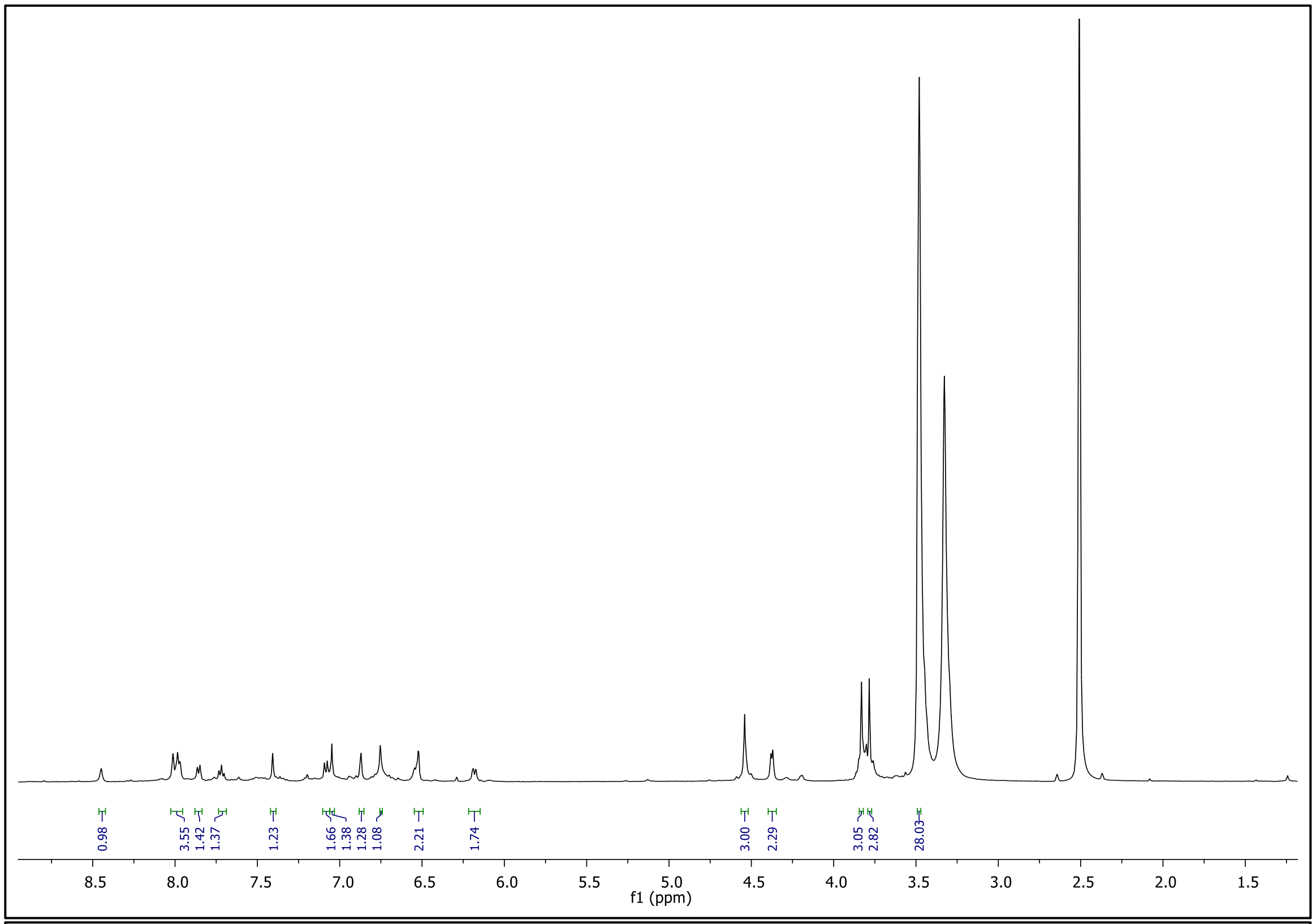
0.98
3.55
1.42
1.37
1.23
1.66
1.38
1.28
1.08
2.21
1.74
3.00
2.29
3.05
2.82
28.03
8.5 8.0 7.5 7.0 6.5 6.0 5.5 5.0 4.5 4.0 3.5 3.0 2.5 2.0 1.5
f1 (ppm)

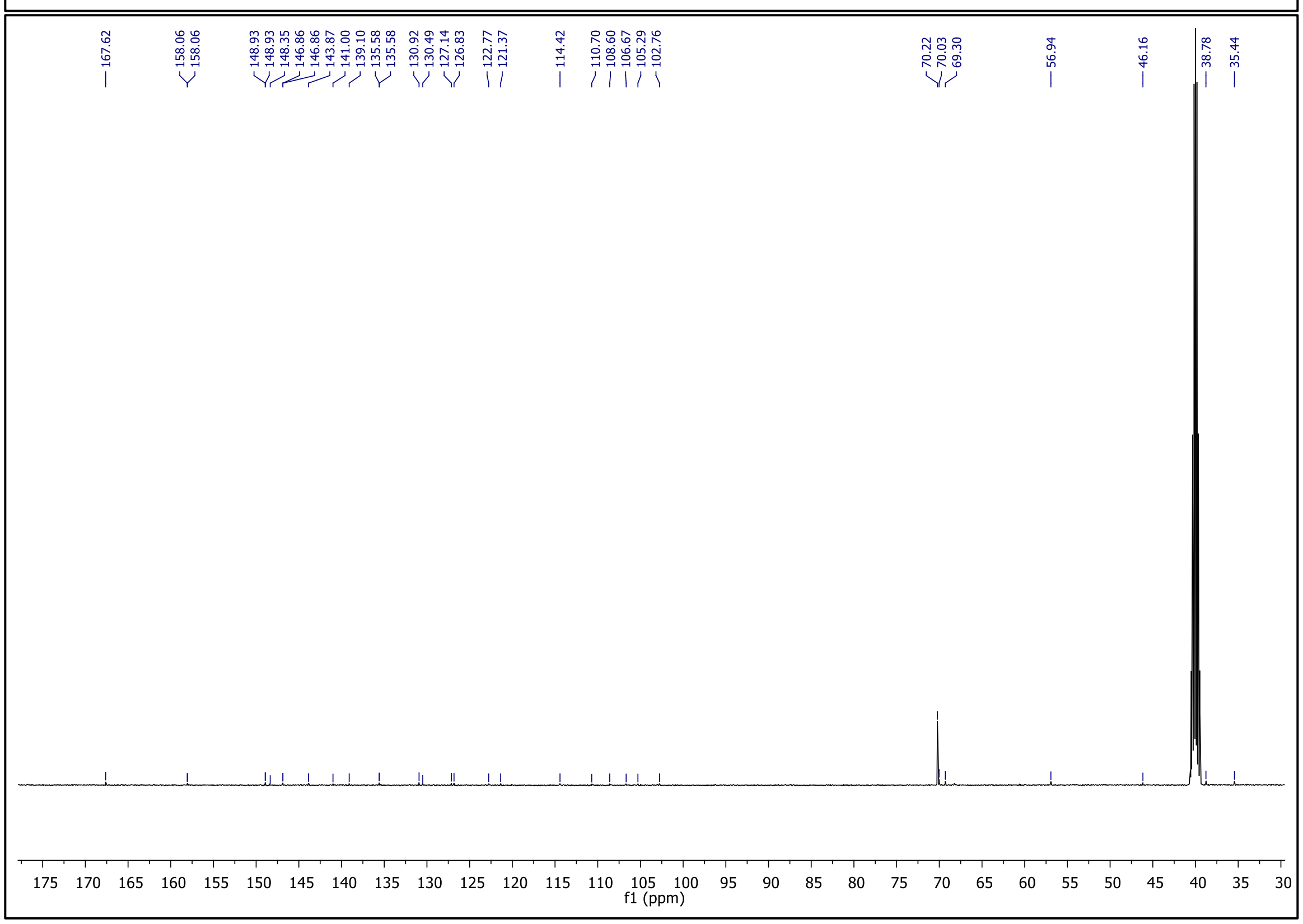
167.62
158.06
158.06
148.93
148.93
148.35
146.86
146.86
143.87
141.00
139.10
135.58
135.58
130.92
130.49
127.14
126.83
122.77
121.37
114.42
110.70
108.60
106.67
105.29
102.76
70.22
70.03
69.30
56.94
46.16
38.78
35.44
175 170 165 160 155 150 145 140 135 130 125 120 115 110 105 100 95 90 85 80 75 70 65 60 55 50 45 40 35 30
f1 (ppm)

EL225:

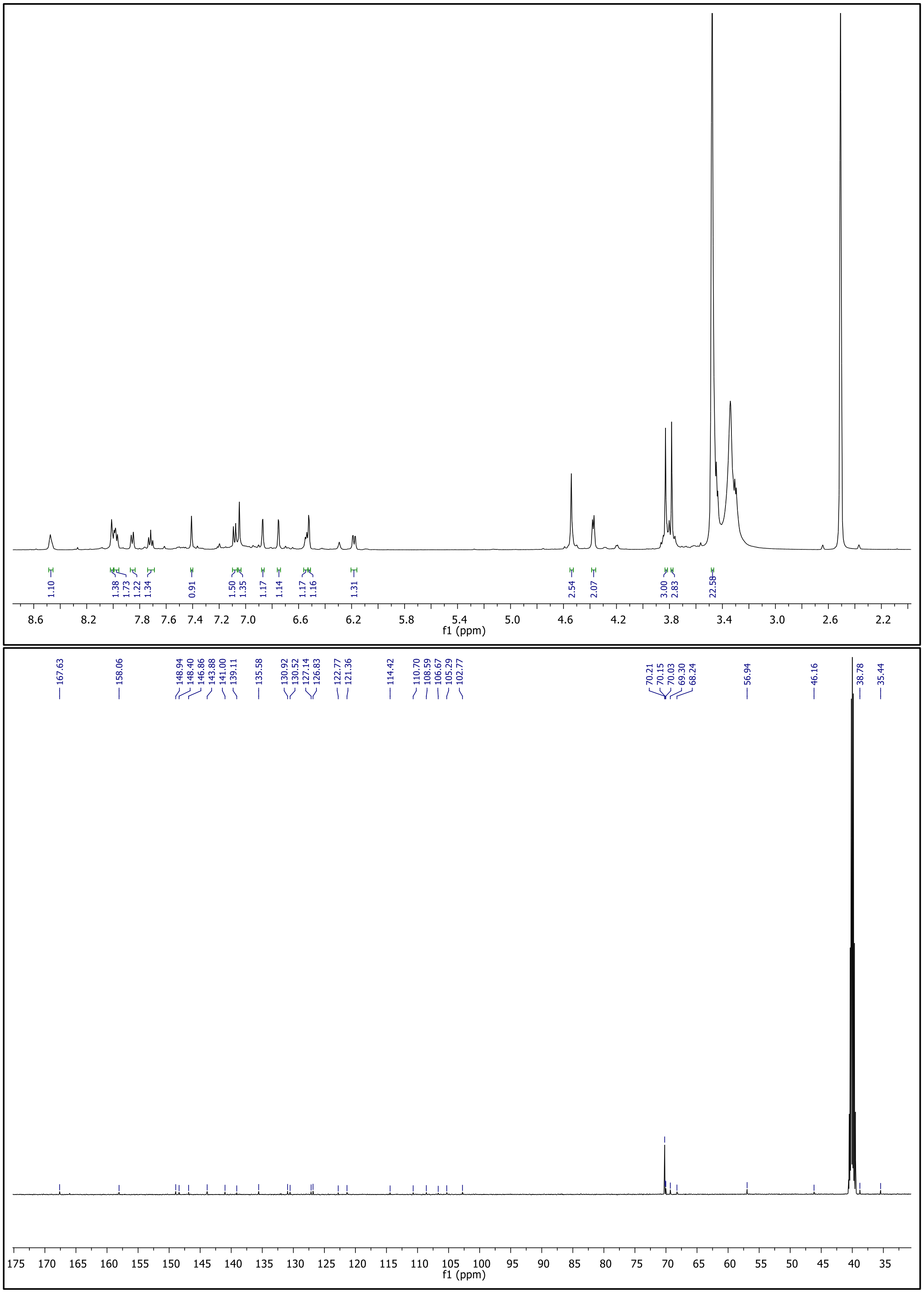
1.10
1.38
1.73
1.22
1.34
0.91
1.50
1.35
1.17
1.14
1.17
1.16
1.31
2.54
2.07
3.00
2.83
22.58
8.6
8.2
7.8
7.6
7.4
7.2
7.0
6.6
6.2
5.8
5.4
5.0
4.6
4.2
3.8
3.4
3.0
2.6
2.2
f1 (ppm)
167.63
158.06
148.94
148.40
146.86
143.88
141.00
139.11
135.58
130.92
130.52
127.14
126.83
122.77
121.36
114.42
110.70
108.59
106.67
105.29
102.77
70.21
70.15
70.03
69.30
68.24
56.94
46.16
38.78
35.44
175
170
165
160
155
150
145
140
135
130
125
120
115
110
105
100
95
90
85
80
75
70
65
60
55
50
45
40
35
f1 (ppm)

OS-47B:

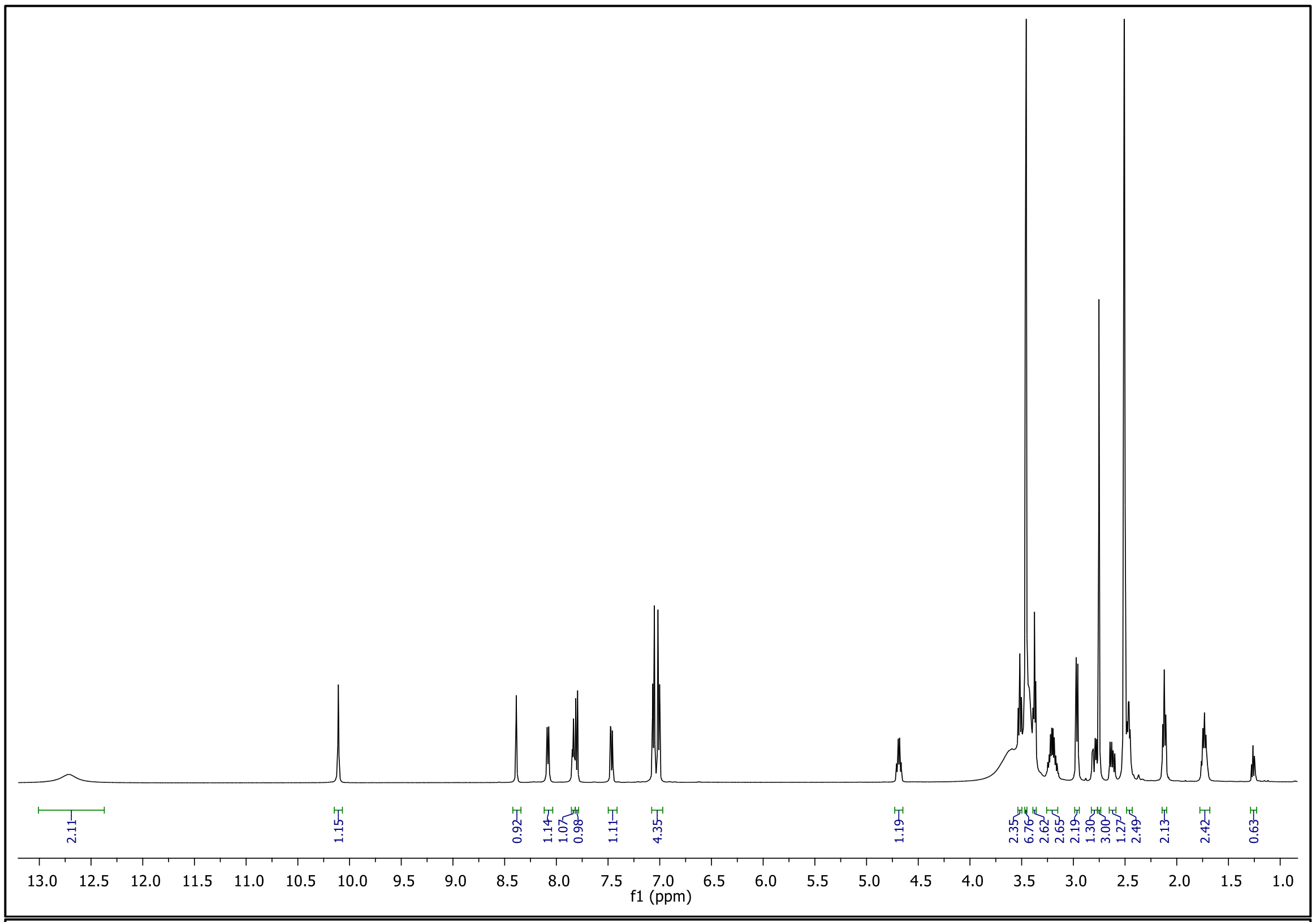

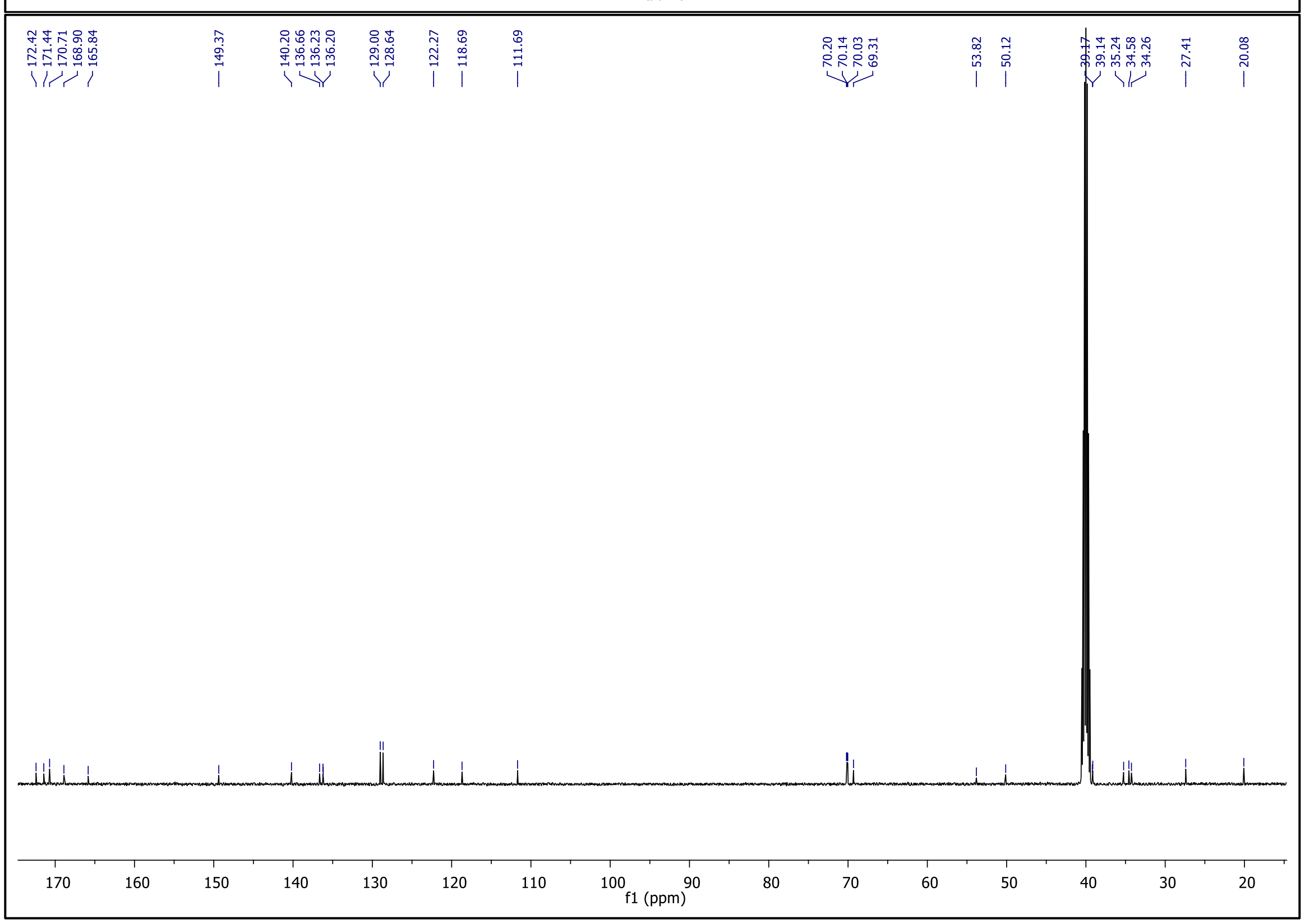

EL132:

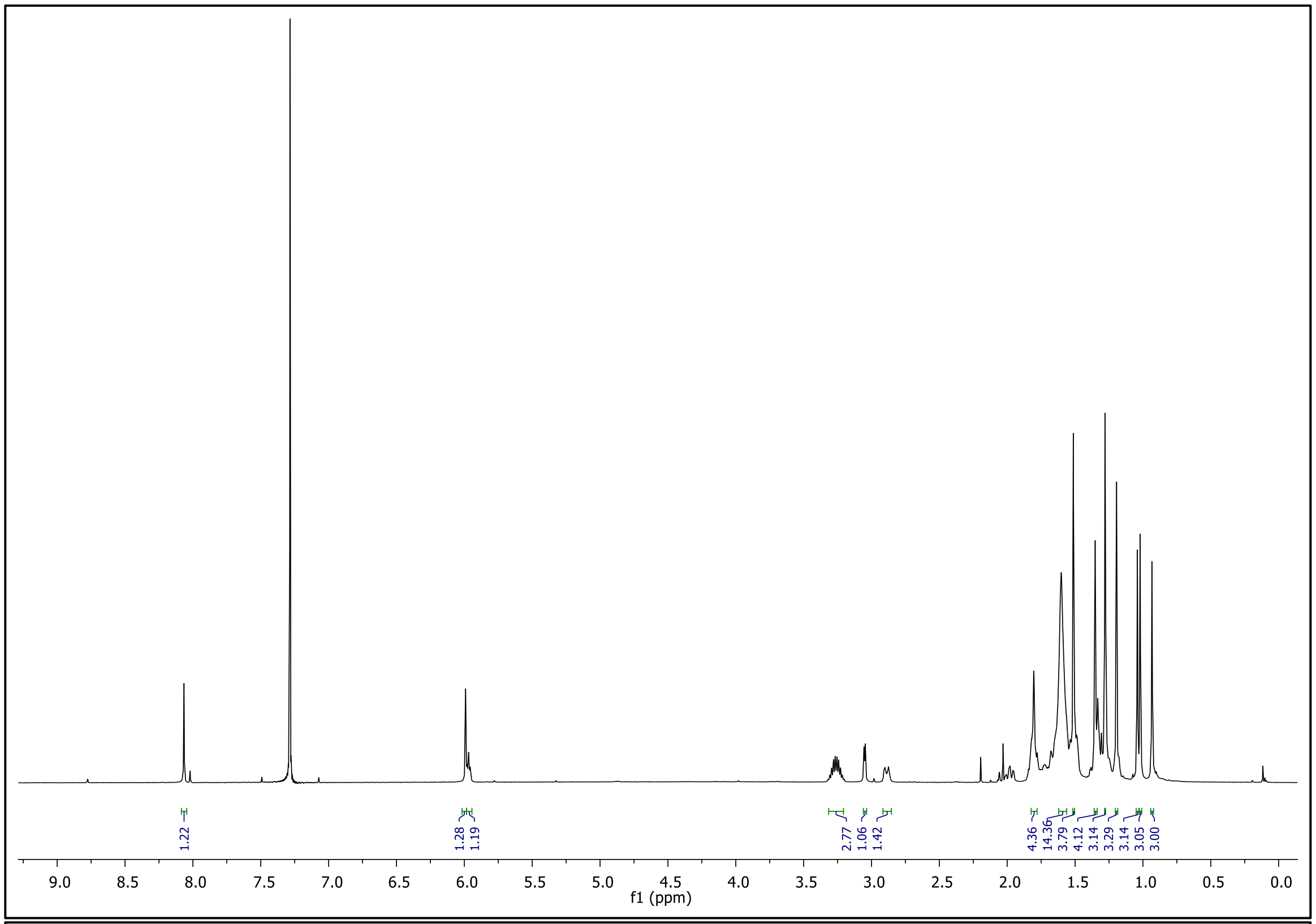
1.22
1.28
1.19
2.77
1.06
1.42
4.36
14.36
3.79
4.12
3.14
3.29
3.14
3.05
3.00
9.0
8.5
8.0
7.5
7.0
6.5
6.0
5.5
5.0
4.5
4.0
3.5
3.0
2.5
2.0
1.5
1.0
0.5
0.0
f1 (ppm)

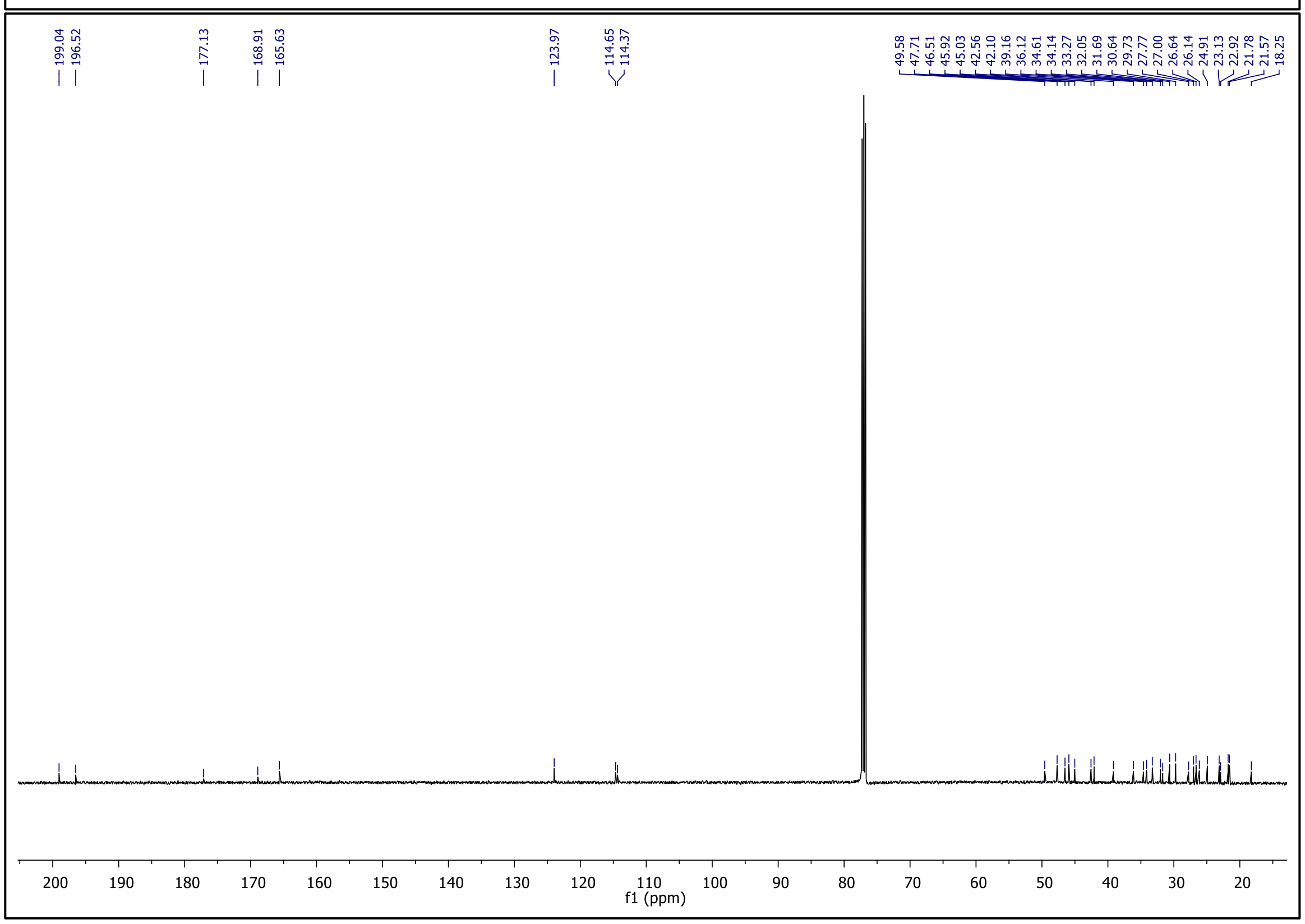
199.04
196.52
177.13
168.91
165.63
123.97
114.65
114.37
49.58
47.71
46.51
45.92
45.03
42.56
42.10
39.16
36.12
34.61
34.14
33.27
32.05
31.69
30.64
29.73
27.77
27.00
26.64
26.14
24.91
23.13
22.92
21.78
21.57
18.25
200
190
180
170
160
150
140
130
120
110
100
90
80
70
60
50
40
30
20
f1 (ppm)

EL230:

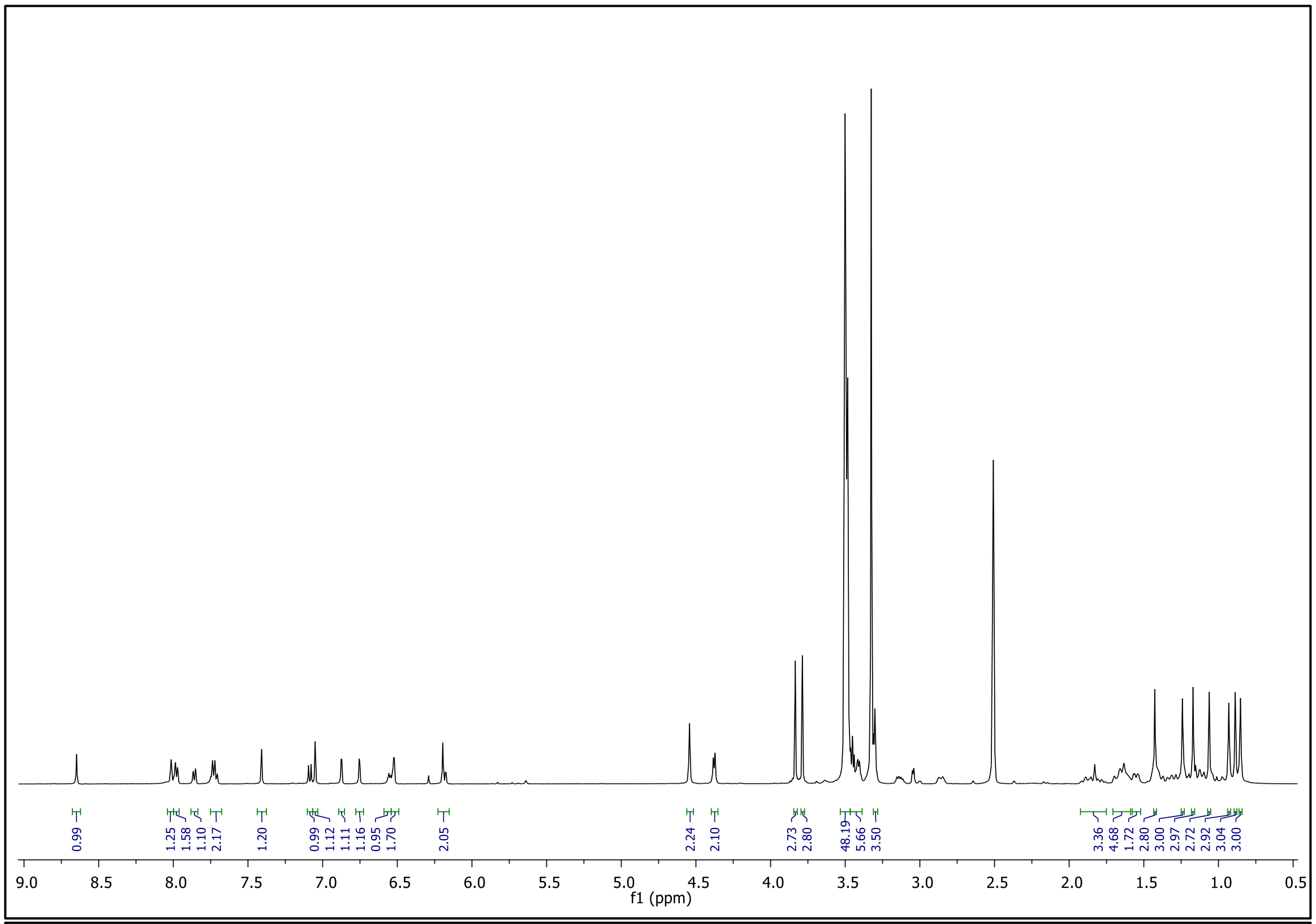

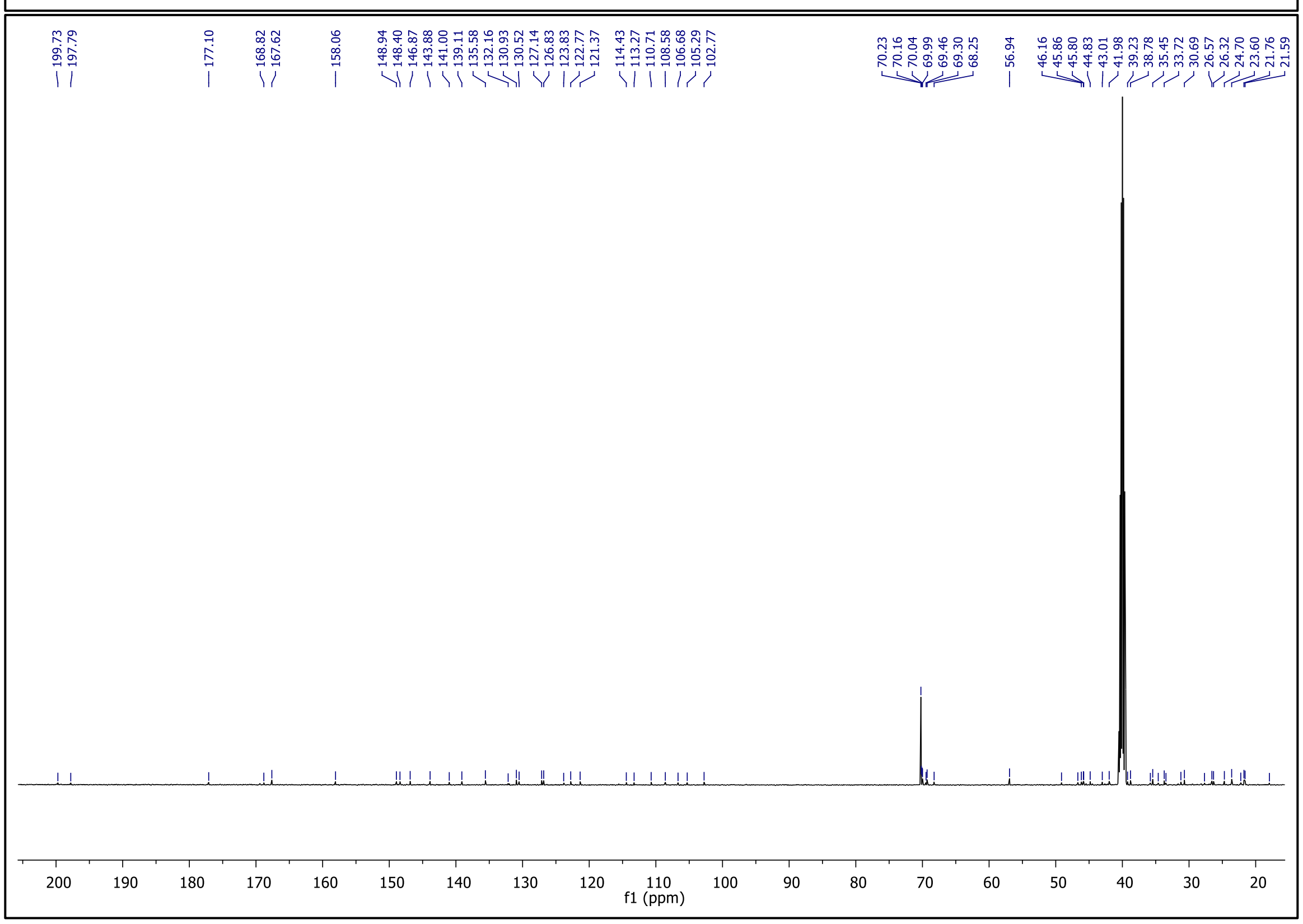

NQO2 amine ligand:

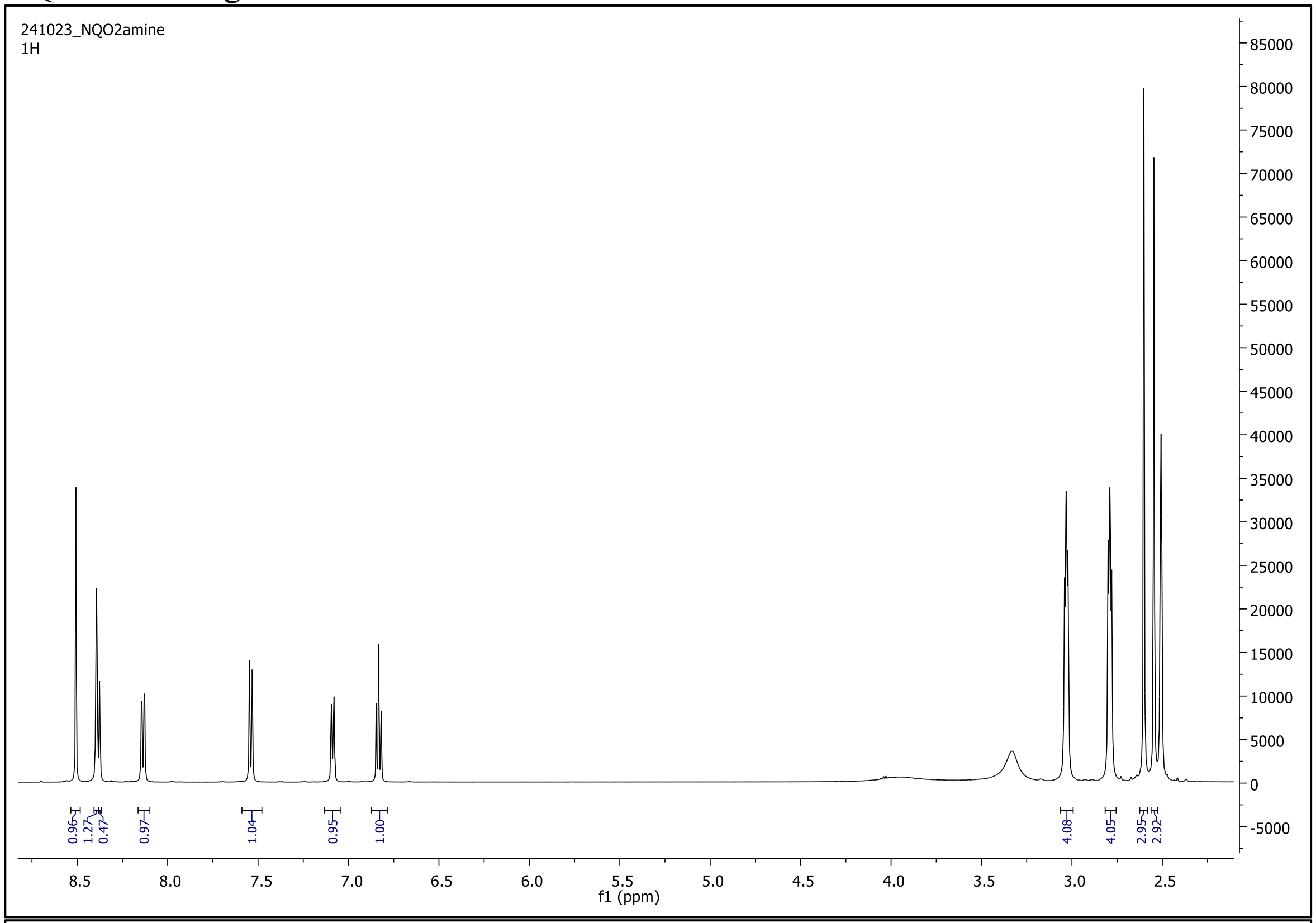

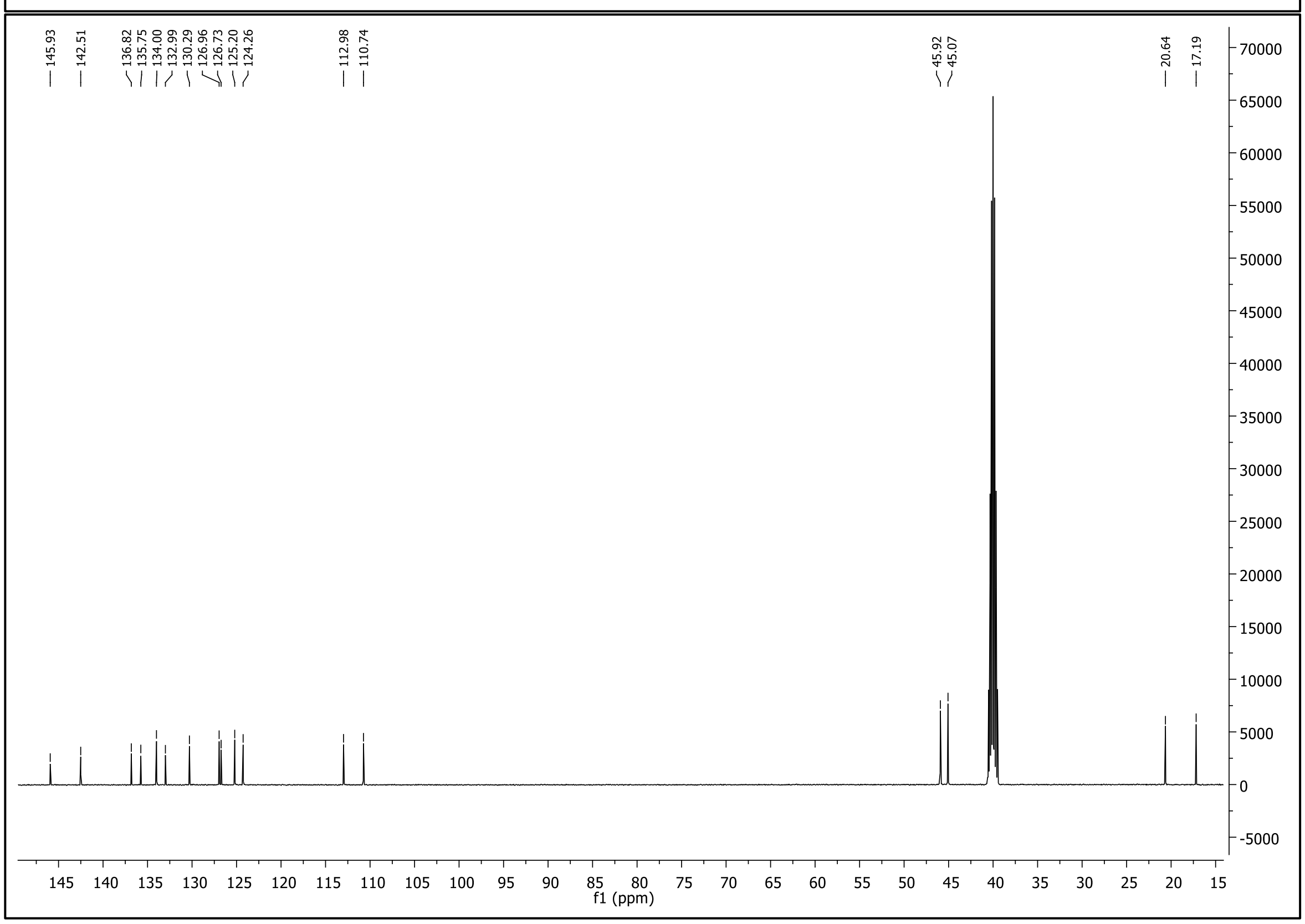

EL241:

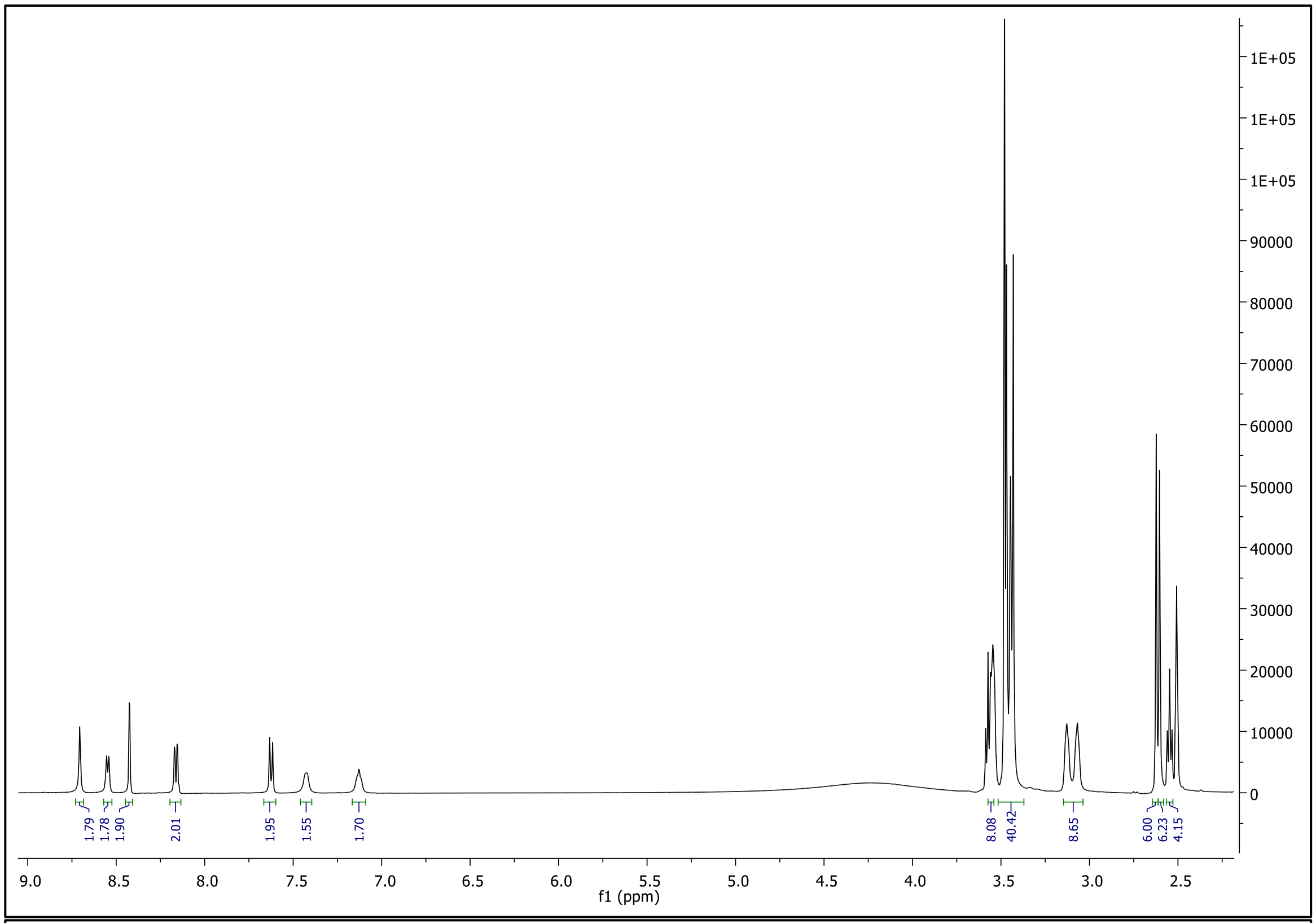

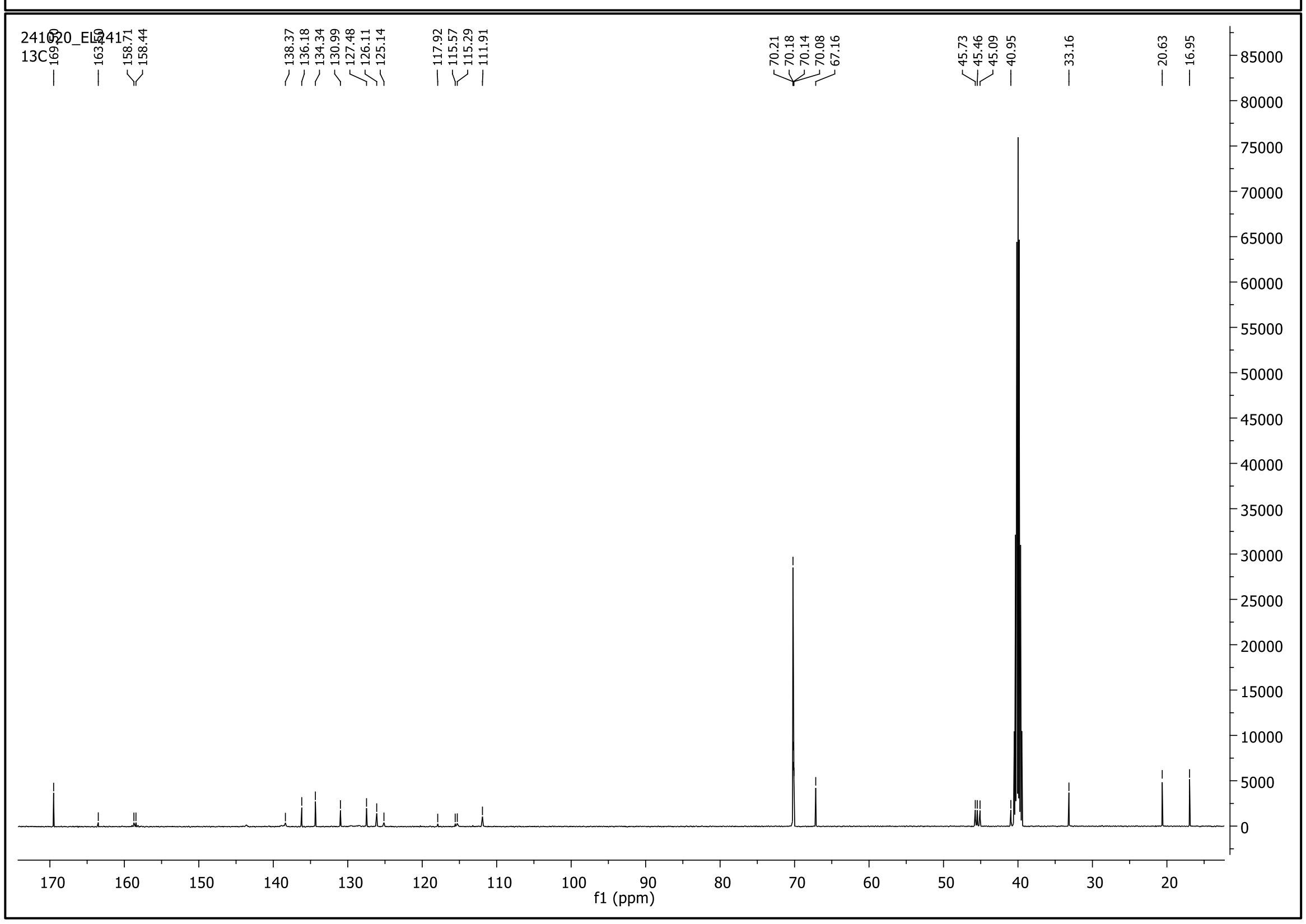

EL246:

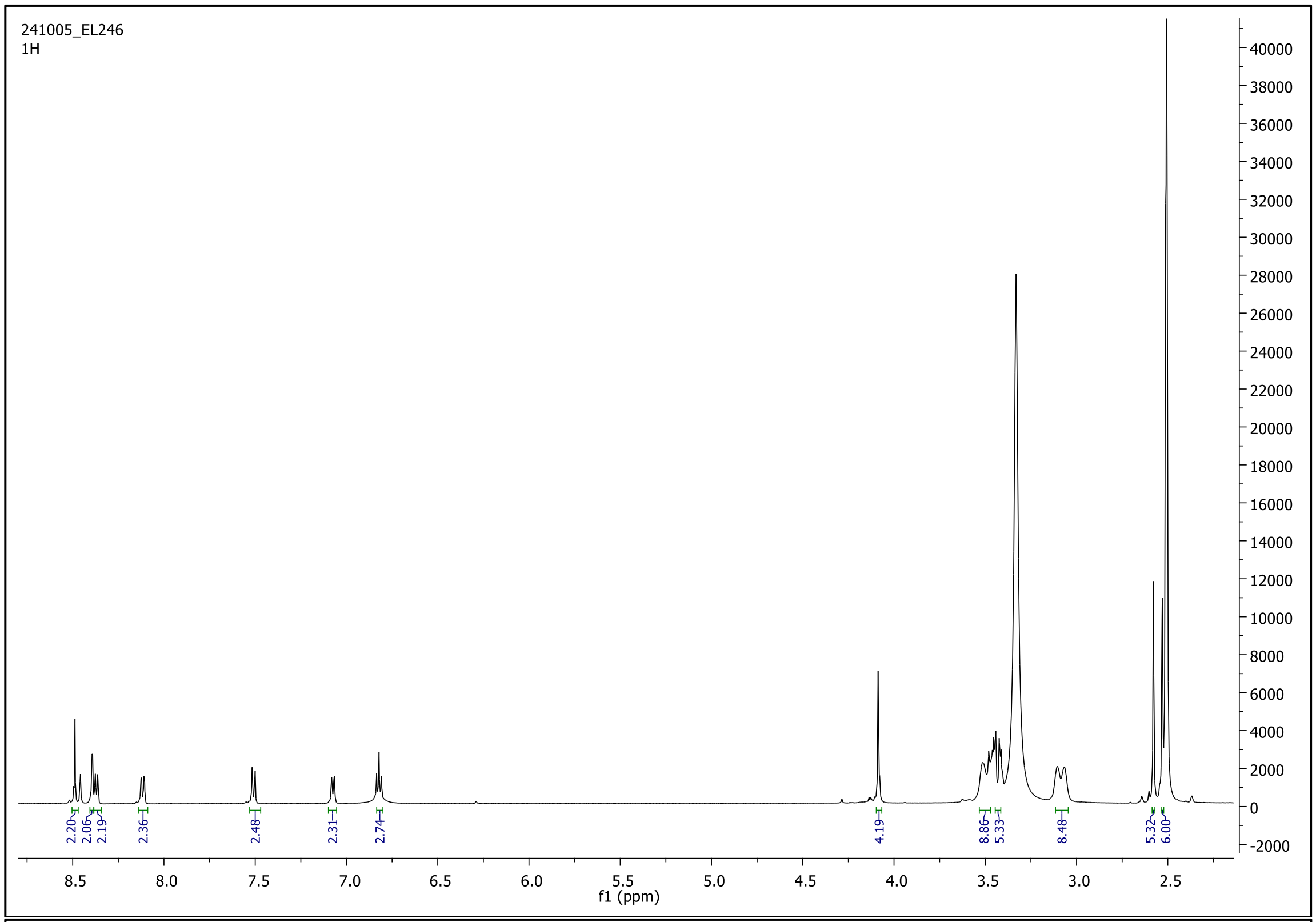
241005_EL246
1H
2.20
2.06
2.19
2.36
2.48
2.31
2.74
4.19
8.86
5.33
8.48
5.32
6.00
f1 (ppm)

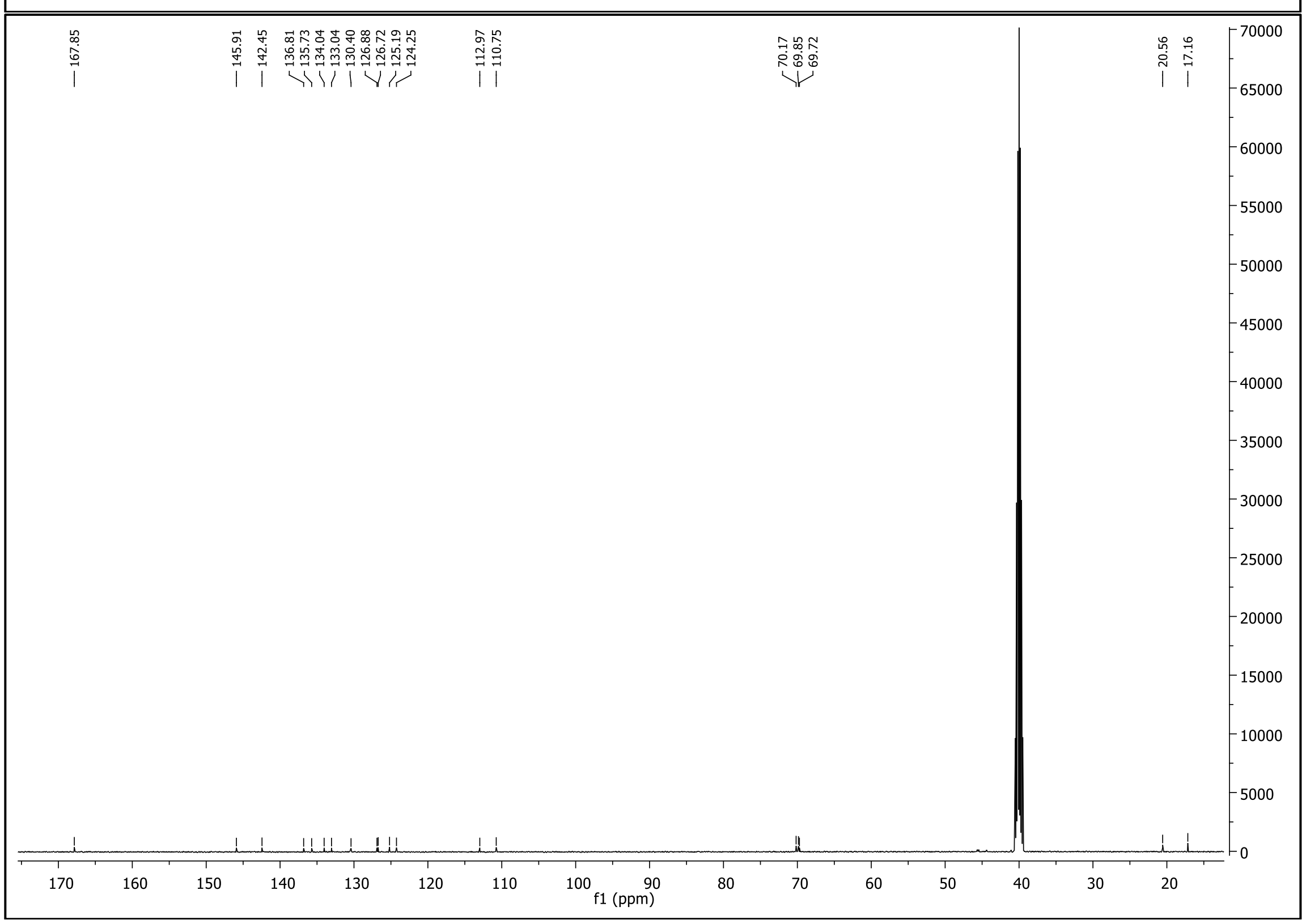
167.85
145.91
142.45
136.81
135.73
134.04
133.04
130.40
126.88
126.72
125.19
124.25
112.97
110.75
70.17
69.85
69.72
20.56
17.16
f1 (ppm)

EL245:

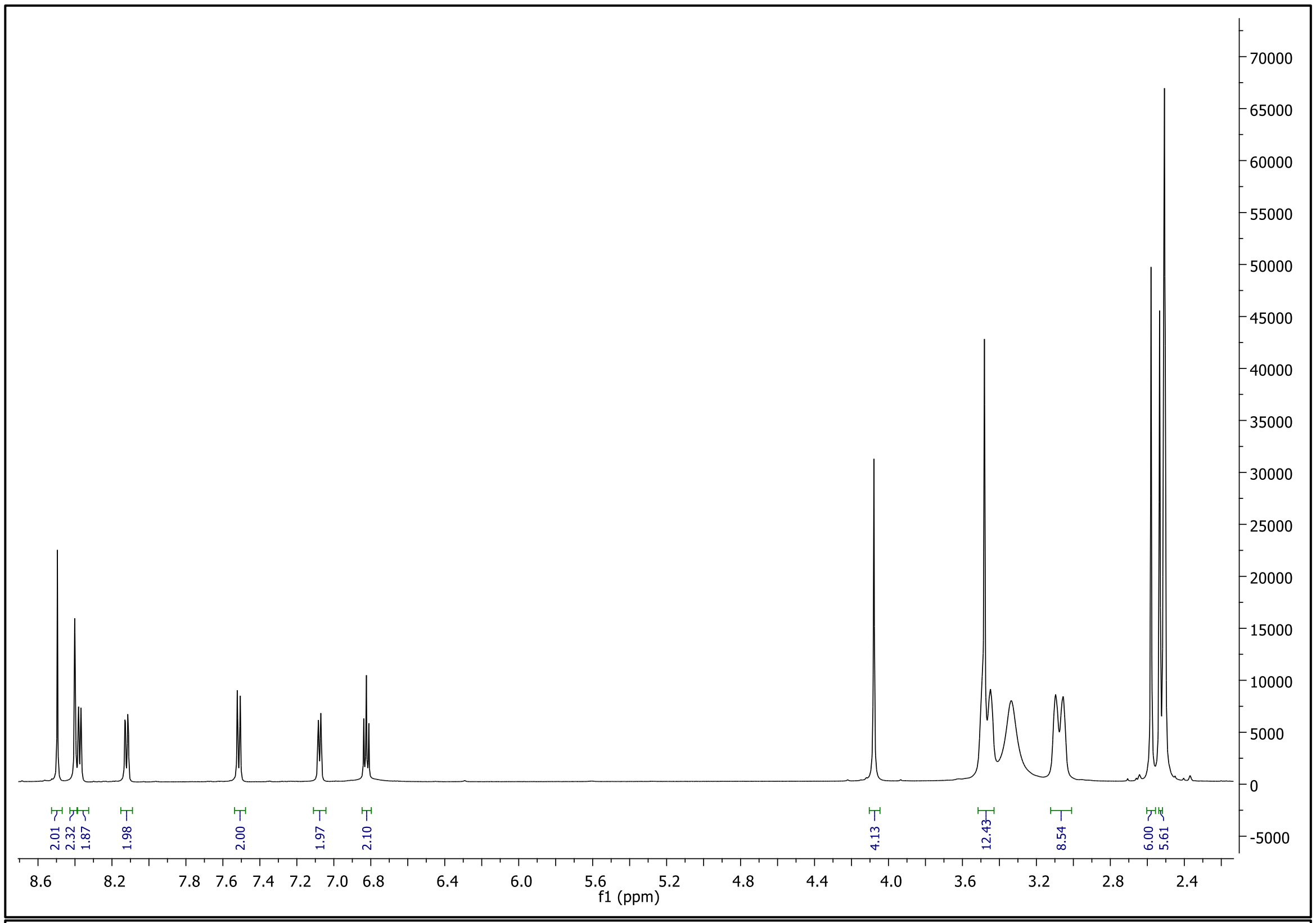

2.01
2.32
1.87
1.98
2.00
1.97
2.10
4.13
12.43
8.54
6.00
5.61
f1 (ppm)

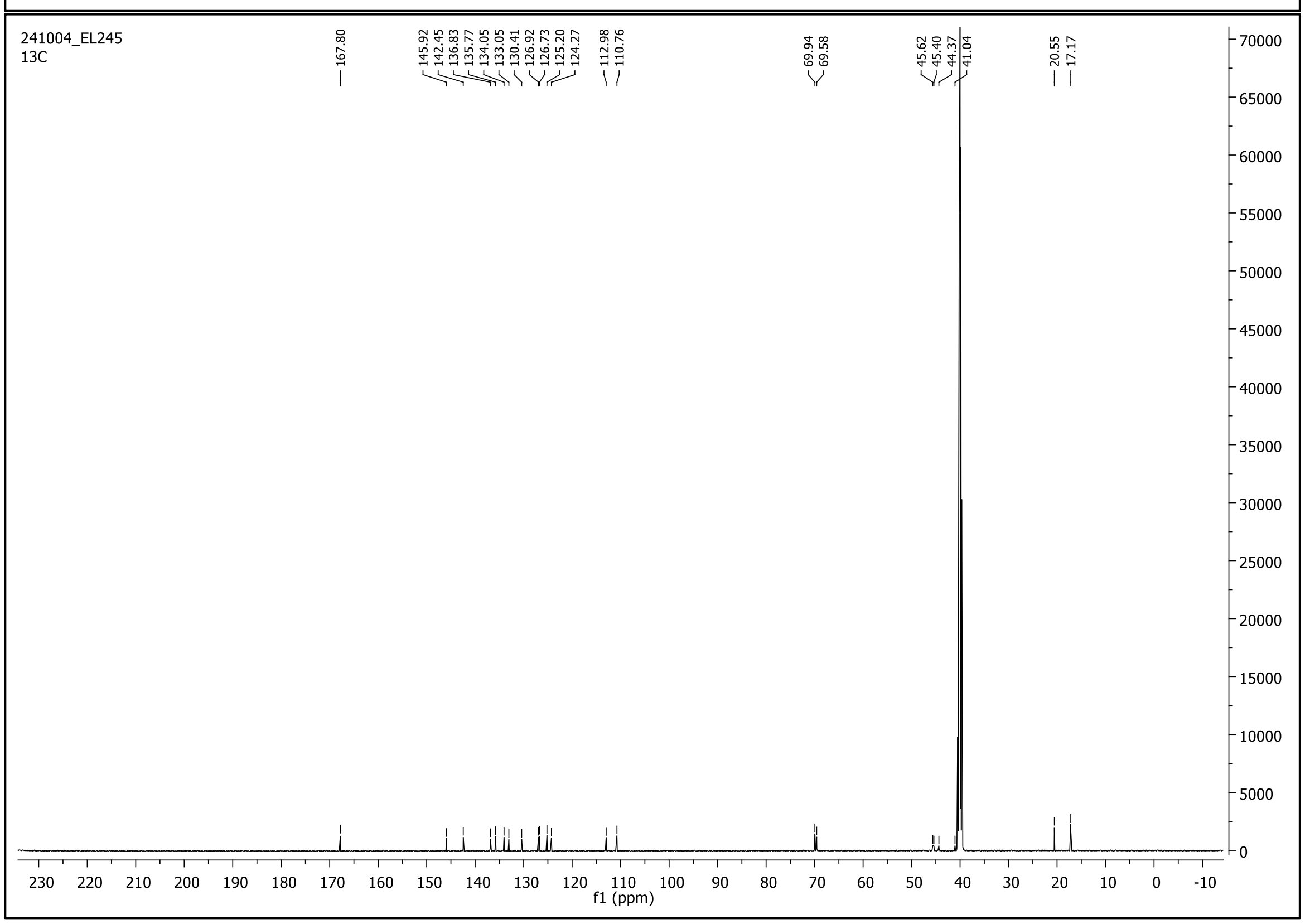

241004_EL245
13C
167.80
145.92
142.45
136.83
135.77
134.05
133.05
130.41
126.92
126.73
125.20
124.27
112.98
110.76
69.94
69.58
45.62
45.40
44.37
41.04
20.55
17.17
f1 (ppm)

EL244:

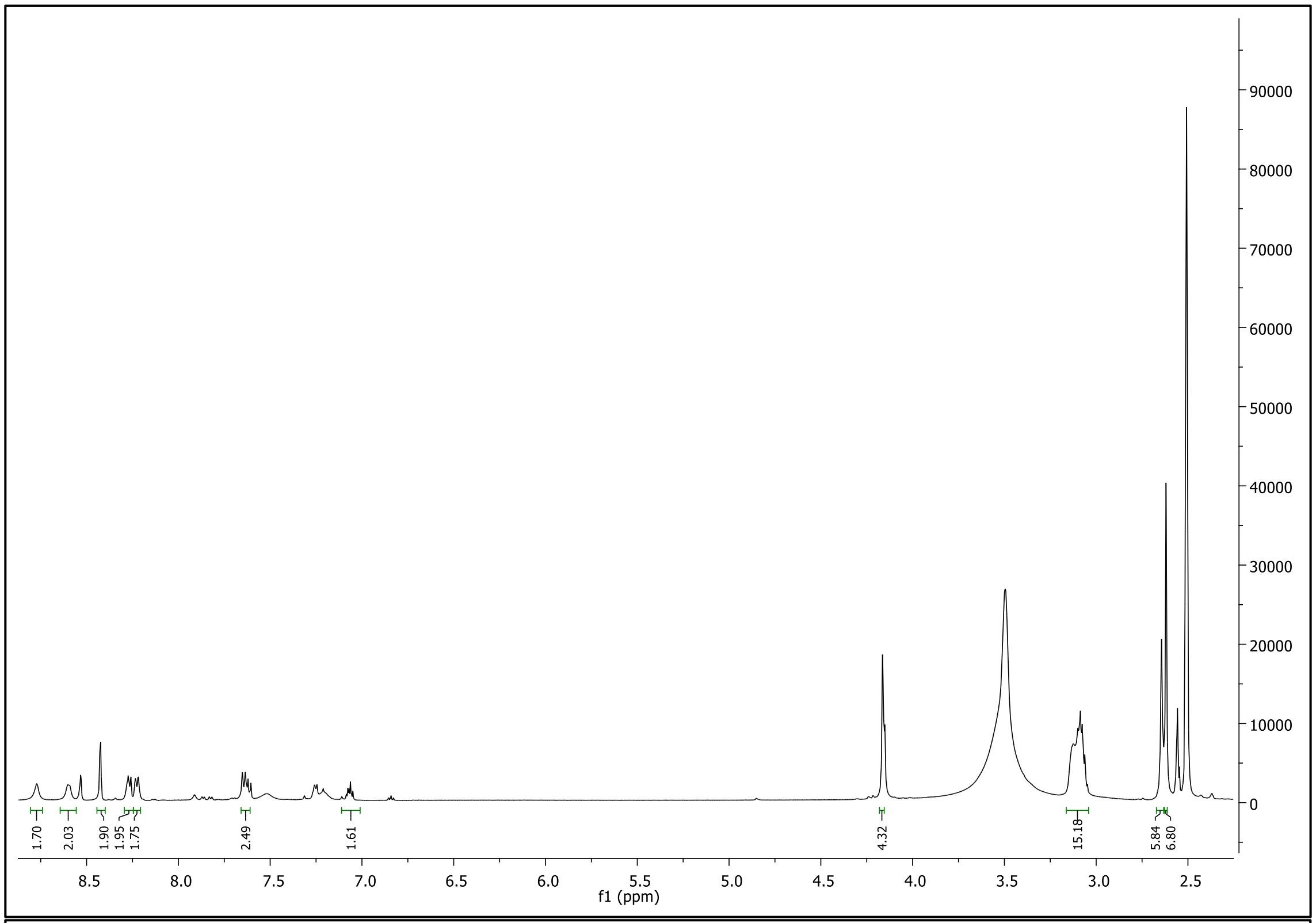

1.70
2.03
1.90
1.95
1.75
2.49
1.61
4.32
15.18
5.84
6.80
f1 (ppm)

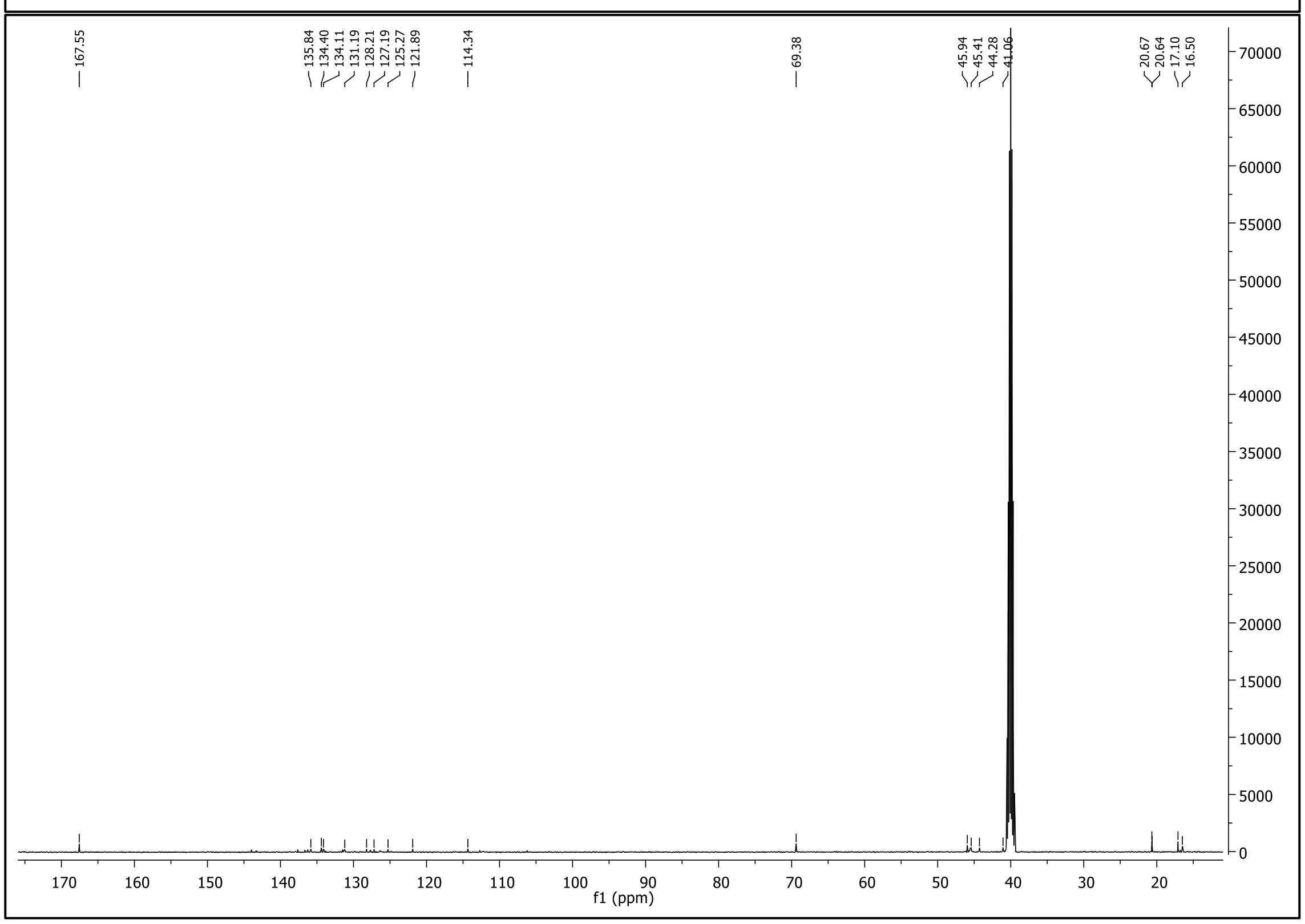

167.55
135.84
134.40
134.11
131.19
128.21
127.19
125.27
121.89
114.34
69.38
45.94
45.41
44.28
41.06
20.67
20.64
17.10
16.50
f1 (ppm)

EL256:

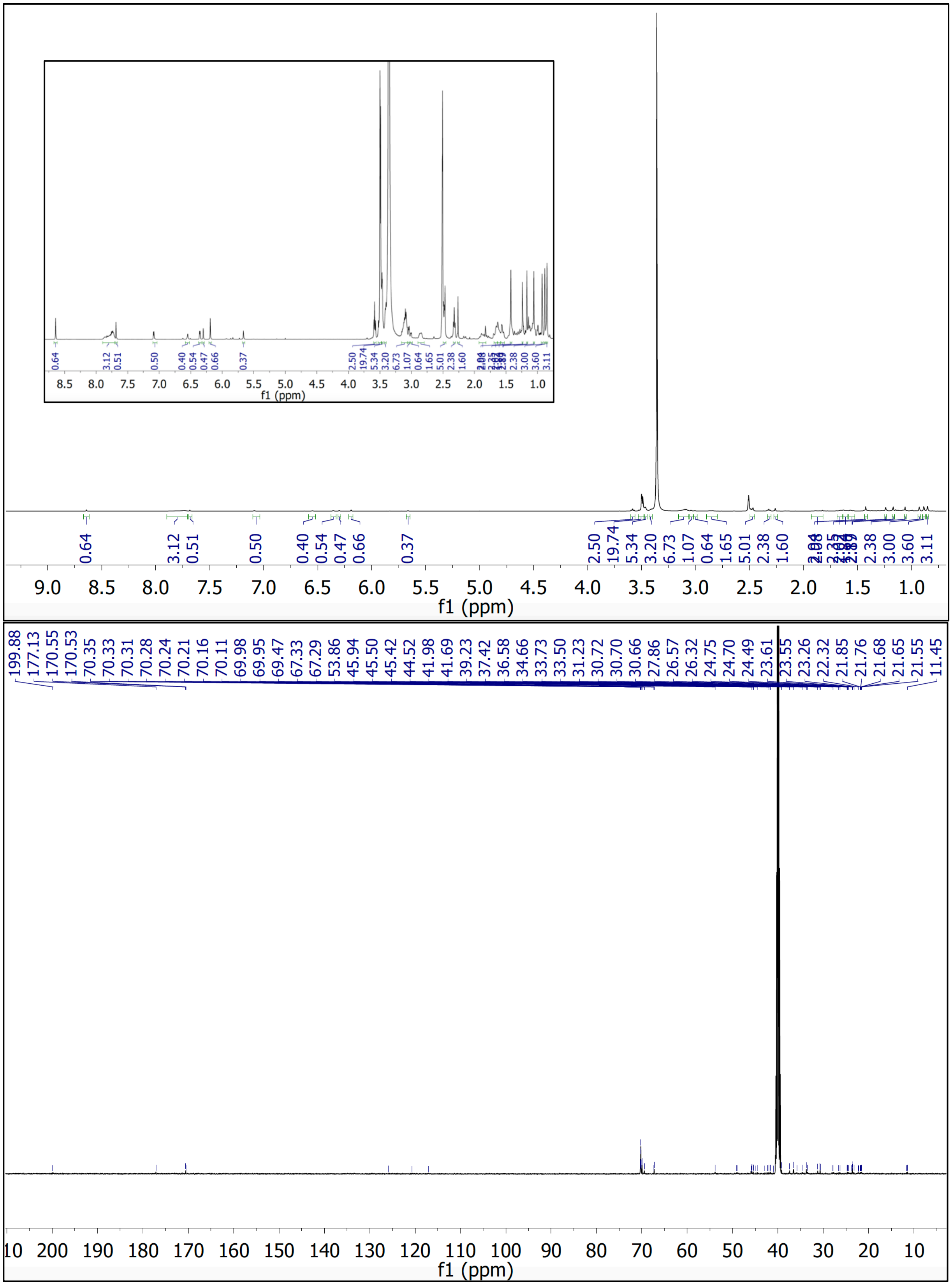

0.64
3.12
0.51
0.50
0.40
0.54
0.47
0.66
0.37
2.50
19.74
5.34
3.20
6.73
1.07
0.64
1.65
5.01
2.38
1.60
2.38
3.00
3.60
3.11
9.0 8.5 8.0 7.5 7.0 6.5 6.0 5.5 5.0 4.5 4.0 3.5 3.0 2.5 2.0 1.5 1.0
f1 (ppm)
199.88
177.13
170.55
170.53
70.35
70.33
70.31
70.28
70.24
70.21
70.16
70.11
69.98
69.95
69.47
67.33
67.29
53.86
45.94
45.50
45.42
44.52
41.98
41.69
39.23
37.42
36.58
34.66
33.73
33.50
31.23
30.72
30.70
30.66
27.86
26.57
26.32
24.75
24.70
24.49
23.61
23.55
23.26
22.32
21.85
21.76
21.68
21.65
21.55
11.45
10 200 190 180 170 160 150 140 130 120 110 100 90 80 70 60 50 40 30 20 10
f1 (ppm)

D. Uncropped gels/blots from supplementary figures 1, 2, 7.

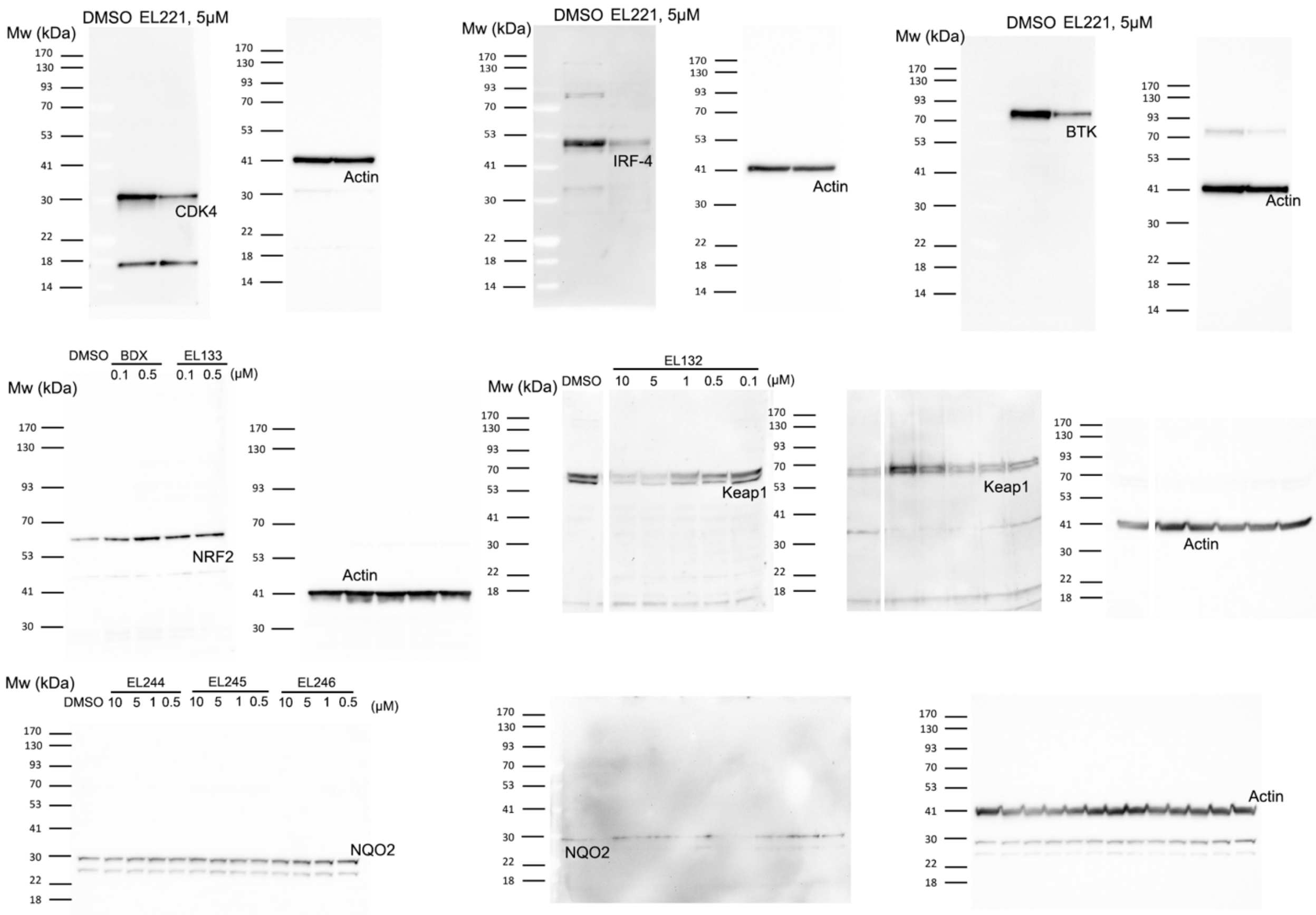

Supplement: Supplementary file 1 [file 41589_2026_2141_MOESM1_ESM.pdf]
