# Supplementary material for: A pharmacological modality to sequester homomeric proteins
Source: Nat Chem Biol. 2026 Feb 5;22(9):1414–24. doi: 10.1038/s41589-026-02141-0 (PMC13503280; doi:10.1038/s41589-026-02141-0)
Supplement: Supplementary file 3 [file 41589_2026_2141_MOESM7_ESM.pdf]

Source data
Main figures and extended data figures- uncropped gels

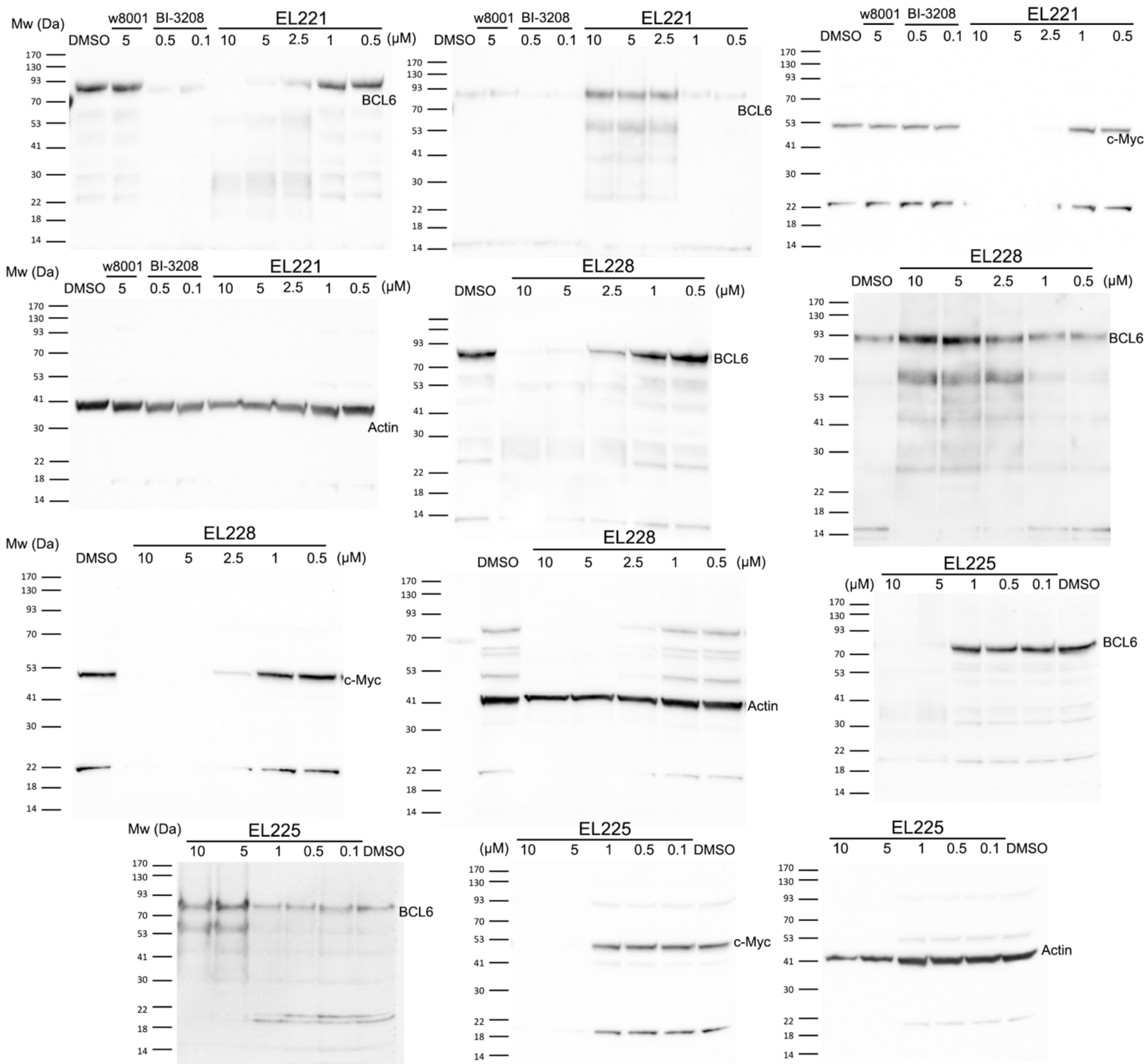

Full gels presented in Fig. 1 of the main text.

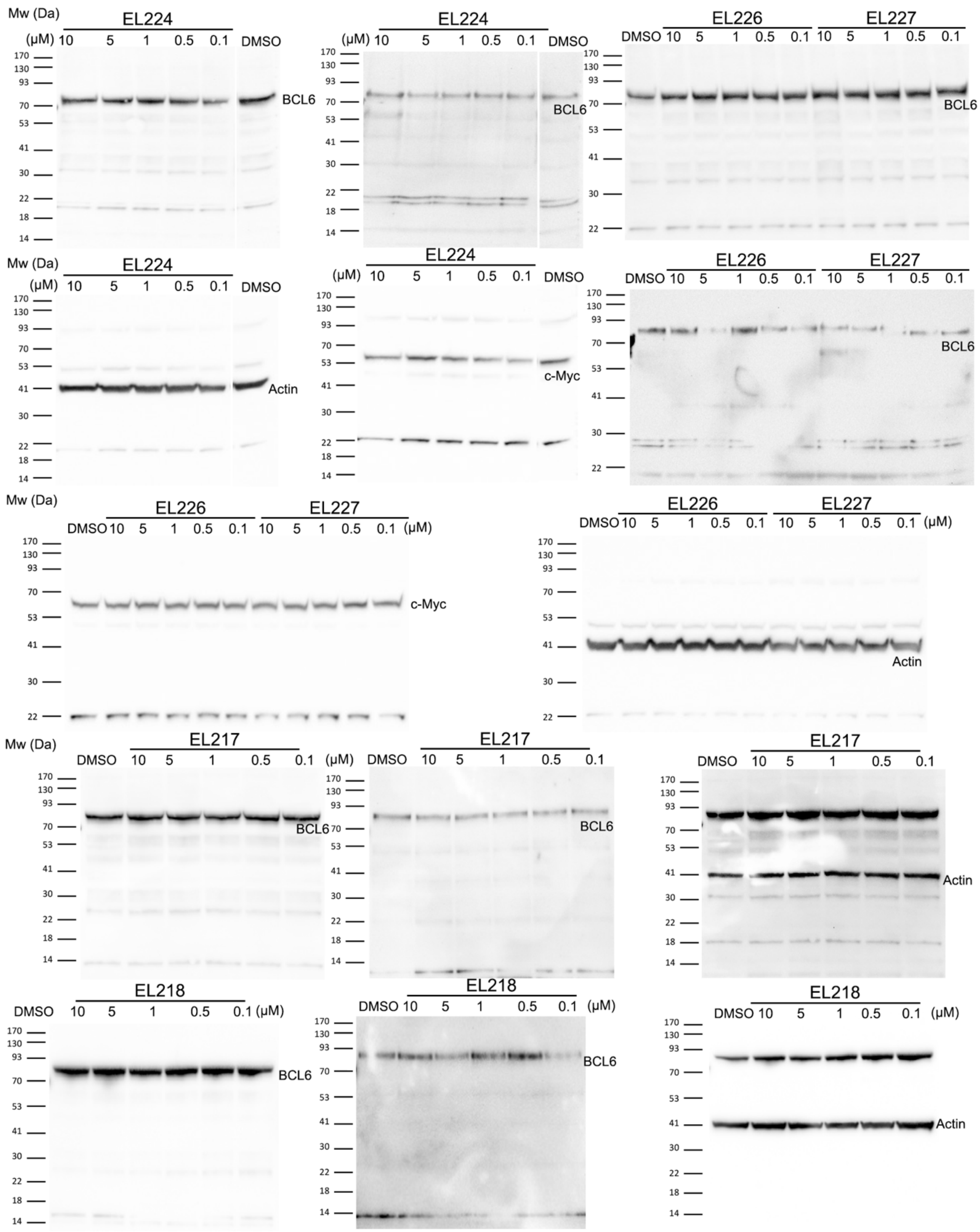

Full gels presented in Extended Data Fig. 1

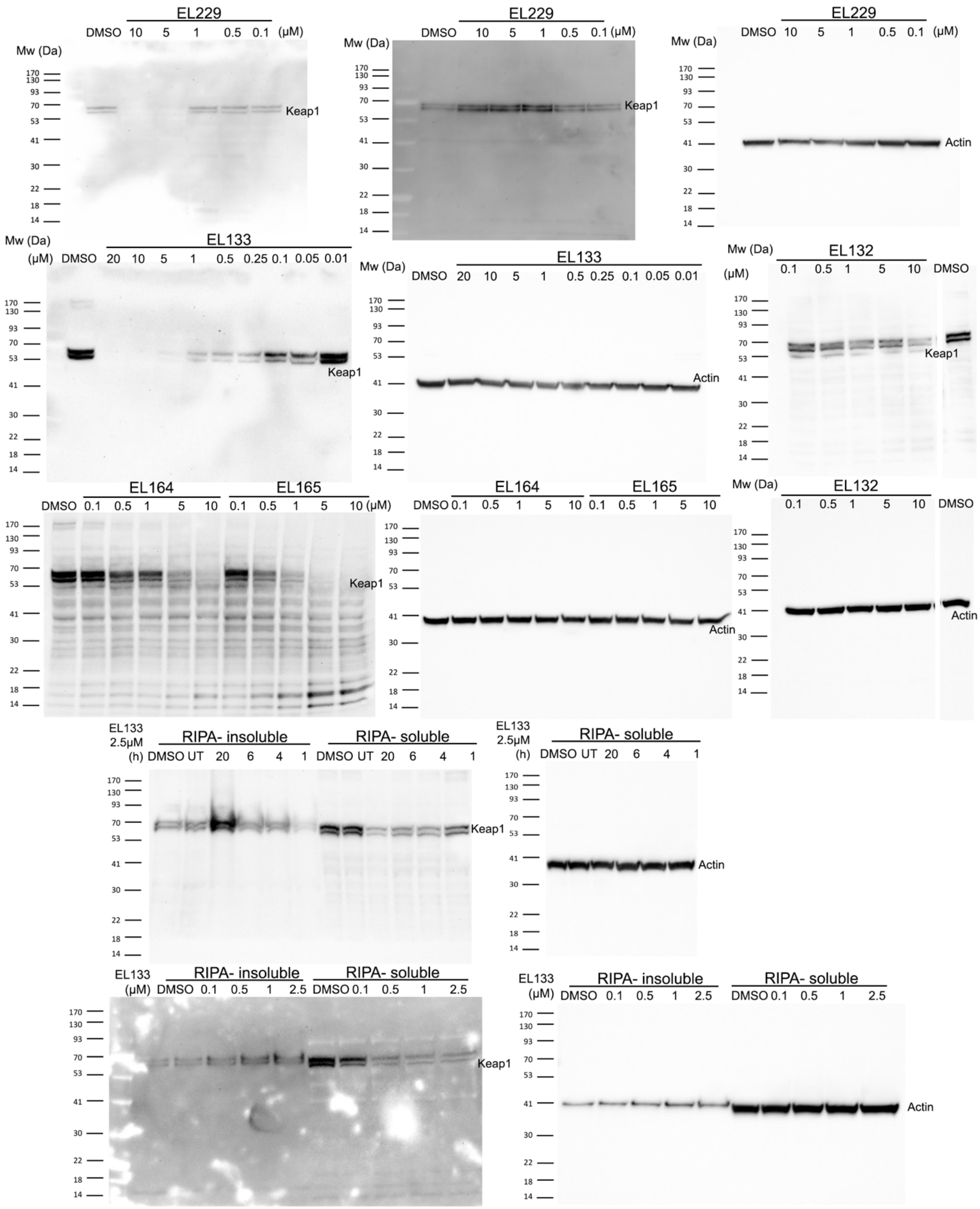

Full gels presented in Fig. 2 and Extended Data Fig. 2 of the main text.

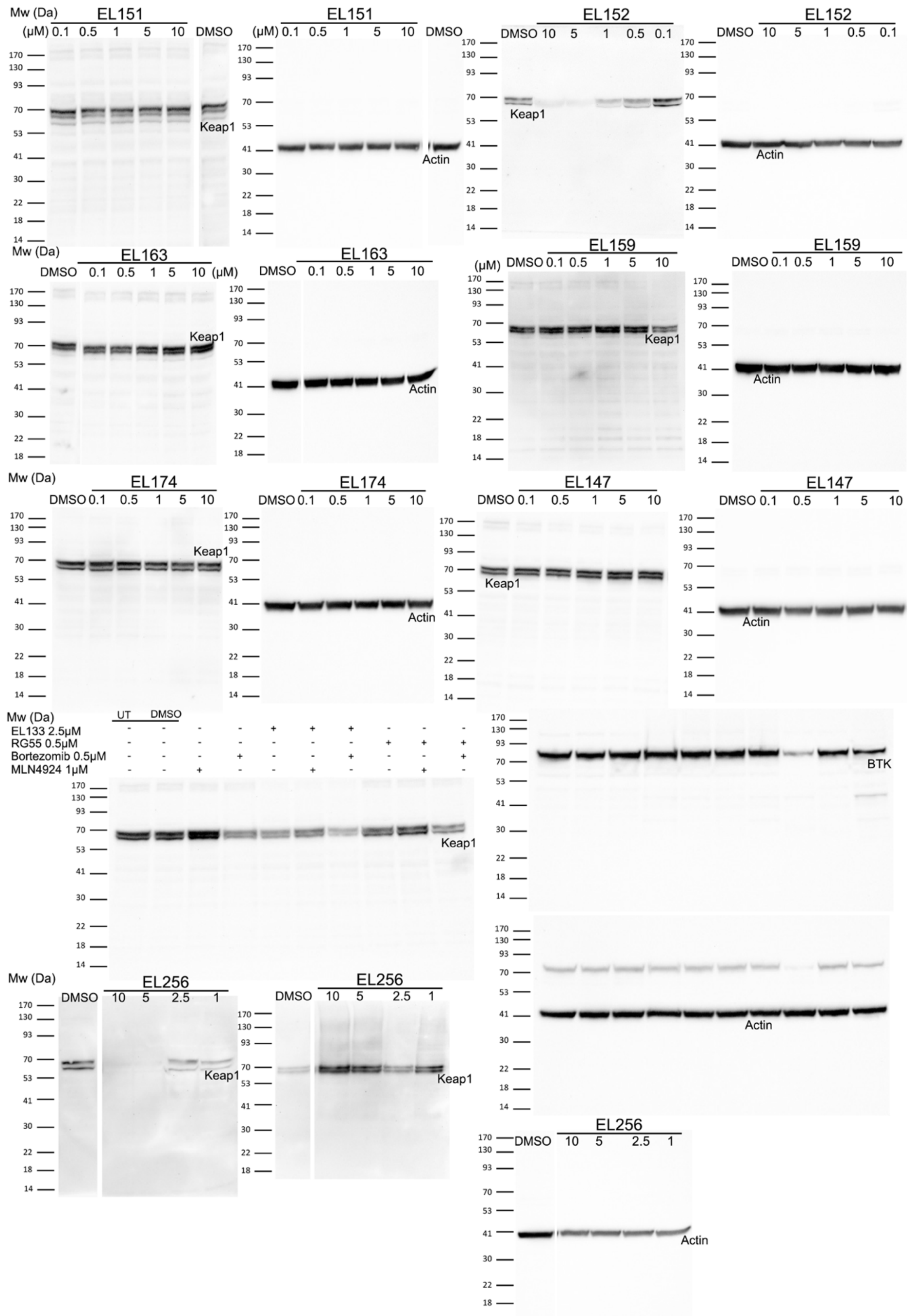

Full gels presented in Extended Data Fig. 2

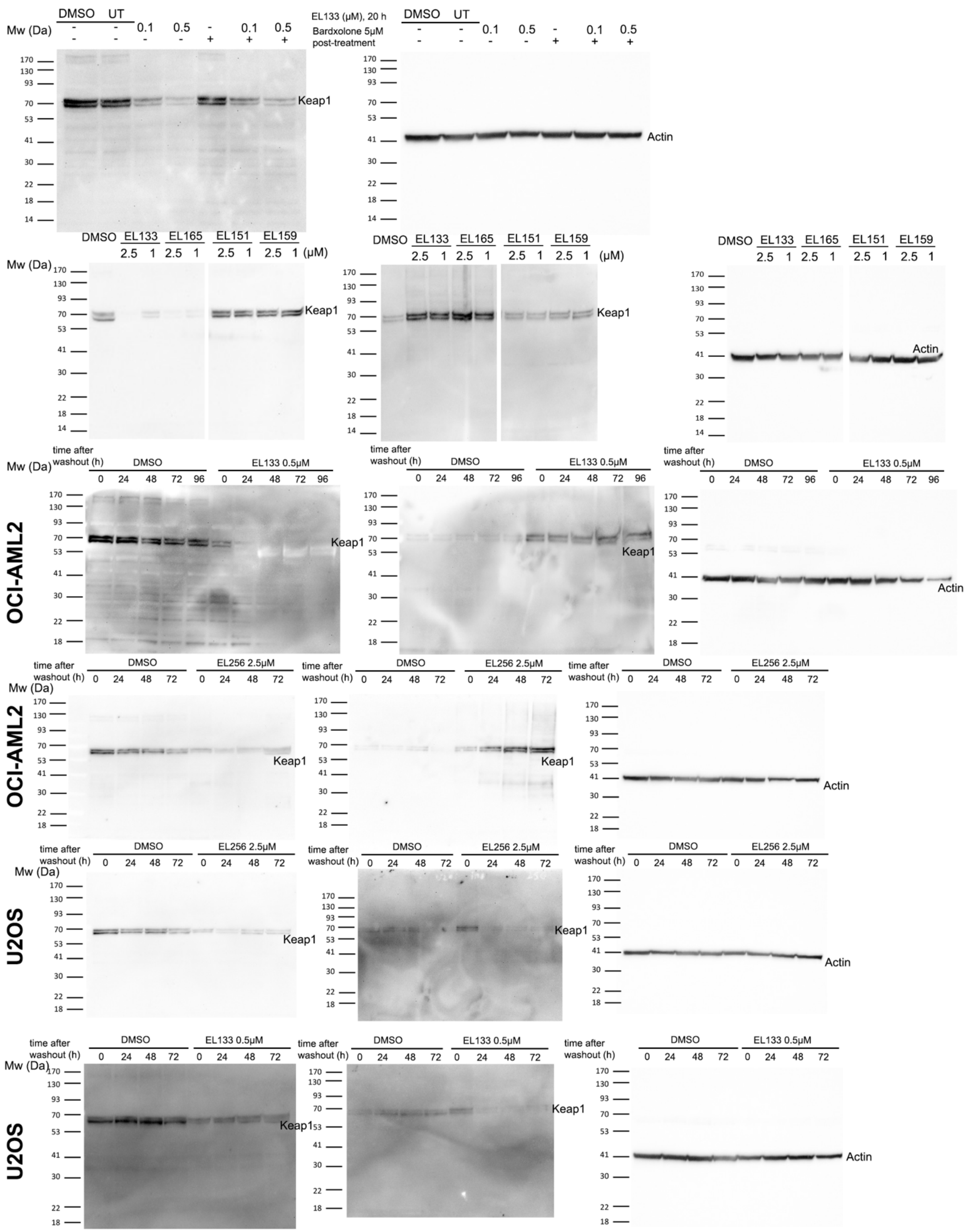

Full gels presented in Fig. 4 And Extended Data Fig. 3.

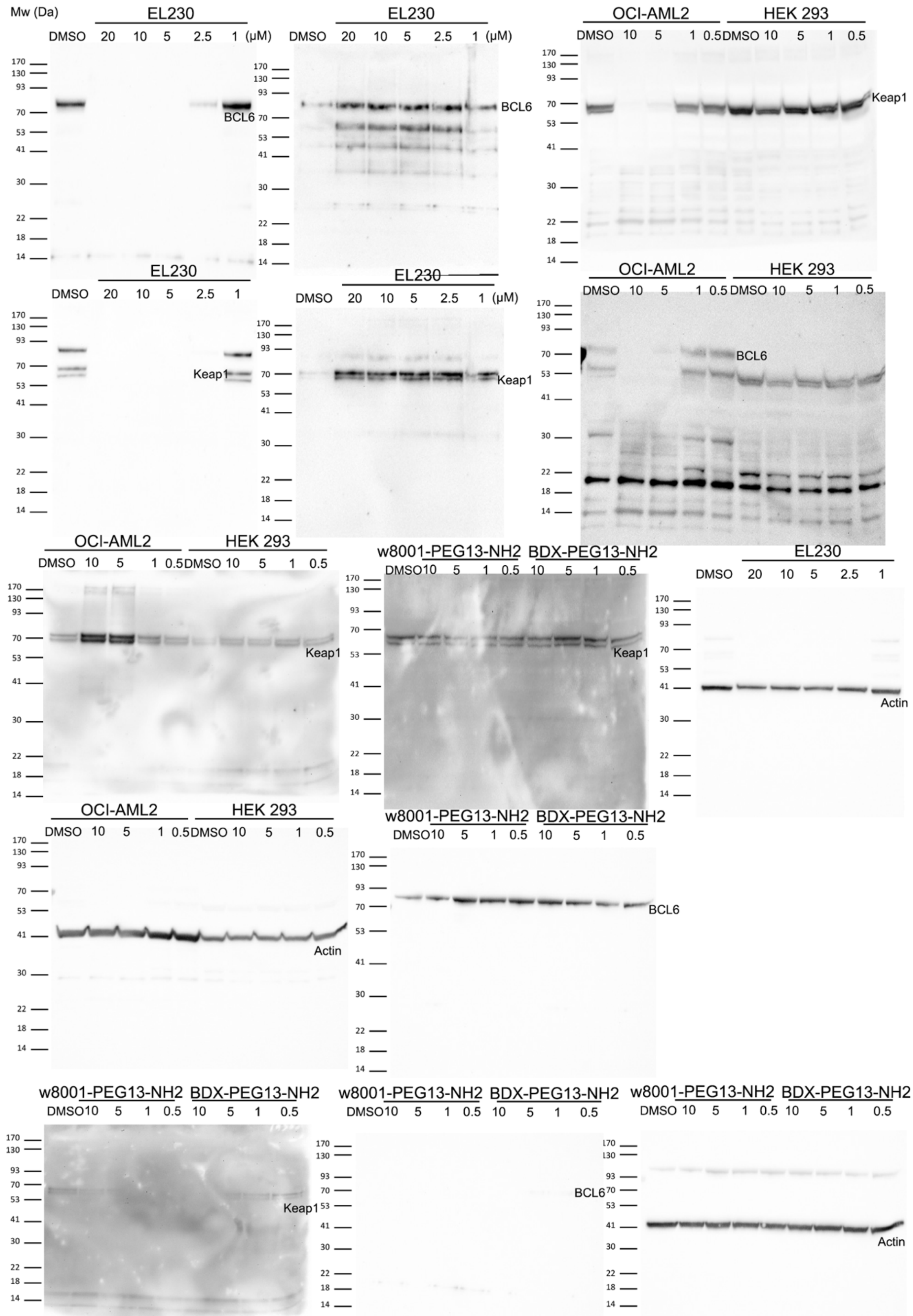

Full gels presented in Fig. 5
